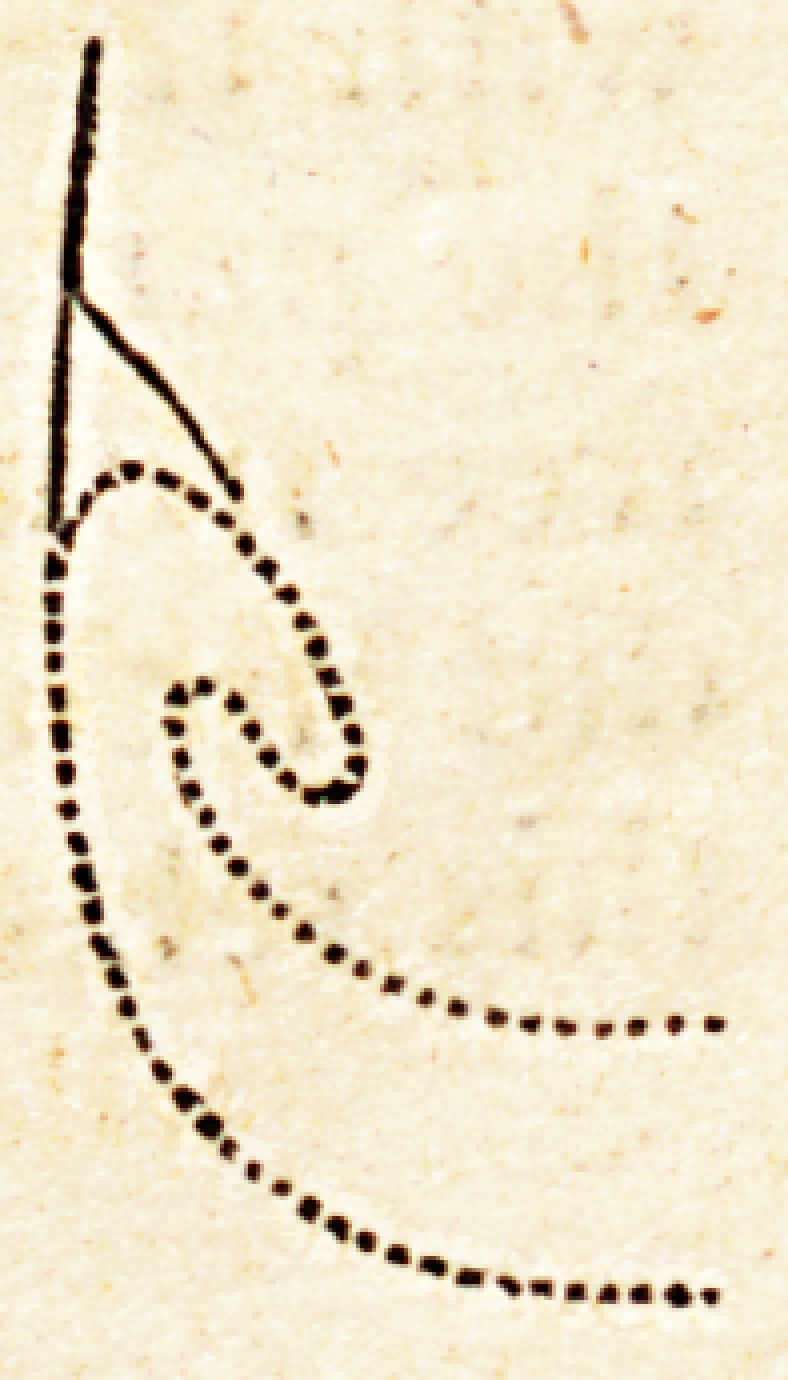# Historical Retrospect for July, 1824

**Published:** 1824-07

**Authors:** 


THE LONDON

### Medical and Physical Journal.

1 OF VOL. LII.] JULY, 1824. [NO. 305.

For many fortunate discoveries in medicine, and for the detection of numerous errors, the world is indebted to the rapid circulation of Monthly Journals; and there never existed any work, to which the Faculty, in Europe and America, were under deeper obligations, than to the Medical and Physical Journal of London, now forming a long, but an invaluable, series.—RUSH.

### HISTORICAL RETROSPECT

FOR JULY, 1824.

### ANATOMY (NATURAL) AND PHYSIOLOGY.

ALTHOUGH it is not easy to draw an exact line of demarcation between some parts of minute anatomy and physiology, yet we shall endeavour, in the first place, to speak of matters relating purely to the disposition of parts, and afterwards proceed to those which involve an inquiry into functions.

The subject of Human Anatomy has been too long and too assiduously cultivated, to have left much for the present generation to accomplish in the way of discovery; and whatever improvements take place consist in new descriptions, new plates, or new arrangements, rather than in the development of any natural part or structure not already known. Of late years, indeed, anatomists, despairing of being able to find new muscles, nerves, or arteries, have devoted themselves to what may be termed the unravelling of textures; and the fibres of every organ (for example, of the heart,) have been followed with most praise worthy patience, and every turn and bending of the most minute muscular thread have been described with the utmost precision; nerves have been traced until lost to the microscope; and arteries have received names, even to the "ramusculi ramusculorum." One great advantage is possessed by those who seek for discoveries of this kind,—we mean the extreme difficulty of detecting or proving their inaccuracy; so that, when a description of some very minute piece of anatomy has once been given, it generally passes current for a considerable time, as not one in a thousand has either the opportunity or the patience to repeat the investigation. Nevertheless, some exceptions to this general barrenness of discovery are occasionally to be met with, one of which we formerly alluded to, in the detection of a new muscle in the orbit by Dr. HORNER, of Pennsylvania; and we shall now present our readers with the account which he himself gives of it in an anatomical work recently published.*

* *Lessons in Practical Anatomy, for the Use of Dissectors.* By W. E. HORNER, M.D. Pennsylvania.

"At the internal canthus of the orbit is a small muscle belonging to the internal commissure of the eyelids, which has not been observed before, or is omitted in the description of the part. That it does not belong to either of the above, [obliquus superior et inferior,] or to the orbicularis palpebrarum, a reference to every minute account of them, given by the most eminent anatomists, will prove.

"This muscle is about three lines broad, and six lines long, arising from the posterior flat surface of the os unguis, near its junction with the os ethmoides, and passes forwards and outwards, lying on the posterior face of the lachrymal ducts. As it approaches the commissure of the lids, it splits into two parts, nearly equal, each of which is appropriated to a duct, and inserted along its course, almost to the punctum lachrymale.

"To get a distinct view of it, the eyelids must be separated from the eye, and turned over the nose, leaving the tendinous attachment of the orbicularis and ciliaris muscles. The valva semilunaris is brought into sight by this process, which must be dissected away, and also the fat and cellular membrane underneath it. The muscle is now seen, and, by passing bristles through the lachrymal ducts, its connexion with them is rendered evident; at the same time we get a good idea of its size, origin, and insertion. While making this inspection, by turning the muscle somewhat aside, we shall be rendered sensible of another fact of some importance,—that the attachment of the inner commissure of the eyelids to the canthus of the orbit is imperfectly described, even by anatomists of much minuteness in their accounts. It is attributed exclusively to the tendon of the orbicularis muscle; so much so, that, in the operation for fistula lachrymalis, we are enjoined not to cut through the tendon, least a puckering of the eyelids be produced by their line of extension being destroyed. The fact, on the contrary, is, that a ligamentous matter behind this tendon passes between the internal extremity of the posterior flat surface of the os unguis; so that, admitting the tendon of the orbicularis to be cut through, this ligament, assisted by the little muscle described, would prevent the dreaded deformity. The internal extremity of this posterior ligament is at least half an inch from the insertion of the orbicularis tendon into the usual process, and it brings into a curve commonly seen at their junction. The lachrymal ducts involved in this posterior ligament, passing along it into the sac, instead of going along the edges of the commissure, as commonly described, just under the skin, must influence considerably the position of the puncta lachrymalia, by drawing them towards the ball of the eye, and keeping them in close contact with it: it is therefore a very efficient means for regulating, so far, the lachrymal passages, and for securing the course of the tears. I am indebted to Dr. Physick for a further suggestion in regard to its other uses, which appears highly probable. In cases of extreme emaciation, it is well known that the adipose matter around the ball of the eye is more or less absorbed, causing it to sink deeper into the orbit, and consequently to retire somewhat from the lids. The effect of the muscle is to draw the lids backwards, and to keep them applied on the ball. Again, in the elevation of the upper lid, or rather the drawing of it within the orbit by the levator palpebræ, the tendency of the margin of the lid is to leave the ball; the upper part of the little muscle obviates this tendency. As such appears to be the actions of the parts, I must therefore coincide with him in calling it *tensor tarsi*, a name expressive of its function."

The minute anatomy of the *Ear* has engaged the attention of Dr. RIBES.* In some of his dissections, he had found the lining membrane of the labyrinth of the ear to be moistened with a fluid of a clear and watery appearance. This fluid, although generally existing in such small quantity as merely to lubricate the inside of the labyrinth, is often more considerable, but still not sufficient to fill the cavities. From having observed that the quantity varied, he was induced to examine to what extent this peculiar fluid occupies the cavities of the labyrinth.

In dissections of persons who had been deaf, he found that the humour contained within the labyrinth was sometimes of a yellowish colour, sometimes of a red and bloody tinge. In the fœtus, the fluid was always bloody in appearance, and occupied all the extent of the cavities. At a later period, it was found more transparent, and less in quantity.

In accounting for a serous fluid being secreted by the membranes of the labyrinth, he supposes it to be analogous to that which is often found in the ventricles of the brain, in the pericardium, the pleura, peritoneum, and joints; and he supposes it to increase in quantity after death.

He dissected the ears of oxen and horses recently slaughtered, to estimate the quantity of fluid within the labyrinth, and repeated the experiment of COTUNNUS, of freezing the ear. He always found fluid, but also a considerable space which was empty. Hence he concluded that there must be air in this space; but how, he asks, does that get admitted, since there is no natural communication with the external atmosphere? He does not solve this difficulty. To discover whether there really be air within the labyrinth, he made some rather clumsy experiments: for example, he put the head of a subject in a basin of water, and fixed it with iron rods; he then introduced a pair of forceps into the ear, and drew away the stapes, at the same time perforating the foramen rotundum; he afterwards poured mercury into the canals. In some of his experiments, air was expelled; and hence he thinks he has proved that air exists in the labyrinth, although he admits that part of it may have been generated after death. He concludes that the internal membrane of the labyrinth is lubricated with a thin fluid, and that an aëriform fluid occupies the rest of its cavities.

The next question he investigates is concerning the manner in which this fluid escapes from the vestibule and cochlea. He had at one time paid great attention to the relative situation of the aqueducts, through which the fluid contained in the labyrinth was supposed to be drained off; but it had not occurred to him, till lately, that this fluid, like the fluids of other cavities, might be absorbed, instead of passing through the pores of the bone. This idea he formed from having observed that,

* *Revue Medicale*, December 1823.

in apoplectic patients, these supposed aqueducts were loaded with blood-vessels. Hence he was led to make injections, and to trace the courses of these aqueducts, by which he found that they were for giving passage to arteries and veins: nevertheless, he still persists in calling them *aqueducts,* and describes their course as such with great minuteness, "winding sometimes round the vestibule and cochlea, and sometimes giving subdivisions which lead into the cavities." After all, he comes to the conclusion which our anatomists had long ago formed,—viz. that these were not aqueducts, but the canals for the passage of vessels. The imperfect knowledge which some of the French possess of minute anatomy, is shown by M. Ribes not being aware that, in describing these openings as being larger in the fœtus than in the adult, he is making only a common observation upon the difference of the cancellated structure of the bone at the several periods of life. We may add, that all he has been labouring to show,—viz. that the passages described by Cotunnus were for the transmission of vessels for the supply of the bone, and the parts of the organ of hearing contained within it, was maintained by BRUGNON, in a paper published in the Transactions of the Royal Society of Turin in 1805.

It is remarkable that M. Ribes never once hints at the labours of Dr. MONRO or SCARPA, nor makes mention of their finished engravings made from dissections. Both of these anatomists describe the vestibule, the semicircular canals, and the cochlea, as being almost completely filled with a thickish fluid, on which the pulp of the nerve is distributed.

If M. Ribes believes that air is better adapted for propagating vibration than fluid, he should have discovered within the vestibule some provision similar to the Eustachian tube in the tympanum. But he appears to have forgotten, or to be ignorant of, the most important laws of acoustics. It is also curious that he never once refers to the nerves of hearing as occupying part of the cavities of the labyrinth.

Dr. WEBER, who formerly published a Memoir on the Mutual Relation between the Development of the Head and the Pelvis,* has lately added some additional remarks to what he had formerly said upon the subject. According to his views, the various forms of the head, which are pointed out as characterising different varieties of mankind, are to be found among all nations,—one or other being more or less common among particular races. The principal forms of the head, according to Weber, are the natural, the round, the pyramidal, the oblique, and the square. The same division holds good with regard to the pelvis, that being considered as the natural which is most regular and most generally found. He asserts that the pelvis always presents the same characters with the head, and this not only in the same nation, but in each individual taken separately, whatever may be the sex, age, or the diseases which may have influenced the conformation of the skeleton: in short, in any individual who has, for example, an oblique head, the pelvis will be found equally oblique; and the same relation will be found to exist between their size and proportions. The cranium corresponds to the

* *Journal für Chirurgie und Augenheilkunde.*

great, and the face to the small basin of the pelvis, so that certain lines of these two parts are always in a definite proportion to certain lines of the other. Thus, the width of the head between the cheek bones corresponds to the upper diameter of the small pelvis; and the distance of the nose from the extremity of the chin corresponds to the length of the symphysis pubis. According to this doctrine, as the proportions of the head can easily be ascertained in the living subject, so from them those of the pelvis can be determined; and hence likewise the exact disposition of the uterus, and even the position of the head of the fœtus.—An example is given of a man, forty-one years of age, in whom both the head and pelvis were oblique, being twisted from right to left, in consequence of rickets.*

Turning from man to the lower animals, we find rather more to attract our notice, the field being more extensive, and consequently not quite so minutely examined. Some anatomical facts have been detailed respecting the genital systems of some species, which may prove not uninteresting in a physiological point of view.

On a former occasion we alluded to the discovery made by Dr. Gärtner, of a glandular organ in the uterus of some of the lower animals: we are now enabled to give our readers a more circumstantial account, the author having published an essay on the subject,† detailing the progress of his investigations. He was engaged in examining the lymphatics in the uterus of a cow, when he accidentally observed a duct, or canal, filled with a yellowish fluid: this he was satisfied, from its appearance, could neither be an absorbent nor blood-vessel. Traces of it could be followed to within an inch of the ovary, and downwards to an opening, by which it ended, close to the orifice of the urethra.

His next examination was on the uterus of a sow, three years old. Upon the side of the vagina next the bladder, he felt a hard cylindrical body, which he opened, and, finding it to be a single canal, he dissected it upwards for some inches; when, by degrees, it divided into branches, which ran into a glandular body, bearing a considerable resemblance to the pancreas. About two inches further on, it again became a single canal, of very small diameter, which stretched on to the uterus. He injected quicksilver into this duct, and found that the mercury came out into the vagina by a small aperture, just beneath the opening of the urethra. He states that he has examined many uteri, both in the gravid and unimpregnated state, as well as the uteri of animals which had been deprived of the ovaries; and that he has generally found a canal beginning on each side of the place where the vagina terminates in the horns of the uterus, running through a body of glandular structure in the middle of the vagina, under the sphincter vesicæ muscle, and opening into the vagina close by the orifice of the urethra. Dr. Gärtner succeeded in injecting this canal with quicksilver. Before it is injected, it has a

---

* *Ein neuer Beitrag zur Lehre von der Conformitat des Kopfs und Beckens.*—(Nova Acta Acad. Cæs. Car. Leop. Nat. Cur. 1823.)

† *Anatomisk Beskrivelse over Et ved Nogle Dyr-arters Uterus Undersögt Glandulost Organ.*

whitish firm appearance, and is about as thick as the barrel of a quill; but, after it has been filled, it appears twice as large, and less compact. Upon being cut into, it has the appearance of the vas deferens, though not so firm in its structure. In the cow, this apparatus is somewhat more complicated; the canal on either side of the vagina expanding into a sac, soon after its origin at the mouth of the urethra, and terminating, about an inch from the uterus, in a cul de sac, which communicates by a valvular opening with a duct which begins here. There are numerous follicles filled with a glutinous fluid, and from these cysts a canal runs along the neck of the uterus, and extends to within an inch of the ovary, beyond which it has not been traced.

Dr. Gärtner states that this organ has been mentioned by some of the old writers on anatomy; but we think there can be no doubt of his being entitled to the merit of drawing our attention to a part which has never heretofore been perfectly described,—which certainly had escaped the attention of modern anatomists, and which appears likely to lead to important physiological deductions.

M. Geoffroy St. Hilaire has remarked, that the generative system of the Ornithorhynchus comprehends the two systems proper to birds and the mammalia; and this examination led him to discover the nature and arrangement of the three passages which serve, in birds, for the abdominal discharges. In the Memoire before us,* the author first makes some general remarks on the sexual system of birds, shewing that in these animals the pelvis is widely open in front, turned backwards, and extending beyond the sacral vertebræ, which it embraces; so that the intestinal canal, being deprived of the situation it has in the mammiferous animals, terminates in the fundus of the urinary bladder. Having pointed out the causes which influence the relative position of the organs, he proceeds to distinguish the parts which have been confounded under the general name of *cloaca.* He calls the space comprehended between the two sphincters of the rectum the " rectal vestibule." In some, as the Ichneumon, or Egyptian rat, this interval is considerable; in other animals the two sphincters are blended; but, in the greater number of the mammalia, this space is formed by two anuses widely separated from each other,—one internal and the other external. In the ostrich, in particular, this vestibule is extremely distinct; but in other birds, as hens and canaries, the vestibule and the bladder form one common pouch of great size, in which is found a narrow cylindrical compartment, where the vasa deferentia in the male, and ovarian ducts in the female, end on either side. This canal, being distinctly perceptible in all animals, is called by M. Geoffroy the " uretro-sexual canal." Another compartment in birds is called by him the " bag of the prepuce," (*bourse du prepuce;*) and this is the last of the pouches. Proceeding, therefore, from without inwards, we have the pouch of the prepuce, the uretro-sexual canal, the urinary bladder, and the rectal vestibule, the whole of which collectively constitute what has received

---

* *Considerations generales sur les Organes Sexuelles des Animaux, a grande Respiration et Circulation.*—Memoires du Mus. d'Histoire Nat. 5 cahier.

the common name of cloaca; each of these has its correspondent part in all the mammalia. Under the name of "accessary bag," M. Geoffroy describes a superadded bag, which opens into the bag of the prepuce above, and at the root of the glands of Cowper, serving as their excretory duct.

In the subsequent part of his Memoir, the author proceeds to consider the generative system of other animals; and concludes by remarking, that the application of certain rules always brings us to a sort of imaginary type, to which all the varieties of form and modifications of organization may be referred; this theory being the leading principle which directs the zoological pursuits of this distinguished naturalist.

Professor JACOBSON, whose interesting discoveries with regard to the veins we formerly had occasion to notice, has lately devoted his attention to the anatomy of the supra-renal capsules,* which he has found in all the quadrupeds and birds he has examined, while he has observed them in but very few reptiles; neither has any thing analogous been detected by him in fishes, with very few exceptions, *(raies et squales.)* In birds, these supra-renal glands have two sets of veins,—one which carries blood to them, and another by which it is again removed, in the same way as takes place in the kidneys of these animals. He conjectures that a similar organization may exist in the fœtus of the quadruped at an early period of its existence, and that the supra-renal capsule may undergo some peculiar change, or be arrested in its development.

Some works of value have been published on anatomical subjects, tending to facilitate the study by improved arrangements and comprehensive views: among these, the "Elements" of M. BECLARD deserve to be noticed here.† BICHAT conferred an invaluable obligation on medical science when he directed the attention of its cultivators to the minute structure of individual textures. Gifted with extraordinary talents and an enthusiastic devotion to his profession, he studied every tissue and every organ, and may in fact be said to have created this branch of anatomy; for MALACARNE, who perhaps first established a distinction between different kinds or systems of organs, did comparatively little with regard to their minute structure. Much, however, as we owe to Bichat, his work, it must be confessed, has been left far behind in the progress of modern science; while the enthusiasm of his character led him to the adoption of numerous erroneous opinions, which nevertheless are supported by such a specious, and often eloquent, train of reasoning, as to render them dangerous. Now the object of M. Beclard is to supply the deficiencies of Bichat, and to rectify his errors; presenting us, in short, with an accurate view of the present state of this branch of anatomy.

---

* *Oversigt over det* K. D. *Vid. Selskabs forhandlinger*, 1823.

† *Elemens d'Anatomie General, ou Description de tous les Genres d'Organes qui composent le Corps Humain.* Par P. A. BECLARD D'ANGERS, Professeur d'Anatomie à la Faculté de Medecine de Paris.—Paris, 1823.

In an introduction of some length, the author gives a general outline of comparative anatomy and physiology, particularly with reference to those animals whose organization most resembles that of man. The general structure of the human body is minutely considered, and more attention given to the history of the fluids than is customary in anatomical works, while the description of each tissue is followed by an account of its pathology. There is likewise a chapter on accidental productions,—such as adventitious fluids, concretions, and tissues; and foreign bodies having life, as intestinal worms and parasitic animals. The work is written in a very compressed style, and contains in one volume a distinct account of many important subjects.

The first volume of an extensive system of Comparative Anatomy* was published a few years ago in Germany, which seems to be little known in this country. It is by the pen of MECKEL, the distinguished professor of anatomy and physiology at the University of Halle. The work commences with the consideration of general anatomy, in which a great deal of fanciful speculation is displayed: thus, we have four chapters containing a general exposition of the laws of formation,—the indication of the essential characters of the animal body,—the development of the first law of formation, called law of variety,—and lastly is considered the second law of formation, called that of analogy or reduction. The characters regarded as proper to give an idea of the animal frame are—1, the external form; 2, the internal structure; 3, the relative situation of parts; 4, the degree of density, or consistence; 5, the number of parts; 6, their volume; 7, their colour. To complete the idea of the animal form, the author adds to the characters we have mentioned, a description of the nature of the organs, their vital properties and their functions. Having pointed out these characters, he passes in review the differences of organization, from the simplest zoophyte up to the most perfect animal; and then examines the different divisions of the animal kingdom, adopted from the time of Aristotle to the present day, terminating with his own, which is as follows:—1, Protozois; 2, Echidermata; 3, Annelides; 4, Insecta; 5, Arachnides; 6, Crustaceæ; 7, Cirrhipedes; 8, Molluscæ; 9, Pisces; 10, Reptilia; 11, Aves; and 12, Mammiferæ.

M. Meckel regards it as of essential importance to found zoological systems on general organization, and not to adopt individual organs as the principle of classification. He combats the opinion of M. Geoffroy, according to whom even insects and the crustaceæ are endowed with skeletons; and refutes the speculations which tend to overthrow the division of animals into vertebrated and non-vertebrated. With regard to the Cephalopodes, which have generally been ranged with the Molluscæ, he places them as an intermediate section between the vertebrated and non-vertebrated animals, on account of the rudiments of a spinal column and bones of the extremities presented by these animals. He attempts to reduce all animal forms, regular and anomalous, to one

* *System der Vergleichenden Anatomie.* Von J. F. MECKEL, &c. &c.—Halle, 1821.

primitive type. We do not know whether or not this work has proceeded further than the volume of which we have given a sketch.

A very elaborate dissertation* has been published by Dr. TREVIRANUS, of Bremen, on the differences between the form, shape, and situation of the various parts of the *brain* in different classes of animals. To this we must refer any of our readers who may be interested in the subject, as it is impossible to give any connected view of the author's minute descriptions, without extracts far more lengthy than our limits admit of.

The *surgical anatomy of the arteries* has been made the subject of a distinct work by Mr. HARRISON, of Dublin,† in which the descriptions are interwoven with practical and physiological remarks. All the arterial branches, of any importance in a practical point of view, are described, with reference to their coverings, the parts they lie upon, and those which they accompany; while the very minute and superficial ramifications are disregarded. This method seems to us judicious, as it relieves the memory from an unnecessary load; and the work itself would probably have been an useful addition to the dissecting-room, had there not already been so many excellent Manuals of this kind.

We have likewise to notice a translation of the General Anatomy of BICHAT, of which only the first part (including two volumes) has yet appeared;‡ and an Introduction to Anatomy and Physiology, by Mr. SANDWITH, in which the descriptions are adapted to the general reader, and the plan of which approaches more to the anatomical part of PALEY's Natural Theology than any other work with which we are acquainted.§

Connected with the subject of anatomy, it may not be irrelevant to mention here that a very favourable account is given of what are called *anatomical imitations*, executed with great zeal and perseverance by M. AUZOUX. These pieces of machinery are so contrived that all the surfaces can be laid bare, as in dissection, from the most superficial parts to those most deeply seated. Each muscle and each organ can be separately removed, and again replaced; each vessel and nerve may be traced from its origin to its minute ramifications; and openings made in the viscera allow their internal structure to be studied. All the characters on which anatomical knowledge depend, as situation, extent,

* *Sur les Differences qui existent relativement a la Forme et a la Situation des Parties du Cerveau, dans les diverses Classes du Regne Animal.* Par le Docteur G. R. TREVIRANUS, Professeur a Breme.—Journal Complementaire, Mai 1824.

† *The Surgical Anatomy of the Arteries of the Human Body, designed for the Use of Students in the Dissecting-room.* By ROBERT HARRISON, A.B. T.C.D. &c.—Dublin, 1824.

‡ *General Anatomy, applied to Physiology and the Practice of Medicine, by* X. BICHAT. *Translated from the last French Edition, by* CONSTANT COFFYN. *Revised and corrected by* GEORGE CALVERT, Esq. &c.—London, 1824.

§ *An Introduction to Anatomy and Physiology, for the Use of Medical Students, &c.* By THOMAS SANDWITH, Surgeon.—London, 1824.

form, attachments, direction, colour, the relations of muscles, the origin, course, and distribution of blood-vessels and nerves, and the disposition of the viscera, are all said to be executed with great fidelity. M. Auzoux is said to employ, in the construction of these machines, a sort of paste which combines solidity with elasticity, which enables him to represent parts which are extremely delicate, as well as those which are of large size,—which resists time and use, and is not liable to be attacked by insects. It is capable of being run into moulds, by which any number of casts may be obtained.*

In the department of PHYSIOLOGY, we find little on which to congratulate ourselves. It would be unreasonable, indeed, to expect that each short period which intervenes between these Historical Retrospects should give birth to any brilliant discovery; but what we lament is, to perceive the danger which physiology at present runs of being brought into utter disrepute, from the manner in which it is cultivated by our continental neighbours, who seem unable to discover that the multiplication of experiments, without some rational object, can never contribute to the advancement of science. Too many of these which we find recorded in the foreign Journals relate to matters of mere curiosity, while others concern facts which we know sufficiently well without any fresh illustration. But the French will not believe that we see with our eyes, or hear with our ears, unless it be proved by experiment. Thus a rage for experiments is the prevailing mania, and every youth who would acquire a name gets him a supply of dogs, cats, rabbits, and guinea pigs, in order to ascertain—no matter what. "Voici un chien, qu'est ce qu'il faut faire." Accident or the whim of the moment seems often to dictate the particular cruelty to which the animal is to be subjected, and the *experiments* are forthwith detailed with all possible minuteness of description, and all the affectation of scientific precision. The next step towards becoming a physiologist of repute is to lay this account before the Institute, a committee of which is appointed to report upon it, and accordingly do report, that it is very clever and very learned; that the author is an ornament to science and an honour to France. Those who are acquainted with the aspect which experimental physiology has lately assumed in that country, will be sensible that this picture is not too highly coloured: indeed, it is quite extraordinary to see with what facility the approbation of various learned bodies in Paris has recently been bestowed upon dissertations the most directly contradictory of each other. We beg not to be misunderstood: it is not to well-directed experiments, instituted to ascertain important objects, that we object, but only to such as we have above alluded to. Viewing the matter in this light, our readers will excuse us if we decline to recapitulate *all* the discoveries recently made by slicing away portions of the brain and cerebellum,—the mode of investigating the functions of the nervous system at present in fashion. Such, however, as appear worthy of attention we shall relate.

Since our last Historical Retrospect, we find many new volunteers in the

---

* *Journal Universal des Sciences Medicales* 95 cahier.

ranks of those who have devoted their attention to the nervous system, prosecuting and extending the experiments on different portions of the brain, after the manner particularly practised by ROLANDO in 1809, and recently revived in France by M. FLOURENS; an account of whose investigations has been given in the preceding volumes of this Journal. One of these gentlemen seems to have surpassed the others in the refinement of his speculations: we allude to M. BAILLY; and, in giving a sketch of his views, they may be taken as a general illustration of the manner in which the subject is at present cultivated.

The principal results of his Essay are certain anatomical facts and general conclusions therefrom, relative to the organization and functions of the nervous system. Among the former we find a particular account of the tubercula quadrigemina in fishes. These organs, which have hitherto been regarded as belonging to one individual function, are composed of two systems entirely distinct from each other in the direction of their fibres, in their relation to the neighbouring parts, in their distribution, their commissures, and functions. One of these systems is formed by transverse fibres, originating in general from the striated body, which is itself the termination of one of the bands of the spinal cord. These fibres pursue their course upwards and inwards, and all meet on the median line with those of the opposite side, where they communicate by means of other transverse bands, constituting commissures. The other system is external to the preceding, and formed of the fibres of the optic nerve, which dilate into a nervous band, covering the inner part described above. The fibres of this external plate are in general longitudinally oblique. In order to attain their destination, they run downwards, crossing the fibres of the internal band, and go to form the optic nerve. The commissure of this part is found, in the greater number of animals, behind the optic nerve, beneath which it is sometimes hid. M. Bailly first discovered it in the buffalo, and afterwards in various others. This commissure runs in the inferior class to the posterior part of the tubercula quadrigemina, of which it constitutes in some sort the external and posterior margin: it belongs only to the external, and in no degree to the internal tubercle, which, as already mentioned, has its own peculiar commissure. Thus each tubercle has its commissure, that of the internal being above, and that of the external being situated below. This commissure has served the author with the means of determining the use of a nervous fasciculus, which, although pointed out by various writers, had never been referred to any particular system,—that, namely, which goes from the posterior portion of the tubercula quadrigemina to the corpus geniculatum externum. This, according to M. Bailly, is the termination of the commissure of the optic nerves in the mammalia, and the following are the grounds on which this opinion is founded:—

The tubercula quadrigemina being simple in the inferior classes,—that is, only consisting of one lobe on either side,—are subdivided into many parts, which in the mammalia have received different names: thus the anterior and posterior tubercula quadrigemina, the corpus geniculatum, both external and internal, are only portions of the optic lobe of inferior animals. The commissure of the fibres of the optic nerve, which runs to the posterior part of this lobe in the inferior classes, is visible in the whole of its extent; but, in mammiferous animals, one portion of these optic lobes is situated precisely on the course of this commissure,—viz. the corpus geniculatum externum. The commissure in question is thus interrupted but in appearance only, for it is continued underneath, and at length ends in the fasciculus above mentioned, which runs from the posterior part of the tubercles to the corpus geniculatum. Now, as, in the inferior classes of animals, the commissure of the optic nerves goes to the posterior part of the tubercula quadrigemina,—and as, in the mammalia, it runs to the posterior tubercle,—we have, in this distribution, a new proof that the posterior corpus quadrigeminum is the same organ as the anterior, from which it is only accidentally separated by a furrow, which is not to be found in the inferior animals.

M. Bailly next shows the singular connexion between the olfactory and optic nerves: the former one covered by the hemispheres of the brain, the ventricle of which is, in some measure, a continuation of the cavity of these nerves; the latter, instead of being covered, do themselves cover the expansions analogous to the hemispheres; for he considers the internal division of the tubercula quadrigemina in this light. Another proof of resemblance consists in the presence of a commissure, which these two nerves possess.

The anterior commissure has been described as simple, going to the middle lobes in man, and to the olfactory nerves in other animals. M. Bailly has discovered that a distinction exists between the herbivorous and carnivorous animals; for, in the latter, this commissure is double, one part going to the middle lobes, and the other to the olfactory nerves. The fifth pair has never been described as possessing a commissure: M. Bailly, however, has found one in the lamna *(lamna cornubica)*, and has likewise described a particular conformation of the anterior lobes of the hemispheres in birds, and probably applicable to other animals. These convolutions, being independent of the pyramidal eminences which cross each other at the summit, cannot afford the same phenomenon of impressions communicated to the opposite side.

M. Bailly is of opinion that the vertebral canal contains not only the origin of nerves of sensation and motion, but likewise that there are in it organs analogous to the hemispheres, and which are the seat of volition. He lays down the following propositions:—1. Every segment, every ring, every vertebra, of an animal, contains the same nervous elements throughout the whole length of the animal. 2. In all the vertebræ of the neck and back, there are nerves of sensation, motion, and digestion; and an intellectual system besides, to receive the impressions of these nerves, in order to produce consequent determinations.

In the head, this intellectual system is constituted by the brain. In the vertebral canal, it is imagined by M. Bailly to be composed of *eight* longitudinal cords, which are described by him, and which terminate anteriorly, each having a distinct development. Thus the inferior median cord, which is continuous with the pyramidal eminence, terminates anteriorly in the hemispheres of the brain. The inferior lateral cord terminates in the internal fold of the tubercula quadrigemina; so that, according to M. Bailly, it is a new organ of intellect, and not one of sensation.

The superior lateral cord terminates in the cerebellum. The superior median cord terminates in the lateral parts of the medulla oblongata. This last organ, of which the greatest development takes place in cartilaginous fishes, is recognized by the author as analogous to the cineritious folds existing in the mammalia. Thus, the hemispheres of the brain, the internal fold of the tubercula quadrigemina, the cerebellum, and the lateral convolutions of the medulla, are organs analogous as to their intellectual functions, and must necessarily be exercised in the eight longitudinal cords of the spinal marrow, which are continuations from them.

The swellings which the spinal marrow presents in different points of its length are not participated by the intellectual cords. The spinal nerves have two origins, which embrace the intellectual cords between them. The same disposition occurs in the nerves of the head, which only differ from the other in the superficial changes produced by the development of the intellectual cords of the head. The author shows that the anatomy of the human body, having preceded that of other animals, has thus influenced the names given to different parts, by which, different names have been given to the different roots of the same nerve: thus the third and fourth pairs, in man, issue from the head separately, but in the inferior classes of animals only constitute one pair. So that, if we are to consider as distinct those nerves whose roots do not issue by the same foramen, we ought to regard each vertebral pair as composed of two different nerves, since, in some cartilaginous fishes, each fasciculus of the anterior origin issues by a different hole from those of the posterior.

Each vertebral pair of nerves communicates externally with a ganglion of the great sympathetic. M. Bailly has found the same correspondence between the cerebral nerves and other ganglia, such as the ophthalmic, the spheno-palatin, naso-palatin, &c., which are to the cerebral nerves what the ganglions of the great sympathetic are to those of the spine. Each vertebral pair furnishes nerves of respiration, or communicates with them. The same disposition obtains with respect to the cerebral pairs. The author considers the fourth and sixth pairs as nerves of respiration of the eye. Each vertebral pair is connected, by one of its extremities, with the intellectual system: the same with regard to the cerebral nerves.

Radiated animals have been considered as formed on a different plan from the articulated or vertebrated; the author, however, finds the same radiated disposition in the spinal marrow, which, composed of eight cords circularly disposed, exactly resembles the ganglionic circle of polypi, &c. In these animals the ganglions have a globular form, while in the vertebrated animals they are elongated into cords; the difference being in their form, and not in their nature.

Some experiments upon turtles tend to confirm the opinion that the author has advanced on the functions of the longitudinal cords of the spinal marrow, which he has designated by the name of *intellectual*, to express their analogy with those of the brain. He asserts that experiments agree with anatomy in pointing out that these cords partake with the brain the privilege of being the seat of volition and determination, and having the power to command the movements of consciousness and intention; a property which heretofore has been regarded as belonging exclusively to the brain.*

The Physiological Journal for October contains the result of some researches made by the editor, with regard to *the functions of the corpora striata and tubercula quadrigemina.* MAGENDIE had pointed out, in a preceding paper, that, when the hemispheres of the brain are removed, the animal begins to run with great activity. In prosecuting his experiments further, he found that it was not the loss of the entire mass of the hemispheres which gave rise to this effect, but only the loss of the corpora striata; for, if the two hemispheres, with the corpus callosum and the anterior lobes, be removed from a young rabbit, without touching the corpora striata, this running forwards does not take place, nor is the gait of the animal changed. If only one corpus striatum be removed, the motions remain free, the animal directing them, and stopping at pleasure; but, as soon as both these bodies are cut away, he rushes forwards, as if impelled by some irresistible power. Magendie had formerly stated, that the removal of the optic thalami produced much more considerable injury than the loss of the corpora striata; animals so treated losing even the power of standing. These effects, it now appears, result from a vertical incision behind the optic thalami: in making another behind the tubercula quadrigemina, the members which were previously apart are drawn together; but, to secure this effect, it is necessary that the medullary stratum lying on the basis of the cranium be intersected.

It would appear from these experiments, that the energies connected with motion reside in the white medullary fibres, which radiate from the corpora pyramidalia towards the hemispheres. The sensibility of this part, however, appears to be less than that of the upper medullary stratum; the distinction of the two properties, which is so evident in the spinal cord, may be recognized even in the medulla oblongata.

M. Magendie has long been in the habit of showing, in his Course, that a wound involving both optic thalami occasions loss of sight; and, with a view of ascertaining whether the optic nerves cross, he renders the cornea of one eye opaque, and after a certain time examines the parts within the head. In birds, he has always observed the nerves of the blind eye wasted, and that this wasting extends even to the optic thalamus of the opposite side; the thalamus having shrunk in size, and the nerve lost its medullary matter. Nothing analogous, however, has resulted in mammiferous animals, even at the end of twelve months after the loss of an eye; in such cases, the nerve being wasted only anterior to the crossing. It is said that there exists in the Museum of Comparative Anatomy at Paris, a specimen in which the wasting had extended even to the thalamus of the opposite side, in a horse blind of one eye.†

M. DESMOULINS has likewise given the result of some observations,

---

* *Bulletin des Sciences Medicales.*

† *Journal de Physiologie Exper.* Octobre 1823.

which tend to confirm those of M. Magendie. In a falcon which had suffered from ophthalmia, ending in suppuration, with thickening and opacity of the cornea, both retinæ were smooth, which induced M. Desmoulins to regard the wasting of the optic nerve, and the disappearance of the folds of the retina, as the simultaneous effect of the atrophy caused by the inactive state of the eye.* He enumerates various facts recorded by MORGAGNI, SŒMMERING, WROLIK, and others, tending to prove that the effect of blindness is to produce wasting of the optic nerves and thalami.

It would be alike tedious and useless to recapitulate all that has been advanced with regard to the inquiry which has recently occupied the French almost exclusively,—namely, to determine the function of each portion of the nervous system. According to M. FLOURENS, the hemispheres of the brain are the seat of sensation and volition; the spinal marrow and nerves give rise to motion; the cerebellum governs the motive powers; while the medulla oblongata presides over the operations of instinct. All these positions are founded on experiments supposed to be incontrovertible; nevertheless, they are repeated by others with results entirely different. SERRES maintains, that injury or removal of the corpora striata paralyses the lower extremities, and that the optic thalami command the superior members. MAGENDIE again cuts away the cerebral hemispheres and corpora striata, and the animal, as we have mentioned, runs straight forward by an irresistible impulse.—The cerebellum, too, is the subject of much contrariety. Some (FLOURENS, &c.) tell us that it is the regulator of all motion; others (ROLANDO, &c.) that it is not the regulator, but the actual portion of the system whence proceeds the power of motion; and a third set (FODERA, &c.) request us to believe that it is the seat of sensibility, and especially of sexual desire. In short, the whole subject remains at present a tissue of jarring opinions and contradictory experiments.†

Dr. BELLINGERI, of Turin, lately read to the Royal Society of that city, a Memoir on the *functions of certain nerves, particularly those of the spine*.‡ His experiments were performed on sheep and horses, and the following are the most important results. The posterior roots of the lumbar and sacral nerves communicate the power of extension to the inferior extremities in quadrupeds; the anterior roots of the same nerves, on the contrary, only give rise to the movements of flexion; the posterior roots alone preside over the sense of touch; the white substance of the spinal cord, and nervous filaments arising therefrom, are destined for movement; the grey matter, on the contrary, and the nerves arising from it, are the organs of touch.

Baron LARREY has likewise given some share of his attention to the nerves, although in a very different way from the professed

* Note in the *Arch. Gen. de Medecine*, tom. iii.

† While this sheet was passing the press, we had an opportunity to become acquainted with further results of M. MAGENDIE's researches, and to witness many of his experiments: of these, an account will be found in another part of this Number.

‡ *Annali Universali di Medicina*, 1824.

experimentalists. The observations of this veteran surgeon have been principally made on the human body, during surgical operations undertaken in consequence of wounds received in battle; and any facts thus indirectly ascertained are, at least, as valuable as those brought together by the express institution of experiments. He has frequently observed* that the division of the nerves of the brain causes acute, but momentary, pain; that the cut extremities, instead of retracting, seem rather to become a little elongated, and to touch each other; whilst all the other soft parts, when recently divided, retract and separate from each other with more or less force; that the cut ends of nerves swell, or bulge out, to a certain distance, and form at their summit a rounded unequal eminence, from whence arise very slender filaments, (doubtless formed by the neurilema,) which unite with the surrounding parts, and lose themselves in the cicatrices, which become very sensible. When these nervous extremities are exposed to the contact of the atmosphere, they inflame, and become covered with fleshy granulations formed by the vessels of the neurilema; and the parts thus inflamed are extremely sensible and painful, so that they cannot be touched without exciting convulsions. When the two extremities of a divided nerve are reunited by spontaneous cicatrization, the nerve, as is well known, again transmits the stimulus necessary to restore the functions of the part to which it is distributed. According to M. Larrey, this communication is not effected by means of any anastomosis, but each nervous filament which enters into the formation of a nerve fulfils a certain specific function, which cannot be supplied by any other; thus resembling the metallic wires which form the common cord of the electrical telegraph of SŒMMERING.

These statements, however, are not peculiar to M. Larrey; but a circumstance follows which we believe is not generally known,—it is that two distinct nerves, which have been divided during an amputation, may unite together in the stump end to end. He amputated the right arm of a soldier, named Glass, in 1821, for scrofulous ulcers and caries of the bones of the fore-arm. The patient died in the summer of 1823, of tubercular phthisis; and, on examining the stump, it was found that the cicatrix, which had become linear, was depressed in the centre, and appeared to have formed vascular connexions with all the subjacent parts, including the periosteum of the bone. This external envelope having been removed by careful dissection, it was found that the cut end of the bone was become thin and rounded, the medullary cavity being almost obliterated; the humeral artery and vein were united at their extremity, and their cavity destroyed to the extent of some lines. The trunks of the median and external cutaneous nerves were united end to end by their divided extremities; and an incision made through the cicatrix afforded no trace of intervening cellular texture, the substance of the two nerves appearing to be blended together, like natural anastomoses formed by the sides of certain nervous trunks which have separate destinations. The two trunks of the brachial plexus, which

---

* *Notice sur quelques Phenomenes observés dans la Lesion des Nerfs, et dans leur Cicatrization.* Par M. le BARON LARREY.—(Revue Medicale, Mars.)

runs to the posterior part of the arm and fore-arm, were united in a similar manner.

A case of *inflammation of the spinal marrow, connected with disease of the kidneys,* related by Dr. JONES, possesses some interest from its relation to the recent experiments on the functions of the anterior and posterior roots of the spinal nerves. The power of moving the limbs was but little affected, but there was considerable numbness and loss of sensation, affecting the back especially. "Now the examination of the spinal cord shewed that, though the sheath, and the cellular substance between it and the inside of the vertebræ, was most inflamed at its anterior part, the enlarged blood-vessels exercised most pressure at the posterior and lateral parts."* It is almost unnecessary to remark, that this account, so far as it goes, is corroborative of the doctrines of Mr. CHARLES BELL and MAGENDIE.

In our last Proëmium, we mentioned the observations made by M. DESMOULINS on the peculiarities of the brain in fishes: we have now to lay before our readers some further researches, by the same physiologist, on *the organs of vision* in different classes of animals.† Careful dissection has given M. Desmoulins occasion to remark a folding of the optic nerve, and of the retina itself, in eight different species of fish, and that those possessed of this arrangement are gifted with more energetic vision than the others. From this disposition of the retina it follows that, when unfolded, it occupies a much larger extent than the membrane which envelops it. Further researches, made on those birds whose sight is most piercing, confirm the results already mentioned, and convert them into a general law. In some of the eagle and vulture species, the retina is folded on itself in such a way as to resemble the meridian lines of a sphere; the folds being deeper and more distinct in the eagle than in the vulture. In these birds, and in some others, the optic nerve is surrounded by a fibrous sheath, which does not adhere to it, but is continuous with the sclerotic coat and the dura mater. In the curlew there is a slight puckering, by which an increase of surface is gained, equal to the tenth of the whole sphere. In the diver *(colymbus minor)* the optic nerves are not longer than the fourth-part of the diameter of the eye; the retina is smooth throughout the posterior fourth of the sphere, and very much puckered throughout the rest of it; so that, if unfolded, it would occupy double the extent of the spherical segment where they are situated. In the mallard, the turkey, and some others, the retina is smooth as in man. It does not appear, then, that the folding of the optic nerve necessarily coincides with that of the retina.

After demonstrating that there is a constant coincidence between the

---

* *Case of Inflammation of the Spinal Cord, combined with Inflammation and Suppuration of the Right Kidney; with the Appearances after Death.* By WILLIAM JONES, M.D. &c.—(Edinburgh Med. and Surg. Journal, April 1824.)

† *Memoir sur le Rapport qu'a l'etendue des Surfaces de la Retine et du Nerf Optique des Viseaux avec l'Energie et la Portée de leur Vue.* Par M. A. DESMOULINS, D.M.P.—(Journal de Physiologie Exper.)

energy of vision and the proportional extent of surface in the retina and optic nerve, the author shows that this anatomical disposition has a constant relation to the size of the tubercula quadrigemina, in birds and fishes. M. FLOURENS considers this portion of the brain as merely the conductor of vision, the cerebral lobe being the boundary of sensation and the site of its perception. On the other hand, according to M. Desmoulins, in those animals in which the cerebral lobes are either wanting, or of which a rudiment only exists, the brain is not the seat of the sensation; and thus, in fishes, he regards the optic lobe, or tubercula quadrigemina, as the seat of vision. He likewise impugns the accuracy of Flourens on another point,—viz. in holding the cerebellum to be the balancer and regulator of determinate movements, as this function exists in animals destitute of the organ in question *(les batraciens)*. CUVIER says that, in these animals, it is only a small triangular body. Now, M. Flourens talks in his Memoir of having removed *both lobes;* which is regarded as a proof that he mistook the part.

Some important observations have likewise been made by M. Desmoulins with regard to *the coloured and reflecting surfaces of the choroid, and the corresponding degrees of perfection in vision.* In cats and some other mammalia, in the sturgeon and various fishes, the whole concavity of the choroid is uniformly coloured of a pearly white, with metallic reflections, which in some species are not inferior in lustre to polished silver. In some *(squales),* a zone of the choroid, touching the iris, is black; but its situation is such as to prevent it from receiving any image or ray of light. Thus it appears that, in various mammiferous animals, and in certain fishes, there is not the smallest black spot on the posterior part of the eye; and that in the sturgeon, and some others, the inner surface of the iris reflects the rays of light. On the other hand, in the hedgehog and the owl, both nocturnal animals, the concavity of the choroid is either black or brown. Those animals which have the choroid coloured generally have the pupil placed longitudinally, of which no example is to be found where the choroid is black or brown: neither does M. Desmoulins suppose that any animal, really a nyctalope, has the choroid black. In short, the idea of this physiologist is, that the coloured choroid acts by reflecting the rays in such a manner as to produce a double image; thus resembling the effect of the folds existing in birds, although the multiplication takes place to a much less extent, and depends upon a mechanism entirely different. In the sturgeon, the two contrivances (of a reflecting and folded choroid) are united at the inferior extremity of the eye.

In our Number for May, we gave an extended account of Dr. KELLIE's paper on *the circulation in the brain,* and we have now to notice a very interesting communication on the same subject, by Dr. CARSON, of Liverpool.* This gentleman adopts the opinion (concerning the accuracy of which, we conceive, little doubt can be entertained,) that, as the limits of the cranium are fixed, the quantity of its

---

* *On the Circulation of the Blood in the Head.* By JAMES CARSON, M.D. Liverpool.—(Edinburgh Med. and Surg. Journal, April 1824.)

actual contents must be always the same, although many variations may occur with respect to the relative proportion of fluid and solid parts. It is obvious, however, that, as the substance of the brain cannot during life undergo any very sudden change, so these variations must principally regard the fluid contents,—that is, the relative quantities of blood in the vessels, and of water in the ventricles. Neither, however, can this last be regarded as subject to much change in the healthy state of the body; and, if we thus suppose two of the three constituent parts of the contents of the cranium remain the same, by what means is the alternate entrance of the third into the brain, and its removal therefrom, to be accomplished? To the solution of this difficulty, Dr. Carson applies the resiliency of the lungs, a principle of which he has formerly made such ingenious use. He supposes a portion of the atmospheric pressure to be removed from the blood, at the ends of the sinuses communicating with the veins outside the neck, by the dilatation of the heart and the resiliency of the lungs having a constant tendency to increase the capacity of the veins within the thorax. But this abstracting power, although generally aided by gravity, is insufficient to suck out the blood from the head, except at the moment when the arteries, by their contraction, are ready to introduce a quantity of blood into the head, equal to that which the veins are endeavouring to abstract, and thus the blood is equally circulated through the whole head. It has been common to attribute the phenomenon of the pulsation so frequently observed in the jugular veins, to the impulse communicated to them by the contiguous arteries; but, according to the view just given, a very different explanation presents itself: for, as it is contended that the arteries convey the blood to the head in synchronous jets, and that at the same instant, and then only, the veins are enabled to abstract it, so it is obvious that a succession of currents must pass through the veins, whose alternate dilatation and collapse give rise to the phenomena in question.

These views, as well as those of Dr. Kellie above alluded to, suppose the quantity of blood within the cranium to continue always the same, while the other contents remain unaltered. We now come to a different proposition. It is generally admitted that, in particular diseases, the substance of the brain is more or less wasted; and this must give rise to an increase of one or both of the other contents of the encephalon,—probably, for the most part, to the latter. Supposing the blood to be increased in quantity, it is obvious that there must be a limit to this accumulation, as the vessels cannot be supposed capable of unlimited distention. Now, the object of Dr. Carson is to show that the use of the ventricles is to guard against this danger, by affording receptacles for water, by which the space that would otherwise be a void is conveniently filled up, without rendering it necessary for the vessels to become too much enlarged.

"On the dissection of bodies reduced to great emaciation by disease," says Dr. Carson, "the vessels of the head are found to be turgid, the substance of the brain to be soft and to contain an unusual quantity of blood, and the ventricles to be greatly distended with water. Sometimes, in such cases, the quantity of water contained in the ventricles is very considerable; ten ounces is by no means uncommon. Suppose there had existed no receptacles for water, such as the ventricles, and the brain to have been wasted to that degree by which room was afforded for the admission of ten ounces of water into the encephalon, the blood-vessels of the head must necessarily have been loaded with ten ounces of blood, in addition to the quantity which they already possessed, and by which they appear to have been already too much distended. Long before they could have been distended to the capacity necessary for the admission of so great a quantity of blood, their coats must have given way, and a fatal hemorrhage ensued; or, at all events, they must have been too much surcharged for the performance of their functions. Hence, we readily perceive the important uses of the ventricles. By becoming the receptacles of a mild fluid, they, in certain circumstances, prevent the blood-vessels from being over-distended. By their greater or less expansion, they become the grand regulators of the circulation of the blood through the head. Water in the ventricles, in such circumstances, instead of being considered a disease, is in reality the great remedy provided by nature for the preservation of life, in situations in which it could not otherwise exist. It is the defence set up by nature for the protection of the breaches or weak points which may exist in this part of her works.

"The ventricles of the brain, in consequence of their irregular course, are admirably situate for enabling the substance of the brain to assume that variety of position necessary, as circumstances alter, to give due support to the vessels of the head, without sustaining at any point a disproportionate distention. But, to perceive this sufficiently, the brain itself must be examined."

Those views of the subject appear to us highly interesting and satisfactory. We think it is extremely probable that, when any portion of the brain is removed, it may give rise to synchronous and corresponding distention of the blood-vessels; and that these, being thus distended to a certain extent, and for a certain time, may relieve themselves by serous effusion into the ventricles. So, on the contrary, if the quantity of the solid substance of the brain be increased, little water will be found in the ventricles; the space assigned to the blood-vessels is encroached upon, it may be, to such an extent as not to leave sufficient room for its circulation: hence, though unable to gain admission, the blood is driven to the external parts of the head, and gives rise to that redness of the face and appearance of fullness about the head which characterizes those predisposed to apoplectic seizure.

Our countryman, Dr. EDWARDS, who has become naturalized in France, and whose name we have frequently had occasion to mention with respect, has lately published an extensive work upon *the influence of physical agents upon animal bodies*, by which appellation he designates the air, water, temperature, light, and electricity.* Those causes, in some of their modifications, exert a silent but constant agency, and the method adopted to measure the effects thus produced was that of

---

* *De l'Influence des Agens Physiques sur la Vie.* Par W. F. EDWARDS, D.M. &c. &c.—Paris, 1824.

experiment upon each of the four classes of vertebrated animals.—Among these physical agents, the atmosphere is most universal in its operation, and most important in its effects; yet there appears reason to believe that, much as the phenomena connected with it have been studied, from the time of GOODWIN to the present day, too great importance has been attached to the relation subsisting between the atmosphere and the lungs, or, rather, this alone has been studied, while the effects resulting from the influence of the medium in which an animal is placed upon the surface of the body, has until lately been nearly overlooked. In some classes of animals, indeed, the influence of this agency is acknowledged; but its existence does not seem to be confined to those modifications of life which prevail in the species which constitute the lower ranks of creation,—at least, if we admit the theory of M. GEOFFROY respecting the respiration of the fœtus. The influence of the medium, independently of respiration, was ascertained by Dr. Edwards, by an ingenious application of the wonderful property possessed by reptiles of retaining the free exercise of sense and voluntary motion for a certain period after the excision of the heart. By this operation, the circulation is arrested, and the function of respiration ceases, as a necessary consequence. The result of this is to leave only the nervous and muscular systems in operation. If, when this has been done, some animals be placed in air, and others in water, the difference between the period during which life is prolonged under these two circumstances will indicate the respective influence of the media upon the nervous and muscular systems, *independently of respiration and circulation.*

Such was the plan adopted by Dr. Edwards. He cut out the hearts of four salamanders, two of which were exposed to the air, and two placed in water, which had been freed from air by boiling: for a short time they were all equally lively, but their activity gradually diminished, and was only manifested at long intervals. At the end of four or five hours, those in the water appeared to be dead, but they still moved on being pinched: one died at the end of eight, and the other of nine hours. Those which had been left in the air, on the contrary, lived twenty-four and twenty-six hours. These experiments were frequently repeated, as well on salamanders as on frogs, and with similar results, except that, in these latter animals, the period during which they survived in the air was not so much greater.

The difference in the effects of the media was further illustrated, by placing a frog, under the circumstances above mentioned, in water, and, when it no longer gave any sign of life, removing it into the air, by which it recovered and began to move. This experiment was likewise reversed.

Another modification of these experiments consisted in preventing respiration by tying pieces of bladder over the heads of frogs, so as to produce strangulation. They were paralyzed at first, but gradually recovered their strength to a certain extent, and lived from one to five days: while those immersed in water, under similar circumstances, died at the end of ten or twelve hours. It was further ascertained, by inclosing frogs, with the head thus enveloped, in vases of atmospheric air, that a portion of carbonic acid was formed, thus properly constituting cutaneous respiration. This had been previously ascertained by SPALANZANI, but in a less satisfactory manner, as there were sources of error in his experiments, which it is unnecessary here to detail.

A very curious question has received conclusive elucidation from the investigations of Dr. Edwards: we allude to the singular fact of certain animals, particularly toads, remaining alive for indefinite periods, although enclosed in solid bodies. Most of our readers are probably aware of the famous experiment of HERISSANT, who enclosed three toads in boxes sealed with plaster; two of which were found alive at the end of eighteen months. The account of this experiment is not very satisfactory in its details, as no mention is made either of the size or materials of the boxes employed; and there is reason to believe that a certain portion of air was present in them. Dr. Edwards, in order to guard against these objections, took boxes about four inches square, and, having put some plaster in the bottom, he placed the toads in them, and, surrounding them on all sides with plaster, shut and secured the boxes. The circumstance to be ascertained was—whether these reptiles, deprived of air by the contact of a solid body, or by immersion in water, would survive longest; and it is sufficient at present to remark, that they lived much longer in the plaster than in water. A fact sufficiently remarkable, but what appears more extraordinary still, is that they lived longer when enclosed in a solid body than in air. Four frogs were confined in a dry jug, and an equal number were placed in dry sand: the third day, all those confined in air were dead, except one, while all those enclosed in sand were alive, except one; from which it would appear, not merely that these reptiles can live when surrounded by solid bodies, but that placing them in this situation is a means of prolonging their existence; a conclusion which is in accordance with those well-authenticated narratives of animals of this class having been found in the centre of solid masses, where they must have been enclosed during periods concerning the duration of which it would be in vain for us to indulge in conjecture.

That the sand employed in the last-mentioned experiment contained air is obvious, and that the plaster was pervious to air was proved by the following experiment:—An open tube was corked with wet plaster to the extent of an inch; after it was dried, more plaster was applied, to cover any imperceptible apertures; the tube was then filled with mercury, and inverted over a vessel of the same: the air entered through the plaster, and made the quicksilver sink in the tube. But as it might be said that, although some air passed through the plaster, yet enough to sustain life could not be supposed to find its way through so dense a body, toads and salamanders were enclosed as before, and the boxes buried in water and quicksilver: they now died as soon as when merely immersed without any covering. It would thus appear that the fact of these reptiles living in solid bodies is not an exception to the general law, which regards air as necessary to the support of animal life. The fact of their surviving longer in plaster or sand than in air, seems to depend upon the waste by evaporation being thus lessened; it having been found, by statical experiments, that, *ceteris paribus*, a frog confined in air became emaciated and shrivelled with much greater rapidity than when surrounded by solid materials; the rationale of which is too obvious to require explanation.

The influence of cutaneous respiration was further illustrated by preventing the action of the lungs. As the mouth of these animals is necessarily shut during respiration, to enable them to throw air into the lungs by a movement of deglutition, Dr. Edwards took advantage of this circumstance, by placing a piece of stick between the jaws, so as to prevent them from being closed, and retaining it in this situation by a particular apparatus. This contrivance impeded, but does not appear to have entirely arrested, the action of the lungs, and therefore the results can scarcely be regarded as satisfactory; nor the method, one proper to be adopted in repeating these experiments. A ligature was applied so as entirely to exclude the access of the air; the reptiles were placed on wet sand, and lived for a considerable time,—one of them for twenty days after complete strangulation, although, when similarly treated in water, they died in from one to three days; a result which is regarded as proving the beneficial influence of the air upon the skin. Yet another and a very important modification of this experiment consisted in the entire extirpation of the lungs, the peculiar construction of which in these animals renders it capable of being done without making an extensive wound: an incision of two or three lines in the flank permits of their extraction, while a ligature placed at their root prevents any effusion of blood. This operation was performed on three frogs, the external wounds being closed by sutures: they seemed to suffer little, and were soon as lively as before; two of them lived thirty-three days, and the other forty.

We have thought it proper to give these experiments a place in this compilation, not on account of their absolute novelty, but because they appear to us more precise than those of Spallanzani, or any previous experimentalist on this subject; and to place the importance of the skin as an organ of respiration in a very clear light, so far as regards the animals experimented upon. The work contains many interesting doctrines, particularly concerning the action of temperature and different media upon animals: almost all of these, however, were published in detached Essays, which have appeared in the French periodicals at various times during the last five or six years, and consequently the most important of them have already been noticed in this Journal.

M. Defermon has performed some experiments on different animals, with the intention of examining under what circumstances contraction of the spleen takes place, and what causes effect variations in its volume. It appears that certain substances, as strychnia, camphor, acetate of morphia, and others, which affect the nervous system, produce contraction of the spleen. When strychnia, for example, is given to a dog, this organ, as soon as the poison is absorbed, becomes rolled up in a spiral form, and exhibits powerful contractions, *(se roule en spiral et presente des contractions fort energiques.)* If camphor be given, a different species of contraction takes place; the spleen, which is generally smooth, becoming rugose, presenting eminences which augment and diminish in volume, producing a degree of movement throughout the whole viscus. It is added, that, when the relation between the number of pulsations and respirations in the minute changes, the state of the spleen undergoes a corresponding alteration; that is, it increases or diminishes in size. Suspension of respiration likewise affects the degree of distention of the spleen. M. Defermon is engaged in the prosecution of these inquiries with a view to publication.*

One of the best of the few cases on record in which *the skin of the Negro has become white*, is related by Mr. BROWN, in the Transactions of the Medical and Chirurgical Society of Edinburgh. The change went on gradually, but progressively, and at the end of eighteen months (the period to which the report extends,) the extremities and head were of a natural white appearance; the breast, abdomen, and back speckled; and the change making regular progress.

The most important work connected with physiology which has for some time appeared in this country, is the "Elementary System" of Dr. BOSTOCK.† Independent of its intrinsic merits, it is further remarkable as the first regular Treatise on Physiology which has issued from the English press; a circumstance not a little extraordinary, when we reflect upon the active share which our countrymen have had in promoting the advancement of all the sciences connected with medicine. To supply this want has been the object of Dr. Bostock; and, in the work to which we allude, the student will find a concise but interesting account of the various theories and hypotheses which have at different times prevailed in the medical world, as well as an instructive view of the present state of our physiological knowledge. As, however, a work of this kind necessarily presents little which has not already appeared under different forms, we shall, on the present occasion, content ourselves with having thus briefly alluded to it.

---

### MORBID ANATOMY AND PATHOLOGY.

As the anatomy of healthy structures seems naturally to precede Physiology, so Morbid Anatomy may with propriety be placed immediately before General Pathology.

A very curious example of what Dr. DUNCAN has denominated *bifid brain*, has been recorded by that gentleman in the Edinburgh Medico-Chirurgical Transactions;‡ and a circumstance which gives additional value to the description of this malformation is, that it comes from the pen of the late lamented Dr. GORDON. We have many inducements for transcribing the narrative into our pages, which, independent of its relating to a very rare species of deformity, may be regarded as a model

---

* *Bulletin des Sciences Medicales*, Fevrier.

† *An Elementary System of Physiology.* By JOHN BOSTOCK, M.D. F.R.S. L.S. & H.S. M.R.S. Vol. I.—London, 1824.

‡ *Case of Hydrocephalus, with Bifid Brain.* By ANDREW DUNCAN, jun. M.D. &c. &c. *With a Description of the Malformation*, by the late JOHN GORDON, M.D. F.R.S.E. &c.—(Transactions of the Medico-Chirurg. Society of Edinburgh.)

for such reports ; and to those of our readers who had the good fortune to be acquainted with Dr. Gordon, the case will be received with interest, as the posthumous production of one whose penetrating mind appeared well qualified to give a fresh impulse to the sciences he cultivated.

The child, which forms the subject of this paper, was a female, born hydrocephalic, and which lived seven months. All the external senses and functions appeared to be natural ; she was occasionally lively, and evidently received pleasure when any one played with her ; but could never sit up, on account of the weight of her head. The day after her death, the body was examined by Dr. Gordon, in the presence of Dr. Duncan and various other gentlemen, and the following description of the appearances drawn up by him :

" *Notes of the Anatomy of the Head.*

" I. *Of the Parietes of the Cranium.*

" 1. *Shape and Dimensions externally.*—I measured the head, I think, in May or June. It was 29 inches round by the frontal and occipital protuberances, and 16½ over the vertex from one ear to the other ear. I did not then take any outline of its shape.

" At the dissection, the form of the head was somewhat like this—

" The dimensions were 28½ inches in its greatest circumference, and less tense than before death.

" When the back part of the head, behind *a b*, was placed between the eye and the light, it was distinctly translucent.

" 2. *Parts composing the Parietes.*—A. *The Integuments.*—Had a thin appearance, and were actually thinner than natural. Large ramifications of veins were very visible in the regions of the temples and occiput. There was no appearance of granular adipose substance under the cutis vera, such as is always to be seen in children of the same age in a state of health.

" B. *The Bones.*—Both halves of the frontal bone were fully a fifth larger than they are naturally at this age, and they were separated from each other, at the anterior superior fontanelle, fully an inch and a half; but they gradually approached each other below, so as at last to come in contact, in the usual manner, in the origin of the nasal process. Their structure and thickness were not different from the natural. Both parietal bones were between a fourth and a fifth larger than they are in a full-grown subject, and were as thick as in a child of six months, or rather more. Their margins were separated from each other, and from the frontal bone, about an inch and a half. The occipital part of the occipital bone, like the frontal, was about a fifth larger than it commonly is at this age, but was of the usual thickness and structure, and separated from the parietal bones by a space not less than three, but nearer four, inches. The squamous portions of the temporal bones, and the large wings of the sphenoid, were also larger than natural; and between their margins there were considerable intervals, and they were more indistinct outwards than natural. But the parts of the basis of the cranium, which occupy the median plane and its vicinity, were but little affected.

"3. *The Dura Mater.*—The whole inner surface of the cranium was lined, as usual, with dura mater. It adhered closely to the bones where they existed, and, where they were deficient, it was united to the inner surface of the integuments. It had the same thickness and structure as in health. Its falciform process was of the usual depth or breadth, but greatly longer. It stretched from the region of the frontal spine to the internal transverse ridge of the occipital bone, as usual, and there terminated in the tentorium cerebelli. The tentorium itself was very little enlarged or altered in its situation or appearance.

"II. *Of the Contents of the Cranium.*

"1. *The Water.*—This was drawn off by a puncture through the most prominent point of the occiput; a blowpipe being immediately introduced to facilitate its discharge. By this opening all the water was evacuated. The quantity was 136 ounces by weight. It had the transparency and want of colour which are so remarkable in this secretion, being exactly like the purest spring-water.

"2. *The Brain and its Membranes.*—As soon as the whole water had been evacuated, an incision was made with a pair of scissors, first transversely on the left side, from the puncture between the occipital and parietal bones, for about two inches, and then, at right angles to this, a little to the left of the median plane, from the puncture all the way forward to the brow.

"When the opening in the course of this incision had been made sufficiently large, we looked through it into the interior of the cranium, conceiving it possible that appearances might be advantageously seen in that stage of the dissection, which might afterwards become less distinct; and certainly the aspect of parts was very striking. The brain occupied the lower part of the cavity alone; the rest was entirely empty, excepting only that the falx projected down into it, but still separated from the brain (between the hemispheres of which it naturally penetrates) nearly two inches.

"The cavity being fully exposed, and the parts minutely examined, the following was the result:—Those surfaces of the two hemispheres of the *great brain,* or brain proper, which are usually applied to the falx of the dura mater, were separated from each other for about four inches, except within an inch of their anterior extremities, where they remained united in the natural manner. The corpus callosum was wholly wanting, except two white bands, which stretched across between the anterior horns of the ventricles, nearly parallel to each other, about a quarter of an inch apart, and each of them from an eighth to a quarter of an inch broad. We took these to be vestiges of this body. I endeavoured to produce a fibrous laceration in these bands, but their softness did not admit of it. The fornix had also almost entirely disappeared:—perhaps we ought to say, entirely; for there remained only a round white cord, of the diameter of a crow-quill, on the right side, which ran from the region of the anterior pillars of the fornix, closely tied, by means of the pia mater, to the lower surface of the convolution which usually overhangs the corpus callosum, backwards towards the commencement of the hippocampus, in the inferior horn of the right lateral ventricle, where it gradually disappeared. Yet this cord had been little analogous in its structure to nervous matter; it was firm and tough, and not easily torn. The septum lucidum was entirely gone. There was not the slightest appearance of the anterior commissure of the brain remaining. The folded layers of white nervous matter anterior to the pineal gland, commonly called the *posterior commissure,* were quite distinct, and both broader and thicker than usual. The ventricles were considerably enlarged; and, from the disappearance of the corpus callosum and fornix, they formed one common cavity with the parietes of the cranium, and a membrane afterwards to be described. Had they been quite shut up, they would perhaps have contained about * * * * of the whole fluid found within the head. When the brain was first examined, it presented an appearance like that which anatomists daily produce, when they divide the corpus callosum and fornix longitudinally from behind forwards, and allow the hemispheres to separate from each other by their own weight. But, owing to the absence of the parts mentioned, the surface exposed was much more extensive in this case. Besides, the depth or breadth of that flat surface of each hemisphere, which in a healthy cerebrum is applied to the falx, and is bounded by the corpus callosum below, was in this instance a good deal diminished. This change had taken place to a considerably greater extent on the left than on the right side.

"Hardly any part of those convolutions and white nervous matter on which they rest, which form the median surface of the left posterior lobe, remained. The parts looked as if the inner wall of the posterior horn of the left lateral ventricle had been shaved away. In consequence of this, the surface of the inferior horn was freely exposed, and a full view might at once be obtained of the whole cornua of this ventricle. On the right side, although one could, from the absence of the posterior extremity of the corpus callosum and fornix, see almost down to the anterior extremity of the inferior horn, without displacing the parts, yet the inner surface of the posterior lobe was not more narrowed than the

* Not filled up by Dr. Gordon.

portions of the hemisphere more anterior to it. The third ventricle was considerably widened. The flat inner or median surfaces of the optic thalami were at least half an inch asunder. The cavity of the infundibulum was enlarged in proportion; but the aqueduct of Silvius, or passage to the fourth ventricle, was of its usual dimensions. The tela choroidea and plexus choroides had entirely disappeared, so that the third ventricle communicated, at all points, freely with the lateral ventricles and the general cavity of the cranium. The only change in the corpora striata and optic thalami, was their being less elevated than usual. The tænia semicircularis was very distinct. The colliculus, or ergot, in the posterior cornu of the left hemisphere was entirely gone, the very wall from which it projects having disappeared; nor was this eminence at all perceptible in the right hemisphere. Both hippocampi remained in the inferior cornua, but they were broader and less elevated than usual. The band on each of them, called the *tænia hippocampi*, so remarkable in a healthy brain, was entirely wanting; and, in consequence of this, the inner border of each hippocampus flowed gradually into the inner convolution of each middle lobe. The whole inner surface of the ventricles was quite smooth, and lined with an epithelium, preserving all the increased thickness and strength which is so remarkable in every case of hydrocephalus.

"The thickness of white nervous matter within the cavities of the ventricles and the basis of the convolutions, was diminished proportionally to the enlargement of these cavities. Opposite to the junction of the posterior and inferior cornu of the right lateral ventricle, it measured only a sixth of an inch.

"The only part where the convolutions were distinctly diminished in depth, was opposite to the posterior horn of the left lateral ventricle. Here they were fully one-half lower or shallower than usual. At all other parts they were of their usual dimensions, and, on being divided, exhibited the usual proportion of white and brown matter.

"All the parts in the region of the basis of the brain were entire. The olfactory and optic nerves, and the motores oculorum, could be distinctly traced to their origin; and they presented no appearance of derangement of structure.

"The only change which the substance of the cerebellum had undergone, was a little flattening of the left hemisphere, and proportional elevation of the right; and the vermiform processes and parts usually occupying the median plane, were placed about an eighth of an inch to the right of this plane. Internally, its structure was perfectly natural; and the fourth ventricle had undergone no enlargement. All the nerves issuing from it, viz. the trigeminal, the pathetic, the abductores oculorum, the facial, and the auditory pairs, were quite entire.

"The medulla oblongata, and the nerves springing from it, were in a healthy state.

"The pia mater presented nothing unusual. A whitish tough cord, of the diameter of a crow-quill, ran across from one hemisphere of the brain proper to another, which, on minute examination, was found to be a tube, and seemed obviously to be a branch of the arteria corporis callosi.

"All the parts which were provided with pia mater, were likewise covered with a natural arachnoid membrane.

"One of the most remarkable effects, however, resulting from the disease in this instance, was the production of a new membrane, which was interposed between the dura mater and the water, over the whole extent of that part of the enlarged cavity of the cranium which was not occupied by the brain. This membrane was perfectly transparent and colourless, without any appearance of laminæ or fibres, or vessels of any kind. It was soft and flexible, and about three or four times thicker than the arachnoid membrane, even where that membrane is thickest, in the region of the basis of the brain. In short, it resembled, in a remarkable degree, the amnion of the gravid uterus. It arose below from the whole inner and upper edge of each hemisphere, from the external border of the posterior lobe, and from the inner convolution of the middle lobe, along that line to which the arachnoid membrane is affixed in the basis of the brain; but it was not continuous with that membrane at this part. Extending upwards from these attachments, it lined closely the whole inner surface of the dura mater, and terminated on each side by being attached, along its whole upper and median border, to the root of the falciform process, along the course of the longitudinal lines. The following rude diagram, representing an imaginary section across from ear to ear of the dura mater, and the parts within it, will perhaps illustrate the connexions of this membrane:

The outer strong line, *a b c*, is a section of the dura mater; *b d*, a section of the falciform process; and *e*, a section of the superior longitudinal sinus in that process. The dotted line, *f g h*, represents the outline of a section of each hemisphere of the brain proper. The fine line, *i k*, represents a section of the new membrane, issuing from the upper edge of each hemisphere below, and attached to each side of the root of the falx at *k* above.

"At its origin from the hemispheres, its root could be easily separated into two layers for the extent of about an eighth of an inch, and each layer was continuous with the arachnoid membrane, crossing the convolutions thus:— Its outer surface was applied close to the dura mater, but it was not attached to it at a single point, except close to the falx above; so that it was quite movable upon it, and could be raised from it with the utmost ease. Towards the fore part, three large venous trunks could be seen running upon its inner surface towards the longitudinal sinus, into which they opened. These corresponded exactly to the large anterior venous vessels of the pia mater in a healthy brain.

"The whole great brain, or brain proper, weighed, with the pia mater and arachnoid membrane, 16¾ ounces.

"The right hemisphere weighed 8½ ounces, the left 8¼, or ¼ ounce less than the right.

"The weight of the whole cerebellum, with its membranes, and the medulla oblongata attached to it, was 2½ ounces; so that the weight of the whole brain was 19¼ ounces."

Dr. Duncan is of opinion, that the membrane described by Dr. Gordon is not to be regarded as a new one, but as the arachnoid coat, morbidly distended and thickened. In this supposition we cannot agree, as it is expressly stated that there was "a natural arachnoid membrane" besides.

In a recent Number of the Revue Medicale, M. Bayle has related some cases of disease in different parts of the *brain* and *spinal marrow*, which appear to us more valuable than all his numerous experiments and physiological speculations. The first case, when viewed in conjunction with others of a similar nature, may be regarded as proving that the general doctrine, which constitutes the brain the exclusive seat of sensation, is not true,—at least in its full extent. It likewise presents what we believe has at present no parallel in the records of pathology,—viz. an example of a cancerous, or rather of an encephaloid, tumor in the vertebral canal. A washer-woman, aged fifty-two years, suffered at first from severe lancinating pains in the thorax and abdomen, and afterwards in the pelvis and lower extremities; after a time, these became affected with convulsions, and at length with perfect flexion, immobility, and insensibility,—except that they suffered occasionally from shooting pains, which originated in the pelvis, and appeared to follow the course of the nerves. On examination after death, the spinal marrow was found to be healthy as far as the tenth dorsal vertebra, where, on its posterior surface, a tumor was found between the folds of the arachnoid. This tumor was of an oblong shape, about two inches in length, and placed longitudinally in the medullary canal; its internal structure resembled the substance of the brain, but was firmer; it had no adhesion to the spinal marrow, which for two inches was rendered very soft throughout its whole thickness, and which, at the most prominent point of the tumor, appeared divided transversely; so that the two portions, separated by a slight interval, resembled two cones placed with their summits towards each other. An attentive examination of the parts did not lead to the discovery of a single fibre which remained free from the disorganization. The limbs were wasted, but the nerves did not appear to be smaller than usual. The most important part of this relation is, that, while the communication between the upper and lower portions of the spinal cord seem to have been cut off, the inferior extremities were nevertheless the seat of frequent lancinating pains.

Four examples (and, so far as we know, only four,) of a similar nature have been recorded: one by DESAULT,* which took place in a man who had been shot in the back, at the right side, and before the inferior angle of the scapula. This patient lived above twenty-four hours, voiding his urine frequently and without difficulty, and being able to move the pelvis and inferior extremities up to the moment of his death. The ball had entered the chest between the eighth and ninth ribs, went through a portion of the right lung, penetrated the right side of the tenth dorsal vertebra, and entirely divided the spinal marrow. This is probably the only example in which the sudden division of the spinal cord has not been followed by paralysis of the parts beneath it; in all the other instances, the interruption has been the result of changes effected slowly. This case, it is true, falls not within the period assigned to the present compilation; but we have alluded to it, as interesting in relation to the others.

A second example of division, or rather interruption, of the spinal marrow, will be found in our Number for July, 1823, related in MAGENDIE'S Journal, by Dr. RULLIER: in this case, the patient retained the free use of his lower extremities, notwithstanding the almost complete destruction of a great portion of the spinal cord. And in the work lately published by M. OLLIVIER on the Spine,† we find two more instances of a similar nature: these we shall here record.

A child, of eight or nine years old, of scrofulous constitution, fell a victim to caries of the vertebræ, accompanied by intense and continual head-ache, but retaining to the last the feeling and power of motion in the lower extremities, although they were weak and wasted. On opening the body, a complete interruption of the spinal cord was discovered, extending from the ninth dorsal to the first lumbar vertebra, —that is, about four inches. The envelope of the marrow was flattened, but presented no other alteration. The upper part of the cord terminated at the interruption in a sort of bulb; there was no medullary matter in the intervening space, but the sides of the sheath were in contact without adhering.

The last example is as follows:—A young girl, thirteen years of age, died in consequence of a disease of the spine, accompanied with protuberance of the vertebræ in the dorsal region. The patient had been able to move the thighs and legs, and had got out of bed three or four days before her death, the viscera of the pelvis, &c. having suffered no interruption in their functions. On examination post-mortem, two vertebræ were found carious, with a considerable flattening of the marrow throughout the extent of five inches; the membranes were inflamed; and, "at the lower part of the dorsal region, the nervous pulp, reduced to a putrid state, was converted into a pultaceous matter, and deficient for four or five lines."‡

---

* *Journal de Chirurgie de* DESAULT, tom. iv.

† *De la Moelle Epiniere, et de ses Maladies, &c. &c.* Par C. P. OLLIVIER D'ANGERS, &c.—Paris, 1824.

‡ *Lib. cit.*

In allusion to cases of this kind, M. Bayle asks in what manner the phenomenon is to be explained, and suggests the four following hypotheses:—1. Either the spinal marrow can serve as a conductor of sensation and motion, notwithstanding the entire disorganization in some part of its length; 2, or the enveloping membranes can discharge this function; 3, or the external impressions and the determinations of volition may be propagated by the anastomoses of nerves; or, lastly, the limbs can derive sensation and the power of motion from a portion of the spinal column which has ceased to communicate with the brain. He gives the preference to the last of these conjectures.

Another case related by M. Bayle is of considerable interest, from its relation to some of the many hypotheses prevalent in France about the functions of the cerebellum. A woman, aged seventy-two, enjoyed very good health up to the 14th of January, 1824, when she was suddenly seized with giddiness, and fell to the ground in an apoplectic fit. No palsy nor loss of sensation followed, but she remained in a state resembling sleep. About the third day, convulsions of the inferior extremities came on; and, on the fifth, coma and death. An enormous effusion of blood was found in the centre of the cerebellum, and considerable fullness of the vessels of the uterus and ovaries. This patient pulled away the limbs when very slightly pinched, and put out her tongue when desired; phenomena incompatible alike with the opinion of those who attribute movement, as of those who attribute sensation, exclusively to the cerebellum.

It was remarked, so early as the time of ARETÆUS, that paralysis took place on the side opposite to that on which a blow of the head had been inflicted; and so general is this, that some (Rouchoux, Serres, &c.) have denied the possibility of the injury and the palsy both occurring on the same side. In a Memoir on this subject, by M. BAYLE,* we find eight examples in which, on examination after death, *the lesion of the brain was found on the paralysed side.* They stand as follows:—1. A blow on the left temple: palsy of the right arm; effusion of blood on the right; and no lesion on the left side of the brain. 2. Comatose affection, with hemiplegia of the right side; disorganization of the cerebrum and cerebellum on the same side. 3. Attack of apoplexy, with hemiplegia of the right side; erosion of the right optic thalamus, and effusion of blood into the ventricles, so as to produce considerable pressure on the right side, and but very little on the left. 4. Attack of apoplexy, with hemiplegia of the right side, which passed off to a considerable extent after a time; in four years, a fatal seizure. Three cavities with apoplectic cysts, effusion of blood into the optic thalamus, and softening of the corpus striatum,—all on the right side. 5. Apoplectic seizure, with hemiplegia of the right side; softening of the right hemisphere. 6. Apoplectic attack, with hemiplegia of the right side; bloody extravasation in the right hemisphere. 7. Mania, with epilepsy; palsy of the left side: rachnitis and softening of the left hemisphere.—

* *Memoir sur l'Existence de la Paralysie du même côté que la Lesion Cerebrale qui la determine,* par A. L. I. BAYLE.—(Revue Medicale Janvier.)

These cases are taken from the works of Smetius, Forestus, Valsalva, Bruner, and Morgagni, the seventh only being his own; and it is remarkable as the only instance in which the injury and palsy both occurred on the left side.

M. Bayle endeavours to explain this phenomenon by supposing that there are some nervous fibres which do not cross. "In fact," says he, "amid the fasciculi of nerves which compose the medulla oblongata, the anterior, which intersect each other before they arrive at the brain, contain some fibres which do not appear to undergo any kind of crossing; while the posterior and lateral fasciculi do not present any manifest intersection."

One of the fashions at present prevailing among the French, is for the elder physiologists to set on some of the younger to write for them, and bolster up their reputation by the repetition of their experiments and the reiteration of their opinions. It appears to be with this view that M. LACRAMPE-LOUSTAU has been made to publish some researches conducted "sous les yeux de M. SERRES." It was a problem laid down for solution by the latter, in his work on Apoplexy,—"a paralysis being given, to determine its seat by the symptoms?" In the Memoir before us we find it asserted, that palsy confined to the arm arises from a lesion of the posterior part of the optic thalamus, and of its posterior radiations; that paralysis of the leg proceeds from injury of the anterior half of the corpus striatum, or of its anterior radiations; that in hemiplegia it is a lesion of the corresponding half of the optic thalamus and corpus striatum, or of the radiations proceeding therefrom. In one case it was the anterior part of the corpus striatum, and posterior of the optic thalamus, which were affected. In short, according as the palsy is more complete in the arm or in the leg, is the alteration more deep and more extensive towards the thalamus or the corpus striatum. Laying these positions down as facts, it is concluded that the posterior radiations of the optic thalamus preside over the movements of the arm, and that the posterior radiations of the corpus striatum preside over the movements of the leg.*

Following the same arrangement as in the preceding department, we next turn to the *circulating system.* Dr. ABERCROMBIE has published a collection of cases of disease of the heart, under the heads of "Inflammatory Affections," "Organic Affections," "Rupture," and "Displacement of the Heart."† These are not devoid of interest, but contain few points of novelty which are calculated for insertion here. Among the organic peculiarities, the following is the most remarkable.

"The left auricle of the heart was very much enlarged, and contained two remarkable bodies. The one was a spherical cyst, about an inch

---

* *Recherches faites a l'Hospice de la Pitie, sous les yeux de* M. SERRES, *pour determiner les Rapports des Lesions du Cerveau avec les Paralysies des Membres, superieurs et inferieurs.* Par M. LACRAMPE-LOUSTAU.—(Revue Med. 1824.)

† *Contributions to the Pathology of the Heart.* By JOHN ABERCROMBIE, M.D. Fellow of the Royal College of Physicians.—(Transactions of the Edinburgh Medico-Chirurgical Society.)

and a half in diameter, full of dark-coloured tenacious fluid. The cyst was externally of a dark-brown colour, nearly black; and, in the fluid which filled it, there were several membranous substances of the same dark colour, like similar cysts in a collapsed state. The other body was a cup, or hollow hemisphere, of a diameter exactly corresponding with the spherical cyst, taking in the half of it. It was about one-third of an inch in thickness at the bottom, and became gradually thinner towards the mouth, where it terminated in a thin margin. It was of a light brownish or ash colour, and a firm fleshy structure, and composed of concentric laminæ, eight or nine of which could be separated from each other at the thickest part of it. It was found lying loose in the cavity of the sinus; but, on the outer surface of it, at the bottom, there was an irregular roughness, as if it had been torn off from some attachment to the parietes of the auricle. The spherical cyst did not appear to have had any attachment. The mitral valves were slightly ossified."

This patient had suffered from severe suffocating cough, palpitation of the heart, and frequent attacks of dyspnœa. The palpitations frequently terminated in a state resembling asphyxia, which continued for a few minutes; she had also occasional temporary loss of sight, in which an object gradually disappeared for a time, as if a cloud had intervened. Dropsical symptoms afterwards came on, and she died from gradual exhaustion. It is remarkable that the pulse continued regular until within a week of her death. We remember a similar case, which occurred in the Royal Infirmary of Edinburgh, about 1812.

Connected with this branch of pathology, we may mention a case of *malformation,* which we owe to Dr. Holmes, of Montreal.* In a man, who lived to the age of twenty-one, notwithstanding that he had been delicate from infancy, and suffered much from palpitation attended with blueness of the lips, it was found that the right auricle had become so much enlarged as to be capable of containing a pint, the musculæ pectinatæ being very strong, and the inner surface feeling gritty, apparently from earthy deposition. No communication existed between this auricle and its corresponding ventricle, but the blood passed from it into the left ventricle by a large opening, furnished with valves similar to the bicuspid. Between the ventricles, a communicating aperture, with tendinous margins, existed, just beneath the semilunar valves of the aorta. The foramen ovale was open. The right ventricle was much smaller than usual; the left much enlarged, and very thin. The course of the blood must obviously have been as follows:—Entering the right auricle by the two cavæ, it would pass into the left ventricle, except a small portion that might get into the auricle; on the ventricles contracting, such portion of the blood on the left side as was not thrown into the aorta would pass into the right ventricle, and hence reach the lungs, to be returned to the left auricle.

A case of *gangrene of the heart* has been put on record by Dr.

---

* *Case of Malformation of the Heart.* By W. F. Holmes, M.D. &c.—(Transactions of Medico-Chirurgical Society, Edinburgh.)

KENNEDY, of Glasgow, which, although described in rather an affected style, is nevertheless possessed of sufficient interest for quotation.

"This disease, in the progress of its ultimate stage, was accompanied by the following manifestations:—Together with an unsteady glistening eye, an indescribable expression of anxiety was depicted on the patient's countenance. Depositions of dark sordid matter on her gums and teeth were incessantly renewed. The surfaces of her tongue, mouth, and fauces, were parched, rough, and black: in the end, the margins of her lips grew quite livid. Her voice was broken and mournful. The dejections consisted of very dark, scybalous, intolerably fœtid substances; her urine was scanty, thick as syrup, and porter-coloured.

"Her pulses, at first small, hard, irregular, rapid, afterwards became feeble and less frequent, and intermittent: long before death, they disappeared altogether from the wrists. Syncopal tendencies often recurred, but never terminated in fainting. A sensation of burning heat pervaded all the regions of her chest; in the left it was excruciating. During the last four days of her life, the patient suffered much from stinging pains, which first occupied her extremities, then pervaded the shoulders and line of the vertebral column, then seized the left thoracic department, and, after the last bleeding, went to be immoveably fixed in the right hypochondriac region. She had catchings in the chest and orthopnœal breathing.

"At an early period, cardiac palpitations supervened, and gradually acquired frequency as well as strength. As the disease advanced, however, and the powers of the heart began to yield, its actions became very intermissive and troubled. All the extremities, superior and inferior, suffered progressive tumefaction from the diffusion of extravasated lymph. The left arm and limb, in particular, were distended almost to bursting: their joints, at first nearly inflexible, could not, during the three days which preceded her dissolution, be moved without occasioning exquisite pain. Broad livid patches, in some places, disfigured their surfaces; on others were large sphacelated wheals. At the same time, she was altogether so impotent as to be incapable of changing, in any degree, her position without assistance. Many hours before her demise, she fell into a state of comatose abstraction, which uninterruptedly increased, till the last of her vital forces irreparably failed.

"Twenty ounces of turbid serum were taken from the chest: it had an impure orange colour and a fœtid smell. The pericardium enclosed four ounces of a fluid in all respects similar. On the internal surface of this capsule was much vascular net-work, dark, as if composed of injected veins.

"All the parts of the heart, external and internal, exhibited distinct marks of having been the seat of gangrenous inflammation. They were preternaturally flaccid, and dark in colour as the darkest coagulated venous blood; they could be easily perforated in every direction with the finger. When thus torn, they exhaled a putrid odour; but no blood exuded from their ruptured vessels. The left ventricle, in particular, was quite livid, and destitute of its muscular tenacity; it was little firmer than cerebral structure. When lacerated, it threw out a most offensive odour, not different from what is generated by putrescent animal substance. All the cavities of the heart were empty; but the large veins, especially the abdominal, were loaded with grumous blood.

"On the thoracic face of the diaphragm and surfaces of the left pleural membrane, were small patches of vascular reticulation: in other parts, they were roughened with darkish granules, having more firmness than those by which structural lesions are repaired. Although heavily loaded with blood, the lungs appeared healthy; they remained free from adhesions of every kind.

"Patches of morbid structure, unequal in size, and discoloured to various shades of darkness, had formed on different parts of the surface of the body; some of them were putrescent, others putrid. Each of the left extremities was in a state of general tumefaction, and firm from distention: around their articulations, and over the central portions of their muscles, the surface had assumed a livid hue."*

Three cases, by MM. Andral and Bayle, are to be found in a recent French periodical, in which the heart was affected with a disease having all the characters of cancer. It is remarkable, but from these cases it would seem that the muscular fibres of that organ may be in part destroyed, without any considerable derangement of the circulation.†

M. Louis has devoted much attention to the history of those cases in which a communication exists between the right and left cavities of the heart, and the following are the results at which he has arrived:‡—This communication may exist in a variety of ways, but the most common are the foramen ovale, and the perforation of the interventricular partition. It is congenital; is associated, in the majority of cases, with a well-marked narrowing of the pulmonary artery, and always with dilatation of one or more of the cavities, generally with those of the right side. The effect of this communication is a mixture, more or less ample, of the black and red portions of the blood; but the blue colour of the surface is rarely universal: sometimes it is only preserved in the face during the last few weeks of the patient's life, and occasionally is not perceptible at all. This communication, and consequent mixture of venous and arterial blood, may exist a long time without the health being affected, and many of the characters, as the discolouration and the sensibility to heat and cold, and sense of suffocation, are frequently wanting. The only pathognomonic symptom, according to M. Louis, is frequent fits of suffocation, which often come on periodically, and are excited by the slightest causes. The disease is neither incompatible with long life, nor with development of the intellectual faculties. These remarks are in conformity with general observation.

Proceeding to the history of particular tissues, we find that M. Andral has published a Memoir on *inflammation of the pleura cover-*

* *Medical Repository*, April. † *Revue Medicale*, Fevrier 1824.

‡ *Observations suivies de quelques Considerations sur la Communication des Cavités droites avec les Cavités gauches du Cœur.* Par M. Louis.—(Arch. Gen. de Med. Novembre 1823.)

*ing the diaphragm*,* of which affection he has seen many examples, either simple or combined with inflammation of the other parts of the pleura, or of the substance of the lungs. Without following him through the history of individual cases, we subjoin some of the conclusions at which he arrives with regard to the characteristic symptoms: among these he ranks a pain, more or less intense, along the margin of the false ribs, extending in general to the hypochondriac region, and sometimes to the loins; complete immobility of the diaphragm during inspiration; remarkable anxiety, evinced by sudden alteration of the features; orthopnœa, with inclination of the body forwards. Other symptoms, such as hiccough, nausea, vomiting, convulsive movements of the muscles of the face, delirium, and finally jaundice, where the pleurisy affects the right side. M. Andral regards the *paraphrenitis* of Boerhaave as having been in reality diaphragmatic pleurisy.

A Memoir on *the pathological anatomy of the peritoneum*,† by Dr. Scoutteten, contains many instructive remarks, particularly with regard to inflammatory affections of that part. A good account of these has been given in the last Number of a respected contemporary Journal,‡ which we shall here transcribe into our pages.

"1. *Peritonitis Acuta.*—Our author thinks, and with justice, that it is needless to give various names to peritonitis, according to the portion of peritoneum which is inflamed, as omentitis, mesenteritis, &c. It is important, no doubt, to ascertain, as nearly as possible, in what portion of the membrane the focus of inflammation is placed, merely that the external means of relief may be there applied; but, to give names to these, is worse than useless.

"When irritation has been determined to the peritoneal membrane, and there produced the mildest shade of inflammation, we observe small red spots, not more than a line in diameter, separated from each other, and, on minute examination, appearing to be clusters of puncta crowded close together. Between these red spots, the peritoneum, when viewed with a good magnifying glass, presents portions of its surface of a natural colour. The appearance now described is rarely to be seen in man, for obvious reasons; but it may be readily produced in animals, as dogs, by injecting an irritating fluid into the cavity of the peritoneum. Thus, if bile be thrown in and the wound closed, the above appearances will be strikingly produced in twenty-four hours.

"It has been remarked by several acute observers, that all the symptoms of peritoneal inflammation have been present during life, and yet on dissection no trace of it could be found on that membrane. On this point our author professes to be sceptical. But we think there can be no reason to doubt that mere injection of vessels, or the first stage of inflammation, where the structure of the tissue is not altered, may disappear in the interval between death and dissection. Indeed, our author's own experiments give the greatest support to this opinion. Into

---

* *Observations sur l'Inflammation de la Pleurè Diaphragmatique.* Par Andral, fils, M.D.—(Archives Gen. de Medecine, Octobre 1823.)

† *Archives Generale de Medecine*, Dec. 1823, Feb. 1824.

‡ *Medico-Chirurgical Review*, June.

the peritoneal cavities of dogs he injected bile, and, at the end of twenty-four hours, examined the abdomen, when inflammation was unequivocal. The animal was then immediately killed by pithing, and an evident diminution of the inflammation took place with death. As the dogs got cold, the diminution was still greater. These experiments were repeated many times, and always with the same result. It is therefore highly probable, if not quite certain, that the first stage of peritoneal inflammation, in some particular constitutions, may produce such disturbance in the vital functions as to destroy life; and that the collapse after death may dissipate the redness and other marks of phlogosis which existed anterior to that event.

"In the early stages of inflammation, our author found by experiment that the surface of the peritoneum, though apparently dry and glistening, was, when touched with the finger, covered with an unctuous and viscid exudation. Sometimes, instead of the red spots above described, this first shade of phlogosis presented merely a development of red vessels running in lines to a greater or less extent.

"In the progress of the inflammation, the red spots become more extended and close together,—ultimately so blended as to appear one homogeneous patch, of a scarlet colour. Still at this period the distended vessels are visible, but the peritoneal tissue does not appear to be thickened. It has, however, by this time lost its transparency. When the phlogosis is still more advanced, the redness becomes more intense and extended: sometimes occupying the whole peritoneal expansion; at others, bounded to the forms of bands or stripes, traversing various portions of intestine, or only occupying the space where the intestines adhere to each other. The intense red colour at this period is not wholly owing to the injection of vessels, but to a sanguineous exudation which is diffused over the peritoneal surface, adhering very tenaciously to it, and presenting a villous appearance. Even in this stage the peritoneum will sometimes appear dry and shining; but, more commonly, we now find an effusion of a whitish fluid into the abdominal cavity.

"Such an acute inflammation of the abdomen can only last, our author thinks, a few days, (three or four,) without death or a change for the better. The abdominal pain is generally very acute,—the patient drawing up the thighs, or bending the body forwards, to relax the abdominal muscles. Sometimes the pain is bounded to a single point, and then we generally find the inflammation also bounded to the same spot. On the 3d September last, our author opened a man who had felt acute pain in the right iliac region for two days prior to his death. He found, on dissection, the marks of intense inflammation of the peritoneum bounded to the appendix cæci. At times, the peritoneum covering the bladder will be the seat of phlogosis, and then the evacuation of the urine will be almost uniformly suspended, and the pain felt in the pelvis. The peritoneum covering the inferior surface of the diaphragm may be the sole seat of inflammation, and then we have almost constant hiccup. A soldier in the Val de Grace Hospital experienced, for some days, an intense gastro-enteritis, which began to give way to the usual means, when all at once a violent pain was experienced (augmented by pressure) in the direction of the diaphragm, accompanied by constant hiccup, shrinking of the features, drawing up of the legs and thighs. These symptoms could not be controlled, and he sunk in two days. M. Broussais prognosticated the existence of sub-diaphragmatic inflammation of the peritoneum, which was completely verified by dissection.

" In acute inflammation of the peritoneum, we have this membrane sometimes of a purple or even black colour, with strong adhesions of the folds of intestines together, without the intervention of a false membrane, which at other times is occasionally, indeed often, seen. Actual gangrene of this membrane has been found after death; but this is of rare occurrence.

" Another phenomenon, of still rarer occurrence, presented itself to our author in the year 1822, in the body of a man who had died of acute peritonitis. This was a subperitoneal emphysema. The whole sheet of this membrane was equally elevated by gas, which could be pressed from place to place, and made to accumulate in particular positions. In another case he found the same phenomenon, but on a much more limited scale, the emphysema being bounded to the peritoneum lining the diaphragm and covering the liver. In neither of these instances was there any sign of putrefaction to account for the collection of gas.

" Between the laminæ of peritoneum forming the mesentery, we sometimes find collections of purulent matter, in peritoneal inflammation. This, however, is not of frequent occurrence.

" When peritoneal inflammation is extended to twenty, twenty-five, or thirty days, false membranes of albuminous matter are found gluing the convolutions of the intestines together, and even these last to the peritoneum lining the abdominal parietes.

" During the first few hours of acute peritonitis, there is very little effusion of fluid beyond the usual halitus; but, after the inflammation has lasted thirty-six or forty hours, there will be an evident effusion, generally of a whitish or milky appearance. Blood itself has been found extravasated in acute peritonitis; but this is a rare phenomenon. The quantity of effusion varies from a few ounces to some pints. Sometimes it is nearly as limpid as water, and containing no albuminous flocculi; in other cases it is thick, and resembling diluted pus of a very peculiar odour, which can never be forgotten by those whose hands have been imbued in it.

" 2. *Alterations of structure observed after chronic inflammation of the peritoneum.*—Chronic is often the consequence of acute peritonitis; but it also not unfrequently creeps on in a slow, and almost insensible, manner, without any violent symptom. This last may be properly termed ' primitive chronic peritonitis.'

" *Chronic after acute peritonitis.*—On opening the body of a person who has had peritonitis for fifty or sixty days, we shall find the abdomen containing a greater or lesser quantity of a whitish fluid; a number of false membranes gluing the intestines together, or forming sacs which contain fluids of different appearances. When these false membranes are detached from the peritoneum, we shall find that structure less red than in acute peritonitis,—sometimes, indeed, scarcely coloured. In these cases, the effused fluid is rarely in such quantity as to sensibly distend the parietes of the abdomen. In some cases, however, a considerable quantity of limpid yellow serum will be found, without any trace of false membranes, but with the peritoneum thickened, reddish, and highly injected; or the great omentum thickened, red, fleshy, or presenting hydatiform bodies. These two shades of peritonitis are not attended with much pain, and bear abdominal compression without much inconvenience. The patients only complain of a sense of weight, and they are much harassed with constipation of the bowels. When chronic peritonitis has lasted many months, in certain constitutions, we find, besides numerous adhesions and false membranes, a development of tubercles, of different sizes, and various degrees of consistency.

"Instead of the serous or puriform fluids which we generally find after peritoneal inflammations, there is sometimes, though rarely, an extravasation of sanguinolent, or even pure sanguineous fluid, resulting from rupture of vessels.

"Of ulceration, gangrene, and scirrhus, of the peritoneum, we shall not speak, as they are extremely rare in their occurrence.

"The above are the general appearances presented after inflammation, acute and chronic, of this important membrane, and the practitioner should bear them in mind when he is prosecuting his post-mortem researches."

In our last Number we laid before our readers a brief notice of Dr. DUNCAN's opinions with regard to those serious, and even fatal, consequences which occasionally follow slight wounds received in dissection, or from simple venesection. A very striking example of what he has called *diffuse inflammation of the cellular membrane*, attended by some anomalous symptoms, remains to be mentioned in this place. A woman, aged thirty, fell under Dr. Duncan's care, in September 1821, labouring under symptoms which were regarded as the fever at that time prevailing epidemically in Edinburgh. After a few days it was perceived that the right arm and right side of the thorax were affected in a manner similar to what occasionally results from venesection; and it is proper to observe that the patient had been bled, the lancet-wound healing by the first intention. From this time the symptoms increased, notwithstanding the administration of an emetic, general and local blood-letting, and strict antiphlogistic regimen. After a few days she began to mend, and continued alternately recovering to a certain extent, and again relapsing. During one of these accessions of fever, she complained of severe pain of the right side, increased on pressure; the side was swollen, and a tumor, about the size of a pigeon's egg, was perceived above the right clavicle. Soon after she was attacked with some cough and free expectoration; at the same time the external tumors began to soften, and ultimately became emphysematous,—a gurgling noise being heard as the air rushed into them during respiration. As the breathing became much more oppressed whenever the expectoration diminished, it was judged advisable to make an opening into the side, so as to give a free vent to the pus as it formed. This was accordingly done by Mr. WISHART; when a blast of air was discharged by the wound, in addition to the pus. From this time she scarcely brought up any matter by expectoration, but gradually sunk, and died on the

3d of December, being rather more than three months from her first admission into the hospital. On examining the body, very extensive abscesses were found on the breast and neck, communicating with the lungs; so that the case presents a remarkable instance of circumscribed emphysematous tumors, in which the air was confined in cysts bounded by adhesive inflammation.*

Dr. ABERCROMBIE, continuing to prosecute his pathological researches, has published some observations on inflammatory affections and ulceration of the stomach, on diseases of the pylorus, and of the pancreas.† In regard to the *stomach*, the author insists much upon the insidious nature of the symptoms which accompany chronic inflammation of this viscus, and dwells on the importance, and at the same time the difficulty, of distinguishing the complaint from simple dyspepsia. The progress of the inflammation has appeared to Dr. Abercrombie to be extremely slow, occasionally subsiding and recurring, until at length it gives rise to thickening of the coats of the stomach, to adhesions, and to ulceration. These papers may be consulted with advantage, as they evince considerable research, and contain frequent references to similar cases recorded by continental writers, especially among the French; yet they do not present many features of absolute novelty, the most interesting cases, if not the greater number of the whole, being borrowed from others. The following case, however, we think it advisable to quote, as one of the most extensive examples of ulceration of the stomach on record.

"A lady, aged forty-nine, had been in bad health through the winter 1811-12, complaining chiefly of general weakness, and a constant uneasiness across the epigastric region, with occasional attacks of acute pain towards the left side. In May, 1812, she began to be affected with vomiting, which continued from that time, and became more and more urgent. I saw her in July, and found her much emaciated and reduced in strength, so as to be confined to bed. She complained of an obtuse pain in the epigastric region, where considerable hardness was felt; and she vomited a portion of every thing she took, sometimes immediately after taking it, and sometimes a considerable time after. The pulse was weak, and rather frequent. She continued with very little change till the beginning of September, when the vomiting subsided, and she was completely free from it for more than a fortnight. During this time she was affected with diarrhœa; her strength sunk, and she died on the 23d; the vomiting having returned three or four days before her death, though with less severity than before. During the period when she was free from vomiting, she took food and drink of various kinds, and in very considerable quantities, and continued to do so till a few hours before her death.

"*Dissection.*—On opening the abdomen, and looking for the stomach, a large irregular opening presented itself, which was found to

---

* *Case of Diffuse Inflammation of the Cellular Substance of the Side, &c. &c.* By ANDREW DUNCAN, jun. M.D.—(Transact. Med.-Chir. Society, Edinburgh.)

† *Edinburgh Medical and Surgical Journal*, January 1824.

lead into the cavity of the stomach, a large extent of the great arch being entirely destroyed. On the left side, in the region of the spleen, there was found a large irregular mass, which appeared to consist of an enlarged and diseased spleen, and the remains of the great arch of the stomach, so blended into one mass, that it was impossible to distinguish one part from another. In the substance of it there was a cyst, full of very fetid matter. This mass was attached to the cardia by a narrow neck, which remained of the coats of the stomach at that place; and, when the parts were taken out, and displayed by suspending the stomach by the cardia and the pylorus, the appearances were very remarkable. When stretched out in this manner, about one half of the stomach, at the pyloric extremity, was sound and healthy. This portion was attached to the cardia by a narrow portion of the small curvature which remained; and, by another small portion of the great curvature, the mass now referred to hung down on the left side. The remainder of the stomach, consisting of the left side and lower part of the great arch, was entirely wanting to such an extent, that, when the parts were extended in the manner now mentioned, it appeared that nearly one half of the stomach had been destroyed. There was reason to believe that the part which seemed to be wanting was involved in the diseased mass on the left side, and that the sound parts had been separated from this portion by a line of ulceration of such extent, that the sound extremity remained attached to the cardia only by a portion of about two inches in breadth, which remained of the small arch. The ulcerated edge, where the separation had taken place, was studded with numerous hard tubercles, like the edges of a cancerous ulcer.* The pancreas was hard, the liver pale and soft; the other viscera were healthy."

The diseases of the *pylorus* related by Dr. Abercrombie are principally cases of schirrus; the most remarkable of which is the history of a patient, who had none of the symptoms characteristic of this disorganization. During the last twelve months of his life he had no complaint, except progressive wasting and debility; there was no vomiting; the appetite was tolerably good, and the bowels regular: in short, he had no symptom except repeated attacks of violent pain in the abdomen. On dissection, " a large mass of schirrus, four or five inches in extent, surrounded the pylorus; and the pyloric orifice was so narrowed as scarcely to admit the point of a very small finger. The inside of the mass opened upon the inner surface of the stomach, by an ulcerated surface, covered with large cancerous looking tubercles. The other parts of the stomach were tolerably sound, and the other viscera were healthy."

Four cases of disease of the *pancreas* are mentioned by Dr. Abercrombie, and seem to have been of a cancerous nature. He regards disorganization of this organ as one of the causes of anæmia: he was particularly led to this opinion from the following remarkable case.

" A gentleman, aged thirty-five, died, after an illness of about eighteen months' duration, in which it was, to the last, impossible to say

---

* Some interesting cases of perforations of the alimentary canal are to be found in the *Archives Generales de Medecine*, Jan. 1823.

what was the seat of the disease. His complaints began in London with a febrile attack, which left him very weak. From that time he was liable to dyspeptic symptoms, with variable appetite, and undefined uneasiness in the epigastric region. Without any other complaints, he gradually lost flesh and strength; and, when Mr. Newbigging saw him, in January 1822, he found him thin and weak; but he was particularly struck with his remarkable paleness,—even his lips, and the inner surface of his mouth, being entirely colourless. About this time he had some vomiting, and was feverish for a day or two; but these symptoms soon subsided, and left him, as before, with variable and capricious appetite, and an irregular state of the bowels, which were sometimes costive and sometimes loose. He had frequent perspirations in the night-time, and appeared at all times languid and faint; but his pulse was natural, he took a good deal of food, and there was no symptom that accounted for his exhausted appearance. In January he seemed to improve considerably; but in February he became worse, being affected with diarrhœa, and his urine became scanty, and deposited a mucous sediment. These complaints, however, soon subsided; and he then complained chiefly of throbbing in the head, and a constant noise in the left ear. When I saw him, in the middle of April, he was reduced to the last degree of paleness and debility; but his pulse was full, strong, and regular. He took a good deal of food, and complained of nothing except a painful pulsation in his left ear. He died in the end of April, without any change of the symptoms, except that his pulse became frequent a few days before his death; but it continued full, and rather strong. The action of the heart was rather violent, and seemed to produce throbbing over the whole body.

"On dissection, all the internal parts were found remarkably pale and void of blood. The heart was sound, but remarkably empty. The pylorus was thickened and firmer than natural, and had contracted an adhesion to the pancreas. The pancreas was considerably enlarged, and was diseased through its whole structure, being in general hard and cartilaginous; but in some places soft, with the appearance of the medullary sarcoma. All the other organs were healthy."

We now turn to some points touching the pathology of the *fluids*.—An extensive series of experiments, conducted with the utmost care, and showing the remarkable advantage of being directed to a distinct and useful end, have been recently performed at the Hôtel Dieu, under the superintendance of M. Recamier.* The first experiment will at once illustrate an important general fact, and show the minute attention displayed by M. Belhomme, by whom the observations are recorded. "Two bleedings were carried on at the same time, the vessels used being the shape of inverted cones. Robillard, a man aged thirty-five, of athletic constitution, had made a violent exertion, in consequence of which he suffered in the lumbar region. He was seated. *Right arm*,

* *Observations faites a l'Hôtel Dieu, pendant l'Année* 1823, *sous les Yeux de* M. le Professeur Recamier, *sur le Sang et la Couenne Inflammatoire*, par J. E. Belhomme; *precedées de Reflexions generales*, par A. Duges.—(Revue Medicale, Mars 1824.)

—opening in the skin one line and a half, in the vein one line; jet of little strength, continuous for three inches, and lasting three minutes. *Left arm*,—opening in the skin two lines, in the vein one line and a half; jet very strong, rapid, continuous for seven inches, and lasting two minutes. Result: *Right*, no buff; clot of the ordinary consistence. *Left*, very thin layer of buff; clot and serosity as on the right."—By this experiment it is shown, that the blood flowing from a large opening, with a powerful jet, afforded a buffy coat,—slight, it is true, but yet distinctly marked,—and which was entirely wanting in that which flowed from a narrower aperture and less forcible jet. This circumstance held good as the general result in a numerous series of experiments. All the observations of M. Belhomme were conducted in the same minute and careful manner. Convinced that the blood always participates in the alterations of the solids, he devoted particular attention to its condition in patients labouring under various diseases, and in pregnant women; but, equally persuaded of the influence of certain accidental circumstances in producing certain appearances, he very properly thought it necessary to begin his examination with persons, either in health or but very slightly indisposed. He found that, in all, certain states favour the formation of the buffy coat:—viz. 1st, an aperture of moderate size; 2d, a rapid and continuous jet; and 3d, receiving the blood in a narrow vessel. With a view to greater accuracy, he made exact measurements of the vein and of the skin, of the jet of blood, the time during which it flowed, and the vessels into which it was received. In the language of M. Belhomme, an aperture is said to be moderate when its length is a line; large, when two lines; and very large, when three. He points out the necessity of distinguishing between the opening in the skin and in the vein, the former being always larger. He found that the materials of which the vessels were made, had no influence on the phenomena presented by the blood which they contain; but, in general, the one last filled had a more distinct buffy coat.

After having ascertained the influence of external circumstances, M. Belhomme inquired into the effects of physiological and pathological causes. On this subject he informs us, that, even in the healthy state, the buffy coat is generally formed under the circumstances we have above alluded to as favourable to its development. In pregnant women, as is well known, there is a general tendency to this phenomenon; but a remark which, so far as we know, is peculiar to M. Belhomme, is the existence of a peculiar odour in the blood of pregnant women, exactly resembling that of the placenta, when recently expelled. This phenomenon, which is stated to be constant, might be of use in cases of doubtful pregnancy. In subjects labouring under inflammatory and febrile attacks, a great disposition to the formation of the buffy coat was manifested; but even here it was modified, or overcome, by external circumstances. A patient, who had a relapse of inflammation of the chest, with expectoration of blood and much fever, was bled, in the sitting posture, in both arms: one vein was very large, and the aperture made into it was three and a quarter lines in length, from which the blood flowed in a rapid continuous stream: on this a very firm buff formed, between five and six lines thick. On the other side, the aperture in the vein was only half a line, from which a fine but rapid jet flowed; but no buffy coat resulted. It is true that analogous observations have been made before, but they are more precise and satisfactory in the Memoir before us than in any preceding one with which we are acquainted.

Some observations of an interesting nature on the pathology of the blood have likewise been made in this country, by Dr. STOKER,* of Dublin, and Dr. SCUDAMORE.† These we shall here extract, with a view of laying before our readers all that has recently been done on this subject.

The former of these gentlemen says,—"The explanation for some time given of such appearances on the blood, (viz. the buffed surfaces,) is incompatible with these facts, thus detailed, and the opinion involved in that explanation, that slow coagulation was caused by the violent agitation the blood had undergone previously to its being drawn, may perhaps be also questioned. Instead, therefore, of attributing these appearances of the surface merely to the subsiding of the red particles during the slow condensation of the lighter parts, we are, I think, warranted in supposing that an altered and unhealthy state of the blood, exceeding the effects of mere agitation, takes place in the course of circulation, either from want of due preparation of the fluids at the two chief sources of supply, and of the subsequent changes these fluids should undergo in their passage through the pulmonary, sanguiferous, and hepatic systems, or from the injurious effects of diseased functions in the organs of sanguification.

"The colour and external characters which designate various kinds of buffy coat, being also found to indicate the particular functions engaged in producing them, afford additional arguments in favour of the foregoing opinions. In simple pneumonia, for example, the coat on the blood is generally of a colourless white; but, when tinged, it is with bright red, the depth of the tunic seldom exceeding a few lines, and to this probably it is owing that the cupping on the surface of such blood is generally remarkable; the thin and tenacious film contracting as it forms, and drawing towards the centre the external margin at the circumference of the less contractile crassamentum.

"In simple forms of hepatic disease, on the contrary, the buffy covering is generally darker through its whole substance than in pneumonia, and is externally yellow. It occupies a large proportion of the solid part of the blood, and is not often cupped: when it is cupped, there is reason to suppose that the lungs are partly engaged.

"In diabetic complaints, which there is so much reason to believe originate in imperfect digestion, or insufficient preparation of chyle, it is well known that, when blood is drawn, it is often found covered over with a whitish milky-like fluid.

---

* *Pathological Observations. Part I. on Dropsy, Purpura, and the Influenza, &c. &c.* By WILLIAM STOKER, M.D. &c.—Dublin, 1823.

† *An Essay on the Blood, &c. &c.* By CHARLES SCUDAMORE, M.D. F.R.S.—London, 1824.

"I am the more desirous to direct the attention of medical observers to these circumstances, as, if I judge rightly, they will be found to afford effectual aid, not only in reasoning how the surfaces in question were produced, but also in discriminating between the organs affected, with a view to remedy. The observations, indeed, which have been made here, being confined merely to the surface of the blood, embrace but few of the advantages in pathological investigation, which, I conceive, a more general attention to the condition of all the parts of the serum and coagulum would contribute.

"In a conversation which I lately had the pleasure of holding with Mr. Todd, professor of anatomy and surgery in our College of Surgeons, I learned with satisfaction that the opinions which led to the foregoing observations on the diagnostic characters of the buffy coat, coincided with his extensive experience; and he stated to me a fact which appears to me highly important, that, in passing through the wards of the hospitals of the House of Industry, to which he has been for many years surgeon, he could, on inspecting the cups of blood taken in different diseases, be able frequently to pronounce what organs were primarily or chiefly engaged in those who were bled; that with the white and cupped surface having indicated the lungs to be the seat of disease, and that with the dark yellow colour and equal surface, the liver.

"From the difficulty of ascertaining any infallible diagnostic between certain forms of pulmonary and hepatic diseases, (which have so many symptoms alternately or in common,) the foregoing observations will, if found applicable in practice, be justly appreciated.

"In making these remarks on the various kinds of surfaces presented by blood under certain circumstances, I do not wish to be misunderstood as concurring in the opinion that the buffy coat is always indicative of inflammation,—an opinion which does not accord with my experience: on the contrary, I have often witnessed much of that sort of appearance on the blood drawn in certain kinds of dropsies, when the patient had neither increased action of the vessels, nor any unusual sensation of pain or heat in any part; but, on the other hand, in certain conditions of the mucous lining of the cavities or of the viscera, when all other characteristics of inflammation were present, no buffy coat appeared on the blood after being drawn.

"I am inclined, from what I have seen in such cases, to think that, when inflammation is confined to the mucous membranes, the blood is not generally buffed; and that when it is in a sizy state, that then the texture of the liver or lungs is directly engaged, or affected by sympathy with the parts concerned, so as to influence the condition of the functions in these organs."

Dr. Scudamore informs us, that—"It may be stated as a general rule, that blood coagulates in the shortest time, accordingly as it is of high specific gravity. Such is the blood of a strong and healthy person, as abounding most in red particles, which constitute the heaviest part of the blood. Mr. Hunter, in speaking of the red globules, remarks that 'their use would seem to be connected with strength, for, the stronger the animal, the more it has of the red globules.' The fibrin, also, belonging to such healthy blood is more dense than that of blood in disease; and hence the real explanation of a quick coagulation.

"The very marked difference of time in which the blood coagulates, accordingly as the stream from the orifice is fast or slow, appears to me to warrant the conclusion which I have stated, that the commencing part of the process of coagulation,—namely, the escape of carbonic acid, does take place more readily when the blood flows very slowly.

"It appears that rest, merely, does not assist the coagulation of the blood. We see in how remarkable a manner cold delays coagulation of the blood in the basin; and how slowly it takes place when confined in a vessel of the living animal.

"The proportion of fibrin and its quality, as having an influence on the coagulation of the mass, is a point of material consideration. We must keep in view that the solidification of the fibrin is the essential cause of coagulation. The process is promoted by heat, 'for two reasons.' The carbonic acid is driven off sooner, and the solidification of the fibrin is evidently assisted. The temperature required to render the fibrin almost immediately solid, varies according to the density of the fibrin. Thus, in an example of moderately sizy blood, collecting the fibrin while liquid and mixed with the serum, I found that it required a heat only of 120° to render it solid, and the serum 140°. The density of this blood was but little below that of the most healthy blood, for the patient was bled for the first time; but in another example, in which the fibrin very greatly predominated in quantity, the same degree of effect required a heat of 130° for the fibrin, and of 160° for the serum. This blood was of very low specific gravity. The patient had been bled several times, and was much reduced.

"In attempting to explain the coagulation of the blood, I should be induced to say that it depends on a new condition of the fibrin, which can only exist fluid in a state of intimate mixture with red particles, serum, and carbonic acid gas. Except in circulation or in living vessels, the fibrin will not maintain an union with the serum, although it does continue blended with the red particles, either when extravasated in the living body, or when taken from the vessels. It is therefore the property of the fibrin to become solid when separated from the several principles just stated, constituting in union the mass which we call blood. It will perhaps be asked, how does it happen that such blood as contains the largest quantity of fibrin coagulates the most slowly, when coagulation depends upon the fibrin? I do conceive, that the larger the proportion of fibrin is in healthy blood, as its proper constituent, the more rapid will be its coagulation.

"I wish to observe, also, that such blood, which I advert to as the most healthy, contains the largest proportion of red particles, as may be deduced from its high specific gravity. Indeed, in blood of perfect health, its constituent parts may be supposed to exist in a constant relative proportion to each other, and that every considerable difference from such proportions ought to be viewed as a deviation from the best standard.

"To the circumstance chiefly of a large proportion of fibrin as belonging to health, I am disposed to attribute the immediate coagulation of the blood of the sheep; in which I have found the proportion of fibrin to 1000 grains of crassamentum 7.29 grains. The mean proportion in eleven examples of human healthy blood, was 3.72 grains. In answer to the question just now proposed, I contend that, when the fibrin is in great excess, it is less dense than in healthy blood, and takes the solid form more slowly. As explained in my experiments, the excess is so abundant in very sizy blood, that the larger proportion remains in the upper part of the basin, in admixture with the serum; and hence its slower coagulation. Also that portion which falls down with the red particles is less dense than natural, and coagulates rather slowly. When the fibrin is in great excess, it would seem that the red particles cannot blend themselves with the whole quantity as in ordinary coagulation.

"The colour of the blood as it flows from the vein, is influenced by different causes. If the ligature have been kept on the arm for some time, the blood which first flows will appear very dark. In obstructed respiration, particularly in a fit of asthma, the blood presents an almost black appearance, in consequence of its difficult transmission through the lungs, and consequently imperfect oxygenation. In quick respiration, as in phthisis pulmonalis, the blood drawn usually appears very florid. I believe that, in all inflammations, the blood drawn from the veins is more florid than natural, unless the circulation is obstructed, so that the blood passes with difficulty from the arteries to the veins.

"We may soon judge of the density of the blood on its being received into the basin. The appearance to the eye gives some information, but we are chiefly instructed by the time in which it coagulates. Under the head of *specific gravity*, I have given examples to show that the heaviest blood coagulates the most quickly. It is well known that blood which will furnish the buffy coat in a great degree, in the course of a few minutes presents a watery appearance, and the finger, being applied to the surface, is not stained with blood. We may also notice, before the blood becomes quite coagulated, whether it has a frothy appearance, as indicative of a large proportion of fixed air, taking into the account that, if it has passed in a full projecting stream, more atmospherical air will be entangled than if it flowed down the arm.

"I believe that these are the chief circumstances to be noticed in the immediate appearance of the blood, when passing, and just received into the basin.

"The blood should be received in different cups; and the breakfast-cup is the most suitable size. They should be put by in order, all in the same situation as to temperature, and not disturbed, so that we may be enabled to draw some useful inference of the effect produced on the circulation during the operation of venesection, by the relative appearances of the different portions of blood. In deciding upon the mere presence or absence of the fibrinous coat, we must particularly refer to the quick or slow stream in which the blood has flowed. In active inflammation of the fibrous textures, the formation of the buffy coat cannot be prevented, because the fibrin in the blood is in such excess; but, when inflammatory action of the circulation does not much prevail, we shall learn more of the nature of the blood from examining its texture, than from merely viewing the surface of the coagulum. I consider that the texture of the coagulum, and its degree of contraction, indicate more of the actual state of the heart and arteries, than the mere presence or absence of the fibrinous coat. In the examination of blood taken away by cupping, we have to form our judgment chiefly from the texture of the coagulum; as to the eye, it almost constantly presents an uniform appearance. In general terms, I may state, that a firm texture of the blood points out a strong action of the blood-vessels, so as to give a presumptive sign that the bleeding has been proper; and, vice versa, if the coagulum be remarkably loose in texture, we should particularly question the propriety of repeating the operation. I may further remark, that, when the clot possesses uniform firmness, and has its edges turned inwards, we may conclude that the blood-vessels act more strongly than when it is soft in its texture throughout, and having a thin and flabby edge. The extreme toughness of the buffy coat itself, and this contrasted with the soft texture of the inferior part of the coagulum, is matter of familiar observation.

"The quantity of serum which appears in the basin depends chiefly on the more or less firm contraction of the clot. It most abounds when the blood presents the buffy coat, because the strong contraction of such a clot forces out the serum.

"Respecting the external appearance and qualities of the serum, I am not aware that very material conclusions are to be drawn. Its density cannot be judged of by the colour.

"Experiment 84 shows that the palest serum had the highest specific gravity. When the bile has found its way into the circulation, it tinges the serum strongly. If a person be bled shortly after the dinner meal, the serum appears milky. I believe that this appearance is owing to the recent introduction of chyle into the circulation; and it seems to me to warrant the idea, that several rounds of the circulation take place before sanguification, or the complete conversion of chyle into blood, is effected. * * *

"It is undoubtedly true, as a general circumstance, that in the same proportion that the buffy coat of the blood appears to abound, the clot being remarkably cupped, as it is expressively called, with edges inverted, so does the inflammatory diathesis prevail; and, indeed, short of such strongly-marked evidence, in those cases in which the signs of internal inflammation are obscure, the appearance of the blood, as exhibiting the buffy coat or being wholly free from it, or in its texture, as being remarkably firm or the contrary, very materially serves to assist our diagnosis. In forming such diagnostic opinion, I must again desire that reference be made to all the circumstances which have an influence on the particular formation of the coagulum.

"An increased rate of the circulation, merely, does not occasion a larger proportion of the fibrin to be found in the blood, as is proved by Experiments 83 and 84. On the contrary, indeed, in this example, the largest proportion of fibrin was found in the blood drawn before the exercise. The increased proportion of serum, together with its higher specific gravity, concurring with the diminished proportion of fibrin, is a fact worthy of observation.

"It requires the continuance of disease to give rise to the buffy coat. It will not be found in blood drawn in the first few hours of inflammatory action. I have just now mentioned the negative effect of exercise; but, also, it seldom happens that the blood is sizy in simple continued fever, or certainly not in the beginning of the fever. We may expect to find this condition of the blood in those diseases which are attended with muscular wasting; for example, in diabetes, and, above all, in phthisis pulmonalis. Dr. Watt, in his work on Diabetes, mentions the remarkable prevalence of the buffy coat in this disorder; and, probably, was on this account encouraged in his views of treating diabetes by frequent bleeding. In the early period of pregnancy, the blood usually presents some degree of buffy coat; and, indeed, it has frequently been regarded as a diagnostic of pregnancy. I attribute its appearance to the degree of muscular wasting which takes place under these circumstances; agreeably to my former explanation, of the fibrin being retained in excess in the circulating blood, instead of being distributed in the usual proportion to the muscular fibre.

"It deserves further discussion, how far the appearance of the buffy coat is to be taken as a fixed practical rule for the use of the lancet? It appears to me that, while, on the one hand, it is to be received as strong evidence of the inflammatory diathesis, we must, on the other hand, be careful not to be too much influenced in the repetition of the venesection by such appearance of the blood. In many diseases, it will continue to predominate under circumstances of such great debility, that it would be highly injurious to pursue the practice of bleeding. Even to the dying hour of the consumptive patient, the blood does not fail to exhibit the buffy coat, more or less considerably. In the treatment of severe pleurisy, its continued appearance does not alone warrant incessant bleedings. We must always make a comparative estimate between the action of the vessels arising from disease, and power of the constitution. Whatever may be the medical treatment, a little time is required to allow a change of diathesis to take place; and although, during the state of active symptoms, the free use of the lancet is the *sine qua non* of practice, we are not to consider that the taking away blood constitutes the only means by which we can alter its condition; or, more properly, by which we can remedy the disorder of the system. Medicine, suitable diet, repose, and a little time, will accomplish much. In prescribing venesection according to real symptoms and circumstances, we shall most probably avoid error; and it is incumbent on us to take a careful guidance from the mere continued appearance of the buffy coat. I repeat, that it will frequently be found connected with such serious debility of the constitution, that, to continue to rob the vessels of blood on account of its being sizy, would be dangerous to the life of the patient.

"It is obvious that we are led to the further use of the lancet when the buffy coat of the blood appears, because it is so commonly associated with inflammatory action; but otherwise, according to the explanation which I have attempted, that the excess of fibrin in the blood is simply owing to a suspension of its ordinary distribution to the fibrous textures, we should not, in a theoretical view of the question, consider depletion from the vessels as indispensable. A very free and continued employment of bleeding in acute rheumatism is seldom a successful practice, although it is notorious that every cup of blood would present the appearance of the buffy coat."

M. BELLINGERI has made *the state of the blood, with regard to its electric phenomena* in different diseases, the subject of an Essay,* in which he states, that in the phlegmasiæ there is an evident diminution of electricity, which again returns as the disease loses its intensity; while the contrary holds good with regard to chronic affections. The blood, as it flows from the vein, does not always evince positive electricity: on the contrary, in some cases of severe inflammation, it becomes negatively electrified; and, where a buffy coat forms, there the electricity of the blood, as it flows, will be found inferior to what it is during health. But the buffy coat never forms in any case where there is a degree of electricity superior to that which belongs to the state of health: when the buffy coat does form, the blood retains the degree of electricity which it had in the veins longer than under other circumstances. In general, at the commencement of a bleeding, the blood is of deeper colour, more dense, and less electrical, than what flows afterwards: in other respects, the blood taken from a vein has a tendency to the equilibrium of the surrounding atmosphere.

A well-marked example of that singular disease to which the name of *anæmia* has been given, has recently been recorded by Dr. COMBE,† who gives the following well-drawn picture of the complaint:

"It was in the month of July, 1821, that I was first consulted by Alexander Haynes, the subject of this case, on the nature of his complaints. Even at that time I was much struck by his peculiar appearance. He exactly resembled a person just recovering from an attack of syncope: his face, lips, and the whole extent of the surface, were of a deadly pale colour; the albuginea of the eye bluish; his motions and speech were languid; he complained much of weakness; his respiration, free when at rest, became hurried on the slightest exertion; pulse 80, and feeble; tongue covered with a dry fur; the inner part of the lips and fauces were nearly as colourless as the surface. He says that his bowels are very irregular, generally lax, and that his stools are very dark and fetid; urine reported to be copious and pale; appetite impaired; of late, his stomach has rejected almost every sort of food; has constant thirst; he has no pain referable to any part, and a minute examination could not detect any structural derangement of any organ. He is forty-seven years of age; was born, and has spent the greater part of his life, in the country, engaged in agricultural employments; for a few years has been servant to a corn-merchant, where his duties are neither

---

* *Memorie della Reale Accad. delle Scienze di Torino*, t. 24.

† *History of a Case of Anæmia.* By J. S. COMBE, M.D. Fellow of the Royal College of Surgeons, Edinburgh.—(Transactions of the Medico-Chirurg. Society of Edinburgh.)

laborious nor unhealthy. He is married, and has no family; leads a regular and temperate life; has enjoyed perfect health since childhood, and has never been blooded. He was advised to use some medicine to correct the state of his bowels, to confine himself to a light diet, and to take gentle exercise."

Tonics (particularly iron), mercury, opium, and other remedies, were employed, and he appeared for a short time to improve; but soon after he relapsed, and, gradually sinking, died in January 1822. On post mortem examination, effusions were found in the chest and abdomen, and a rough irregular ossification, an inch in length, imbedded in the folds of the dura mater, near the vertex; but by far the most important appearance was an almost bloodless condition of every viscus and structure in the body, with the exception of the spleen, which was very soft, and the contents of which "turned out as from a sac," on pressure being applied. The heart, when cut into, was pale, and did not stain linen when rubbed upon it; no blood exuded from the spleen, on cutting into it; the kidneys were nearly bloodless; the arteries universally empty, as were the jugular, humoral, and femoral veins; the whole muscular structure, in every part of the body, exhibited the same pale colour as the heart, which seemed as if it had been macerated for many days in water.

A very lengthy paper has been published by M. GASPARD, on the subject of *putrid diseases*,* in which he attacks BROUSSAIS by a side wind, and earnestly invites practitioners to return to the old treatment of fevers and putrid diseases, by means of antiseptics, acids, astringents, bitters, aromatics, and cinchona. The general manner of reasoning, or rather of discoursing, upon the subject, is this:—he injects water, in which putrid animal matter has been allowed to steep for a longer or shorter period, into the veins of animals; and he finds vomiting, purging, fever, &c. produced; in which symptoms he finds an exact resemblance to typhus, dysentery, and similar complaints. The next step is to immerse animal substances in different fluids, as vinegar, wine, muriatic acid, &c.; and the result is, that they are preserved "almost indefinitely." In the last place, the first experiment is repeated; and, after the animal has had putrid matter poured into the veins, some of the correctives are injected after it, and lo! some of the patients are thus restored. We shall give the first example in the Memoir of each of these experiments; which, we think, will serve to justify the remarks made in a preceding part of this paper.

*Exp.* I.—"The 27th of March, 1823, I injected into the right jugular of a lamb, aged two months, half an ounce of water, fetid, but still transparent, in which some veal had macerated and rotted during some days. The first symptoms which resulted were pain and frequent acts of deglutition during the injection; vain efforts to vomit; and, in a quarter of an hour, loss of strength, inability to stand, respiration somewhat embarrassed, repeated alvine evacuations, abundant flow of mucus from the nostrils, (which continued until death); next, fever,

* *Second Memoire, Physiologique et Medical, sur les Maladies Putrides.* Par B. GASPARD, D.M. &c.—(Journal de Physiologie, t. iv. No. 1.)

small and very frequent pulse, increasing salivation, alvine evacuations, liquid and watery, repeated every instant, but without fetor, accompanied with tenesmus and painful retraction of the abdomen; *at length, long agony, with continual moans and occasional howls, and death seven hours after the injection.*" Of the examination after death, it is sufficient to say, that the stomachs were sound, but the small intestines were inflamed; the smallest laceration or incision into their parietes giving vent to black fluid blood; the lungs spotted black in different points, and showing serous effusion, both into their texture and the cavity of the pleura; the left side of the heart, the liver, spleen, &c. having numerous spots of ecchymosis.

*Exp.* III.—"I preserved for a very long time, and almost indefinitely, some meat in whey, weak vinegar, cream of tartar, wine, very dilute mineral acids, carbonic acid, chlorine, cinchona, gentian, sulphate of quinina, &c."

*Exp.* IX.—"On the 19th of February, I injected into the jugular of a very small dog, at first half an ounce of putrid water, with meat steeped in it, which excited great agitation, with efforts to vomit, followed by a great calm and species of syncope. Afterwards I introduced slowly, and at five times, two ounces and a half of a limpid, but very strong, decoction of red bark. Now the animal, after these different injections, was in a state of stupor, arthenia, and paralysis; which, however, gradually went off a little, so as to allow him to move about, with staggering, for nearly half an hour. At the end of this time, complete failing of strength returned, and he lay upon the side; flaccidity of the members, and soon after very laborious respiration; belly painful and elastic; sero-mucous running from the anus; and then, at the end of an hour, a liquid, yellow, gelatinous stool. After this his condition became pitiable; belly more and more painful, great dyspnœa, redness of the conjunctiva, eyes tearful, stiffness of the neck and limbs of the right side, *continual howling (cris en maniere de hurlements renouvelés a chaque instant).* Two hours after the experiment, I injected again, very gently, and at four times, two ounces of the same tepid decoction of cinchona; but the dog did not appear to experience any other effect or change than the cessation of its cries. As to the rest, it continued in a state of extreme debility, without, however, having any other alvine evacuation; respiration being long performed in sobs, and the animal at length dying three hours after the injection."

Such of our readers as are interested in the subject will find, in the Memoir alluded to, many additional instances of animals similarly tortured.

The opinion that the various forms of varioloid eruptions are but modifications of each other, seems to gain ground; and we find an interesting letter on this subject, from Dr. Stoker, of Dublin, to Dr. Thomson, of Edinburgh,* stating the result of numerous observations in confirmation of this doctrine. He is of opinion that all the pocky

* *Letter to* John Thomson, M.D. *Edinburgh, containing Observations on the Occurrence of Small-Pox after Small-Pox and Vaccination.* By W. Stoker, M.D. Dublin.—(Edinburgh Med. and Surg. Journal, January 1824.)

exanthemata are derived from genuine small-pox, and conjectures "that it is only the peculiar virus of each which can give protection from the various forms which they assume after having once emanated from the parent-stock; forms which characterize them in each future transfer *ad infinitum.*" Dr. Thomson likewise informs us,* that the general results of his later observations and inquiries have tended to confirm the opinions which he formerly expressed with regard to the advantages of vaccination; the frequent occurrence of small-pox a second, or even a third time, in the same individual; and the identity of all the varioloid eruptions. He adds, that he has been led to believe that the *test-pock* of Mr. BRYCE bears the same relation to the primary cow-pock that secondary small-pox does to primary: in short, that cow-pox modify cow-pox, as small-pox modify small-pox.

---

### MEDICINE (*including* THERAPEUTICS *and* MATERIA MEDICA).

A general History of Medicine has recently been published in Germany,† which is favourably spoken of in some of the foreign Journals.‡ It is stated to be taken from the best sources, and to be executed with zeal and talent. The author begins by exposing the state of medicine among the Chinese and Indians, from the earliest times; proceeds to details regarding the science among the Egyptians; and afterwards passes on to the countries of Europe.

We have likewise to notice among historical records an account of the *epidemics* which have at various times prevailed in different parts of the globe.§ The volume is divided into three parts: the first containing the history of epidemics from the most remote antiquity up to the period of general migration; the second, from this time to the crusades; and the third, from the crusades to the invention of printing.

A paper of some interest, connected with the history of medical science, will be found in a recent Number of a respectable American publication.|| One of the circumstances enumerated as retarding the progress of the healing art, or even giving to it a retrograde course, is *the multiplicity of books.* As reviewers, who are compelled to peruse a great proportion of the medical works which issue from the press, we cannot but admit that it is surprising how few of the volumes which pass under our notice are really marked by novelty or genius; how few there are who study nature; how few, *in making a book,* think any thing more is requisite than to borrow from those who have gone before them.

It cannot be expected that we should often meet with new diseases; and the only thing having any claims of this kind is the account of a

---

* *Lib. cit.* p. 92.

† *Geschichte der Heilkunde.* Von I. F. R. HECKER.—Berlin, 1823.

‡ *Leipz. Lit. Zeitung,* and *Bulletin des Sciences Medicales.*

§ *Chronik der Seuchen, &c.* Von T. SCHNURRER.—Tubingen, 1823.

|| *A comparative View of the State of Medical Sciences among the Ancients and Moderns, its Revolutions in different Periods of the World, and an Enumeration of some of the Errors which check its Progress.* By JOHN STEARNS, M.D.—(Philadelphia Journal, February 1824.)

singular affection, attended with hiccough, which occurred to M. MIKISCH, of Horsens.* This practitioner was called upon to attend ten individuals, under the following circumstances. They were attacked with rigors, perspirations, lassitude, and head-ache; bad taste in the mouth, and oppression in the epigastric region; some were constipated in the bowels, while others were affected with diarrhœa. *After a few days, they all had hiccough,* which at first was slight and with long intervals, but afterwards became more frequent and more severe, coming on every hour, and sometimes every half hour. If the patient heard another person hiccough, he began again, even although his own paroxysm might only have just ceased. During these fits, the patients presented a shocking appearance, the respiration being very difficult, and the countenance becoming purple from the interrupted state of the circulation; sometimes the rupture of a vein in the nostrils or fauces gave rise to a considerable loss of blood; and in other instances the patients had epileptic seizures, and rolled on the ground. During the continuance of the disease, none of them were affected with fever, but all were reduced to great weakness; some had a yellow circle round the eyes, as in the commencement of jaundice; the tongue was loaded and brown, and the urine deposited a copious sediment: in some females, the menses were suppressed.

M. Mikisch regarded the cause of this malady as depending upon some circumstance connected with the atmospheric temperature, or with some error of diet. In February much snow had fallen, after which the wind had veered to the south-west, and a complete thaw suddenly followed. The melted snow filled the wells, and was used by the peasants; but no noxious ingredients could be detected in it by chemical analysis. The bread was not bad; and their beer, though not well brewed, was such as the peasantry in Jutland usually employ: their cattle were healthy. Under these circumstances, M. Mikisch directed the patients to be separated, and placed in warm, well-ventilated situations; all spirituous liquors were withdrawn, (they had been using brandy,) and their beer was improved by the addition of bitters, and by attention to the process of brewing. Evacuants were next had recourse to: when their strength admitted, the patients were bled both generally and by leeches; in other cases, cold lotions were applied to the head, and warm fomentations to the limbs; emetics, purgatives, and mercurials, and afterwards bitters and antispasmodics, were administered, as indicated by circumstances.

The disease had broken out in March, and by the middle of May all the ten patients were restored to health. One woman continues to be attacked with hiccough every time her temper is ruffled, or when she remains long without eating; some of the others have a paroxysm after any excess in drinking.

The following case of what may be called *chronic hiccough,* we regard as possessed of some degree of interest, as connected with the preceding narrative. A young man, aged twenty-six, witnessed a scuffle

* *Bibliothek for Lager.*

between two individuals, by which he was much agitated; he had an epileptic fit, and afterwards was attacked with hiccough, which lasted for thirteen months. It came on ten or twelve times every day, and each fit lasted about half an hour: he was free from it during the night. After trying all the usual remedies without avail, recourse was had to sulphuric acid, of which a drachm was added to a pint of water, and three spoonsful taken every three hours. By this treatment he was speedily cured.*

The *croup* has long been known in this country as depending upon inflammation of the mucous membrane of the larynx and trachea, although its pathology would appear to have been less generally admitted in France, if we are to judge from a recent publication, in which much pains is taken to demonstrate the inflammatory nature of the disease.† Although, therefore, this view of the croup cannot be considered as possessing any features of absolute novelty, yet the work deserves to be mentioned in this place, from its containing a more complete history of the disease than any other with which we are acquainted. The author, M. Blaud, divides the croup into three varieties,—viz. that in which a false membrane is found; that in which inflammation ends in suppuration; and that in which the mucous secretions of the part are not altered, but merely increased in quantity. All these three varieties may exist together, and this he designates by the formidable name of *laryngo-tracheite-myxa-pyo-meningogene.* The symptoms are described with accuracy; and, among other circumstances, it is particularly remarked, that the sonorous or croupy cough and respiration do not belong to the variety in which a false membrane is thrown out, more than to the others. The method of treatment recommended by M. Blaud for the most severe form of the disease is remarkable for its activity, and consists chiefly in copious bleeding.

Perhaps we may be expected to take some notice, in this place, of a recent work on Dropsy, by M. Portal.‡ We shall only say, that we read it with the intention of making it the subject of an article in our Foreign Review, but found it so utterly destitute of any claims to novelty, or even to accuracy, that we conceive we best consult the reputation of the veteran author by letting it fall into oblivion as speedily as may be.

Among the very few novelties in the department of Therapeutics, we have to introduce to our readers, is the system of Dr. Hahnemann, under the name of *homœopathia*, which is used to designate the method of treating diseases by means of active medicines in doses of extreme minuteness. The fundamental principle of this theory is, that different complaints are cured by means which produce symptoms analogous to those of the disease: and that the remedy is appropriate in

* *Annali Universali di Medecina,* Novembre 1823.

† *Nouvelles Recherches sur le Laryngo-Tracheite, comme sous le Nom de Croup.* Par P. Blaud, d.m.p.—Paris, 1823.

‡ *Observations sur la Nature et le Traitement de l'Hydropisie.* Par M. Portal, &c. &c.—Paris, 1824.

proportion to the strictness of the analogy between its effects and the disease it is intended to cure. Thus, a chronic disease may be arrested by an acute one, and vomiting stopped by an emetic: in short, the principle is *similia similibus curantur*. Dr. Hahnemann, proceeding upon this idea, argues that, as an atom of vaccine virus is sufficient to give protection against small-pox,—or an atom of any other morbid poison, as syphilis, is capable of giving rise to a disease similar to that in which itself has originated,—so atomic doses of active medicines, provided they be the proper contra-stimulus to the disease, are regarded as preferable to the same medicines in larger quantities. There appears much of hypothetical assumption in these doctrines, so far as we have yet been made acquainted with them; but numerous plausible cases are cited,* and we are promised further details in a system of Materia Medica, by Hahnemann.

We have alluded to the views of M. Hahnemann, however, chiefly for the purpose of introducing one of the most important illustrations of his doctrines,—one which, rejected in France, and almost unknown in this country, is nevertheless regarded by many in Germany as second in importance to the discovery of vaccination alone: we allude to the alledged efficacy of *belladonna as a preventive of scarlatina*. We are told that the author of this system, having observed that the drug in question, when taken in very small doses, produced symptoms analogous to those of scarlatina, was hence led to the conclusion that it was the proper antidote to the disease; a conjecture which at first was regarded, even in Germany, as too improbable to merit serious attention. But, lest our readers should still think thus of Dr. Hahnemann, and his claims to be regarded as a second Jenner, we shall lay before them the opinion of Professor Koreff, in a letter to M. Laennec; an opinion which, he informs us, is founded on the observations of sixteen years.†

"Observation clearly proves," says he, "that the belladonna, taken for some time, either in powder or in extract, produces, especially in infants, a redness of the skin, which is sometimes transient, but at others more durable; dryness of the mouth, with a sense of heat in the throat; dilatation of the pupil; anxiety; and occasionally swelling of the submaxillary glands: symptoms having a great resemblance to those which accompany the eruption of scarlatina. The effect of the belladonna has also this in common with scarlatina, that neither of them produce the redness of the skin invariably, whilst the symptoms about the throat are always present. I confess to you, however, that all these analogies did not appear to me sufficiently strong to persuade me that in this plant was really to be found a preservative against scarlatina, similar to that which the cow-pock affords against variola. It was not till I had received the authority of the celebrated Soemmering, who informed me

* By Dr. Wiedmann, in Hufeland's *Journal der Practisch Heilkunde*, 11 cahier, 1823.

† *Sur l'Emploi de la Belladonne contre la Contagion de la Scarlatine; Note communiquée a* M. le Prof. Laennec, *par le* Prof. Koreff.—(Bulletin des Sciences Medicales, Avril 1824.)

Curron that he had obtained the most satisfactory results with it when the disease raged epidemically, that I determined to employ it. This malady, accompanied by the most unfavourable symptoms, and having entirely changed its usual character, was at that time producing ravages almost as fatal as the contagious typhus. I then, for the first time, had the happiness to protect from this dreadful contagion almost all those who took the belladonna with a little perseverance, and of these there were many thousands. Since that time I have never lost sight of this discovery, which becomes the more valuable as the scarlatina has increased during the last thirty years, both in violence and extent, in many countries; and I have always found the same effects in different climates, and in epidemics of opposite characters. Many other physicians have equally confirmed the preventive powers of this plant, and the German Journals are daily filled with proofs of a benefit which, with respect to some countries, equals that of vaccination. In France, the capital and the provinces of which appear less subject to these fatal epidemics than Germany, Swisserland, the Tyrol, Poland, and the north in general, less attention has been given to this discovery, and it has been rejected,—it must be said too lightly, and without any sufficient examination, as may be seen in the article *Belladonna* in the Dictionnaire des Sciences Medicales. I only remember a single observation on this important subject, by Dr. MEGLIN,* who gives an account of a trial which he gave to this preservative during an epidemic of scarlatina at Colmar, and which confirms all the assertions of the German physicians. The absence of present danger is, perhaps, the cause of this indifference towards a discovery, which, important in itself, might also be fruitful in results applicable to other diseases. At present, however, I shall confine myself to an account of the results which have been ascertained, (by repeated observations, and by a great number of individuals placed in very different circumstances,) without incurring the reproach of having proceeded in a manner not sufficiently rigorous.

"The powder mixed with sugar, or the extract made very carefully from the juice of the recent plant, are employed after the following formulæ:—Extract of belladonna, three grains, dissolved in an ounce of cinnamon water. Powder, or root of belladonna, two grains, mixed with ten drachms of white sugar, divided into sixty doses. From half a dose to a whole one is given to a child, from six months to two years old, four times a-day; to children from three to six years old, from a dose to one and a half; to those from six to nine, two to two and a half; to those from ten to twelve, three, to four and a half. Of the solution, a drop is given for every year of the child's age, once a-day and fasting. Observation has shown that, when the epidemic is very fatal, or the intercourse with the patients very frequent and intimate, it is prudent to increase the dose a little. It has not yet been possible to determine, in a satisfactory manner, the length of time which is necessary to eradicate by this remedy, the susceptibility of the contagion. Every thing leads

* *Nouveau Journal de Medecine*, 1821.

us to believe that the remedy, if used during a time too short to ward off the contagion, moderates very much the malignity of the disease. We know for certain that the remedy does not permanently overcome the disposition to scarlatina; and it is necessary to resume its use on every recurrence of an epidemic. We have always observed that the most intimate communication with the sick does not produce the disease, provided the medicine has been employed eight or nine times previous to being exposed to the contagion, and continued up to the period of desquamation; a circumstance very important to nurses. It appears more certain to begin with rather strong doses, in order to guard against the first impression of the contagion, and to diminish the quantity after a few days. No sensible effect has been observed to follow the continued use of this small quantity of belladonna. Up to the present time, neither season nor locality, nor any other circumstance, has appeared to diminish the preservative effect of this plant."

After remarking that the belladonna does not exercise the same power over miliary scarlatina, he proceeds:—"Do not believe, my learned colleague, that these results have been too lightly deduced, or from a small number of individuals, or from epidemics of little violence. It is from entire provinces,—from cities affected with this terrible scourge,—from epidemics the most fatal, in all seasons, and in localities the most diversified,—on individuals of every age and of every condition, that observations have been made with the greatest accuracy, and have led to the above results.

*Belladonna* has been long used by CHAUSSIER in cases of rigidity of the os uteri, in the following manner:—Rather more than two drachms (*huit grammes*) of the prepared extract are softened with an equal quantity of distilled water, and incorporated by trituration with an ounce of hog's-lard or simple cerate. In order to secure all the benefit which this means is capable of affording, it is necessary that this preparation be applied directly to the orifice of the uterus. To effect this, he has contrived a small syringe, which, in place of having a pipe, is rounded at its extremity, and has an opening large enough to admit the point of the little finger. The piston is pulled back a little, then about the size of a small nut of the ointment is introduced at the upper orifice; the syringe is directed by the finger to the mouth of the uterus, the piston is pushed home, and the ointment forced out; being of a soft consistence, it liquifies and spreads over the parts. At the end of thirty, or at most forty, minutes, the os uteri becomes soft and relaxed, to such an extent as no longer to present any resistance to the efforts to dilate it, or to the contractions of the uterus itself. This method was extensively employed by the late Madame LACHAPELLE, and is likewise commended by M. LEGRAND, the present midwife in chief at the Maison d'Accouchement.*

---

* *Journal Universal des Sciences Medicales*. 96 cahier.

† *Traité de la Methode Fumigatoire, ou de l'Emploi Medical des dains et douches de Vapeurs.* Par M. RAPOU, D.M.P.—Paris, 1824.

A very elaborate work has been published by Dr. RAPOU, on the therapeutical applications of *baths and vapours*,† in which it is almost unnecessary to say that the "*methode fumigatoire*" is preferred to every other. There are, however, many interesting remarks scattered in the work, and many useful hints with regard to the effects of different medicinal substances, when thus employed in the form of vapour. It would, *a priori*, be natural to suppose that the body would be rendered particularly susceptible of cold by the vapour-bath, but experience has proved, on the contrary, "that when the movement of reaction from the centre towards the circumference is powerfully established, we may be exposed to a very severe degree of cold, without experiencing any disagreeable impression or the slightest inconvenience." Thus it is that the Russians are in the habit of rolling themselves in the snow, the moment they leave an atmosphere heated to a high temperature. The determination to the skin, particularly if the bath has been accompanied by frictions, continues for several hours, and gradually diminishes; and Dr. Rapou, who has tried the experiment on his own person very frequently, says that, on quitting the vapour-bath in winter, the cold air always afforded him a degree of pleasure, similar to that which results from a cool breeze in a hot day; and that he "has always been obliged to put on less warm clothing during several days." The general effects resulting from the action of heated vapour on the human body, are more minutely and satisfactorily explained than in any other work with which we are acquainted; and the following may be taken as some of the most important conclusions.

"1. That, in certain nervous temperaments, the vapours of camomile and mint evidently act as antispasmodics. 2. That vapour impregnated with mugwort, wormwood, and rue, applied as a bath to the middle of the body, has always recalled the menses, after all other therapeutic agents had failed. 3. That the vapour of roses has a directly sedative effect, and is of great use in certain inflammatory irritations of the skin. 4. That the vapour of elder-flowers possesses the same properties in a much higher degree, and is one of the most powerful means that can be applied to counteract acute pain, or the intolerable pruritus of many cutaneous eruptions. 5. That vapours impregnated with the narcotic principle of poppy-heads, the leaves of the nightshade; dry fumigations with extract of henbane, and more particularly with opium, speedily produce a sedative effect, in cases where other means have been employed without avail; but that, to insure this effect, the vapour must be employed at a mild temperature. Animal substances seem to have been but little tried by Dr. Rapou, but he has borrowed assistance very largely from the mineral kingdom: among these, we find sulphuretted hydrogen spoken of in terms of high commendation, particularly in cutaneous affections: "directed at a gentle temperature on an inflamed part, it speedily diminishes the pain, redness, and swelling, manifestly moderating the capillary circulation of the skin. Muscular or nervous pains, which obstinately resist other means, yield to its influence, especially if connected with inflammatory irritation: thus, acute, herpetic, and similar eruptions, derive the most essential benefit from it."

We observe that the application of *iodine* in the form of vapour has been suggested, for which, indeed, its volatility would appear to render it particularly proper; but we are not aware of any satisfactory experiments upon this part of the subject.

Some useful practical remarks on *the treatment of obstruction of the bowels*, have been made by Dr. MAXWELL, of Dumfries.* They relate chiefly to the effect of *injections*, variously modified, in administering which, he is of opinion that the patient ought to be placed in such a position as shall allow the bowels to hang " nearly at right angles with the spine." Dr. M. informs us that, by attending to this circumstance, he threw up three and a half gallons of warm water, accurately measured. He regards the general idea, that the valvular structure between the colon and ilium can prevent the passage of enemata, properly administered, is incorrect; and believes, on the contrary, that, in a healthy state of the intestines, any quantity of water may be made to pass from the anus to the mouth. He has found, however, that injections of water are sometimes insufficient to remove the obstruction; in which case, he has recourse to inflating the bowels with air. The first instance in which he tried this experiment is thus described:

" A piece of cork, nearly flat, was placed on a male catheter, at three inches from the point, which was introduced into the rectum. The patient being placed on his back in bed, and the cork pressed firmly against the anus, the bowels were gradually inflated, the top of the catheter being stopped with the tongue during inspiration. When the air had occasioned considerable distention, it was readily expelled, but nothing followed. Pondering upon my disappointment, it occurred to me that the sudden distention of the colon might press so much upon the ilium, that the air could not enter it. I resumed the operation, blowing slowly, and with the left hand pressing the air forward along the colon into the ilium. When the distention began to give much pain, the air was allowed to escape, and in about an hour it was followed by copious soft stools."

Dr. Maxwell mentions ten other cases in which he employed this plan with success; to insure which, he recommends that the patient should lie on the back, the distention be made gradually, and the air be pressed along the colon into the ilium. In two instances where, from the supervention of symptoms of palsy, it seemed probable that the constipation arose from a paralyzed state of some portion of the alimentary tube, and where the bowels remained insensible to the operation of the usual stimulus of cathartics and enema, Dr. Maxwell employed electricity, by passing brisk and repeated shocks through the abdomen: in both cases the bowels acted, and in one the patient ultimately recovered.

Various cases have recently been published, in which the *oil of turpentine* has been administered with advantage in *purpura hæmorrhagica.*

---

* *Observations on Constipation, with Cases.* By W. MAXWELL, M.D.—(Edinb. Med. and Surg. Journal, Jan. 1824.)

They rest on the respectable testimonies of Mr. THOMPSON, of Whitehaven,* and Dr. WHITLOCK NICHOLL.†

Some examples of the utility of the same drug in *puerperal fever* have been lately published by Dr. JOHNSON, of Chatterton.‡

The oil of turpentine has likewise been strenuously recommended in the treatment of *neuralgia* of the sciatic and other nerves of the extremities, in a Memoir by MARTINET, written expressly on the subject.§ It is maintained that the virtues of the drug reside in the volatile oil, and not in the resin which it contains; that they are unconnected with any purgative, diuretic, or diaphoretic effect; but that it acts directly upon the nerve, exciting a sensation of heat along its course. It is recommended to be given in doses of a scruple three times a-day, in some aromatic syrup,—a larger dose is useless, if not hurtful; severity of pain and well-marked exacerbations are the most favourable circumstances for its exhibition; and six or eight days are generally sufficient to effect a cure. Of thirty-six cases recorded by Dr. Martinet, twenty-six were completely cured, seven were relieved, and three only derived no benefit from the turpentine: they were affected with disease of the hip-joint, which proved fatal.

The same physician has likewise published some remarks upon the doses of *sulphate of quinina*, which he recommends to be administered from the commencement in quantities of twenty, thirty, or thirty-five grains.

A case is related by Dr. BEATTY, in which a child of four years old, affected with *ascites*, was cured by the *pyrola umbellata*, after other diuretics had failed.||

"It was, however, proposed, as a last resource, previous to resorting to the operation, that we should try the pyrola umbellata, described by Dr. Somerville in the fifth volume of the Medico-Chirurgical Transactions. I directed an ounce of the plant to be infused in a pint of boiling water for an hour, and half a pint of the liquor strained off, to be divided into six draughts, one to be taken three times daily. The result was such as to exceed the most sanguine expectations: the urine, almost immediately, became natural in appearance, and was increased to three or four times its usual quantity; the alvine discharge, for the first time since her illness, recovered its natural colour and consistence. Her general appearance was sensibly improved, and her spirits began to return."

The volume above referred to contains several examples of *hæmatemesis* successfully treated with *ipecacuanha*, by Dr. SHERIDAN, who says—"These cases, with the two others which I observed in the country, have made such an impression on me, that I never can hereafter

---

* *London Medical Repository*, November 1823. † *Ib.* June 1824.

‡ *Philadelphia Journal*, February 1824.

§ *Memoire sur l'Emploi de l'Huile de Terebenthine dans la Sciatique et quelques autres Neuralgies des Membres.* Par L. MARTINET, D.M.P.—Paris, 1823.)

|| *Transactions of the Association of Fellows and Licentiates of the King's and Queen's College of Physicians in Ireland.* Vol. IV.—Dublin, 1824.

hesitate for a moment to have recourse to the ipecacuan emetic in hæmatemesis. I do not mean by this term a mere vomiting of blood, which may be produced by various causes, and in some cases of which emetics might prove very prejudicial, but the true hæmatemesis, which I think will always be sufficiently distinguished by the symptoms which have been above described."

In our last Historical Sketch, we laid before our readers some interesting observations made by Dr. HELLER, on the use of *hydrocyanic acid* in *diseases of the heart;* we have now to mention a useful caution with regard to inhaling the vapour which fills the upper part of a phial containing a portion of this very powerful narcotic.* A distinguished chemist in Paris, in arranging his laboratory, perceived that a phial, half filled with the hydrocyanic acid of Scheele, contained some dark-coloured flakes, and, conceiving that it had become decomposed, he was about to throw it away. Desirous, however, of ascertaining its exact position, he took out the cork, and applied the phial to his nose. At this instant he was seized with a great tightness in the chest, and felt three different times successive shocks directed towards the heart, (*secousses precipitées vers le cœur;*) he became pale with general weakness, and a sensation of pricking all over the skin, which ended in stiffness all over the body. This last symptom was particularly remarkable in the legs, which he constantly endeavoured to bend; but he performed these movements so incompletely, that the limbs resumed their former position as often as he attempted to overcome the force which kept them extended. The vapours of ether and ammonia were applied to his nostrils; he was carried into the open air, and every effort made to recall his sensibility. At this time, Dr. Heller having arrived, he found the pulse extremely contracted, and so slow that it only beat thirty-six in the minute. The patient, however, had not lost his perception: on the contrary, he was so much agitated as to render it difficult to compose him. The skin was rubbed with cloths dipped in ether and hartshorn, and he took half a cup of very strong coffee every half-hour. The circulation, notwithstanding, continued oppressed, and the pulse not rising above forty. Under these circumstances, three spoonsful of oil of turpentine were given in the coffee, and repeated twice within two hours. The distress of the patient, however, lasted during the whole of the ensuing night; but next day general lassitude and depression were all that remained. Unfortunately, however, inflammation of the stomach and bowels followed; and he ultimately recovered with great difficulty, and after long and rigorous treatment. The latter part of the evil, however, is frankly attributed by Dr. Heller to the strong coffee and the turpentine, which were so liberally administered, and the impropriety of which he fully acknowledges.

It is rather singular that this accident should have made so little impression on the narrator as it appears to have done; for he goes on to

---

* *De la Necessite de ne point trop insister sur l'Usage interieur des Excitans dans l'Impoisonnement par l'Acide Hydroganique.* Par M. HELLER, &c.—(Journal Generale, Mars 1824.)

inform us that he went, in the course of last summer, to the Veterinary School at Alfort, where he made numerous experiments with prussic acid on animals affected with hydrophobia; and that, having for some time held in his hand a phial half filled with acid, which had been prepared by Robiquet two days before, he took out the cork, and held the phial under his nostrils. Symptoms followed resembling those above mentioned, consisting in a sense of pricking along the mucous membrane of the nostrils, fauces, and larynx; giddiness, weakness, trembling, and general sense of cold. He was made to inspire ammoniacal vapours, by which he was a little restored, and went into the open air, but retained much weakness and oppression during the rest of the day.

Dr. Heller states, that during two years that he has administered prussic acid to numerous patients, of all ages, of both sexes, and different temperaments, he has sometimes seen debility and slowness of the pulse follow its use in too large doses; and occasionally even coldness of the extremities, and a general numbness or stiffness; but that he has always found frictions with warm flannels steeped in ammonia, exposure to fresh air, and making the patients move about, sufficient to relieve the unpleasant symptoms.

The interesting part of Dr. Heller's paper consists in the important caution to chemists, not to inhale too freely the vapours which collect in the upper portion of a phial which is partly filled with prussic acid. The assertions he makes with regard to the treatment adopted in France and England,—viz. that oil of turpentine is generally given as an antidote, is entirely without foundation. In this country, at least, when an over-dose of prussic acid has been taken, practitioners are accustomed to have recourse to brandy and ammonia, as recommended by Dr. Granville and Dr. Murray.

The *pulvis antimonialis* of the London Pharmacopœia was made the subject of experiment by Dr. Elliotson some time ago, with a view of ascertaining its real effects and proper doses. We are inclined to think his observations have not met with the notice they deserve,—at least, they have not had any influence with those to whom we are indebted for the New Pharmacopœia. Dr. F. Hawkins, apparently ignorant of Dr. E.'s remarks upon this subject, has published a paper* in which he has arrived at similar conclusions: indeed, he asserts that the effects attributed to this medicine are not to be produced by ten times the usual dose; and he informs us, that he now begins by exhibiting half a drachm at once. One case is mentioned, in which two scruples were given twice a-day for several weeks: "no function was in any degree affected by it." In another instance, so much as one drachm was exhibited night and morning, without any sensible effect. It is proper to remark, that Dr. H. procured some pulvis antimonialis from Apothecary's Hall for the purpose of experiment.

---

* *On the Dose of the Pulvis Antimonialis.* By F. Hawkins, M.D. &c.—(Edinb. Med. and Surg. Journal, April 1824.)

### SURGERY.

Operative surgery has now nearly achieved all that can be expected from it. The ligature of the aorta, and the internal and external iliacs, —of the subclavian and carotid arteries,—the excision of the jaw,—amputation at the hip-joint,—such are the triumphs of modern surgery; and most of them have now been performed more than once or twice, and some of them ceased to be even objects of much curiosity. We may now, therefore, have leisure to pause over the accounts of these splendid achievements; the wonder and admiration which they have excited may be presumed to have in some measure subsided, and we may be allowed to inquire whether some of these operations may not be undertaken occasionally upon light grounds; and whether we ought not rather to lament the imperfect condition of a science, that renders such mutilation, and such formidable measures, necessary for the preservation of the patient's life. The ligature of a large arterial trunk implies that we cannot cure an aneurism,—that we neither know how to detect its nascent symptoms, or how to put a termination to its progress. Excision of the lower jaw teaches us that we know nothing of the nature of cancer, and of that class of diseases which are nearly allied to it. We know neither the kind nor condition of constitution most prone to the attacks of this formidable disease; and still less are we acquainted with any remedies that control its advances. Amputation at the hip suggests to our minds many melancholy reflections; for it reminds us of many formidable diseases which we are thereby anxious to get rid of, because we cannot cure them.

These suggestions must necessarily arise in the mind of every surgeon who has passed over the first few years of his practical career; and, although humiliating, they are necessary,—nay, they never were more necessary to be encouraged than at this moment; for the splendour of an operation is apt to dazzle the imagination, and overwhelm the judgment, of the young and sanguine, and to turn them from the true object of surgical science—the cure of the disease.

The object we propose by these remarks, is not that of depreciating the value of any of these great triumphs of our art; but it is to repress the ardour that we too often see exhibited by the young surgeon to perform these operations, without due consideration or caution, and to impress upon him the necessity, the humanity, and the superiority, of dispensing with the knife upon as many occasions as possible, and of restricting these severe operations to great and urgent occasions only. We are quite sure that hundreds of arms have been removed from the shoulder-joint inconsiderately and needlessly; and the same mania for the hip-joint operation, we fear, is now likely to produce an equal number of victims to that still more unwarrantable mutilation,—unwarrantable, excepting in some very rare and uncommon instances. On this subject we beg to refer our readers to a late controversial work by Mr. C. Bell, and which we shall have occasion to notice again presently.

We now proceed to describe some operations which have been recorded within the period now under review; and we shall commence by Mr. Syme's case of *amputation at the hip-joint*, which, as far as regards the operation itself, was successful, since the patient lived a month after its performance, and the wound was nearly healed; ascites, the consequence of diseased liver, appearing to be the immediate cause of death. Mr. Syme's patient was a lad, aged seventeen years, who had been afflicted with necrosis of the thigh-bone for upwards of two years, and who had resisted the performance of amputation, which had been recommended by his surgeon, until his constitution had evidently become broken down by the effects of the disease. Having decided upon the removal of the limb, Mr. Syme thus proceeds:

"The next question was—at what part of the limb should amputation be performed?

"In the earlier periods of the disease, nothing forbade removal below the trochanters; but now the case was very different.

"I have said that the swelling could be traced distinctly to within an inch of the trochanter major, consequently it must reach the trochanter minor; and every anatomist knows that, if the bone were cut higher than this latter process, the capsular ligament must be opened. Besides, granting that it was possible, by sawing obliquely through the spongy tissue which constitutes the bone between the trochanters, to remove all the sensibly swelled part without injuring the joint, still, considering the duration and spreading disposition of the disease, it was not probable,—I should rather say it was not possible,—that any portion of the bone could be sound or disposed for healthy action: and, even if this difficulty could be got over, there would still remain another of equal magnitude,—I mean the formation of sufficient flaps.

"Plenty of skin might be got; but it was not likely that the thin, exhausted, and distended muscles of the thigh would show much inclination for union, even if they could be secured in sufficient quantity to cover the bone; and this the most hasty examination was sufficient to prove altogether out of the question. * * *

"On the 2d of September, assisted by my much esteemed and highly respected friend and instructor, Mr. Liston, and in presence of Dr. Abercrombie, Dr. Anderson, of Leith, Dr. Scott, and Mr. Marshall, surgeon to the forces, I performed the operation in the manner following.

"Having, with some difficulty, placed the patient upon a table, so that the affected limb was perfectly free, and ascertained that Mr. Liston was ready to make pressure when and where required, I introduced a narrow knife, about a foot long in the blade, which was sharp at one edge only, at the proper place for transfixing the limb; but being prevented, by the bent position in which, owing to long habit, the patient obstinately retained it, from passing onwards in the direction of the tuberosity of the ischium by the neck of the femur, I lost no time in the repetition of fruitless attempts, but instantly changed my plan.

"Without removing the point of the knife, I brought down its edge obliquely, and, by a sawing motion, quickly cut back, in a semicircular direction, to the tuberosity of the ischium, up along the femur and round the trochanter major, so as to form very speedily identically the same flap which would have resulted from the plan I meant to have followed.

"While Mr. Liston covered the numerous cut arteries with his left hand, and compressed the femoral in the groin by means of his right, I

gathered together all the mass of undivided parts on the inner side of the thigh with my left hand, and then insulated the neck of the bone by passing the knife close past its lower surface. I now cut close down along the bone for some way below the trochanter minor, and lastly made my way outwards obliquely, so as to form a good internal flap.

" Mr. Liston holding aside the flaps, I made a single cut with my long knife upon the head of the bone, which started with a loud report from its socket, as soon as abduction was performed. Finally, I passed the knife round the head of the bone, cut the triangular and remaining portion of capsular ligament, and thus completed the operation, which certainly did not occupy, at the most, more than a minute.

" I then proceeded, without delay, to take up the arteries, which were tied by our very promising pupil, Mr. Thomas Evans.

" As soon as the femoral, which had been completely commanded by pressure in the groin, was secured, Mr. Liston relaxed his hands, in order that we might form some estimate as to the size and number of bleeding vessels; and then, had it not been for thorough seasoning in scenes of dreadful hemorrhage, I certainly should have been startled, prepared as I was to expect unusual vascularity, owing to the extensive action so long carried on in the limb.

" It seemed, indeed, at first sight, as if the vessels which supplied so many large and crossing jets of arterial blood could never all be closed. It may be imagined that we did not spend much time in admiring this alarming spectacle. A single instant was sufficient to convince us that the patient's safety required all our expedition; and, in the course of a few minutes, hemorrhage was effectually restrained by the application of ten or twelve ligatures.

" The flaps were now brought together, and retained in contact by means of five or six stitches. Some dry caddis was laid over the wound; and, lastly, I applied a single-headed roller obliquely round the body and stump, moderately tight, so as to afford proper support to the flaps; and then we lifted into bed the patient, who was wonderfully little exhausted.

" The operation was performed at twelve o'clock; and, during the remainder of the day, nothing occurred of much interest, except occasional vomiting, which I thought, at the time, might be attributed to the effects of an opiate given about two o'clock, on account of pain and restlessness."*

We need not detail minutely the reports of the progress of the wound towards recovery. About a month after the operation, the wound was nearly healed, all the ligatures having been withdrawn the week previously; when, as we before said, death ensued from ascites.

The case is closed by some remarks from the author upon the objections usually urged against this operation, and which, we readily agree with him, cannot hold good when a decided necessity for its performance is made out. In the above instance, Mr. Syme is of opinion that the common mode of amputation could not have been adopted with any chance of success. We are very unwilling to put our opinion against

* *Edinburgh Med. and Surg. Journal*, Jan. 1824.

that of a gentleman, who not only is eminent in his profession, but has the advantage of having examined the patient, which we of course did not, and whose justification (the diseased bone itself) is in his possession; and which, having been seen by his professional friends, (neither few in number, nor little entitled to respect,) have all agreed as to the necessity there was for the entire removal of the bone; still we trust we shall not be accused of over-caution, if we say that a more lengthy and accurate description of the condition of the bone would have been more satisfactory to our minds, since the circumstance of its being *thickened* higher up than the trochanter minor is at best but equivocal.—We shall have occasion to revert to some observations of Mr. Syme's upon amputation generally, in a future part of this paper.

*Amputation at the hip-joint* has also been performed lately by Sir ASTLEY COOPER, with a successful result; but we have no particulars of that operation upon which we can rely, and therefore we shall wait the publication of the case itself. We understand that there were only four vessels secured, and that the operation occupied altogether thirty-five minutes.

The next great operation we have to record is *excision of nearly the whole of the lower jaw,* by M. LALLEMAND;* and, terrific as the detail of the case is, it presents many points of interest to the surgeon; and we cannot too much praise the presence of mind, and the fertility of expedient, which the operator exhibited under circumstances so alarming. The subject of this operation was a labourer, named Louis Guillot, aged forty-seven, of a robust constitution, born of healthy parents, who had, in the year 1820, three or four red pimples on the lower lip, containing some purulent matter, which was discharged whenever he scratched them; greenish scabs succeeded to these pimples, which, falling off, left ulcerated surfaces, which finally spread and swelled up, and formed a large fungous mass. After two years and a half of treatment which proved unavailing, he came to the Hospital of St. Eloi, on the 9th October, 1823. The whole lower lip, the chin, and about one inch of the cheek and upper lip of the left side were transformed into a kind of cauliflower growth, the excoriated surfaces of which bled readily, and were covered with an ichorous matter, of a very fetid odour. The neighbouring parts of the cheek were indurated and thickened for an extent of five or six lines round this fungous growth. The slightest touch produced exquisite pain, which sometimes came on without any apparent cause. One of the submaxillary glands was enlarged to the size of a pigeon's egg. The periosteum, felt behind the alveolar border, was thickened and hard. It was evident that nothing but an operation could relieve this man, who readily consented to it; and, on the 11th of October, it was performed in the following manner:

The diseased parts were included within two curved incisions, beginning on the upper lip, six lines from the commissure of the right side to one inch on the left side, and terminating towards the middle of the

* *Journal Complementaire,* Mars.

thyroid cartilage. Very convex above, they encroached upon the sides of the cheek, just anterior to the masseter muscle; below, they were nearly straight,—thus representing, pretty exactly, the shape of a heart upon cards. The arteries, as they were divided, were compressed by the fingers of assistants. On the left side, the tumor extended the whole length of the periosteum to the masseter; a portion of the insertion of which muscle was detached, and the bone was sawed off to within an inch of the angle opposite the interval between the two molar teeth; but the bone could not be entirely separated, on account of the root of one of the teeth, which was obliged to be divided by the chisel and mallet. On the right side, M. Lallemand expected that the saw would meet with no impediment, as there was only one of the molar teeth remaining; but, the stumps of the other being still in the alveoli, obliged him again to have recourse to the chisel. The body of the jaw being then reversed forwards, the soft parts were divided; the portion of the skin of the neck included between the two incisions was then divided; and, just as this was complete, the patient appeared to make great efforts to respire, immediately afterwards sinking from his chair, his head thrown back, and fell on the ground, in spite of the assistants, in a state of insensibility. But, although he still continued insensible when removed to his bed, the blood was pouring forth with violence from twenty vessels; and, therefore, M. Lallemand justly concluded that this was not a syncope. The efforts the patient made to respire induced the operator to think that the pharynx might be loaded with clotted blood; but, on inserting the finger into the mouth, it appeared that the tongue was forcibly drawn to the back of the mouth, and the posterior face of the larynx rested against the vertebral column. The section of the genio-glossus, genio-hyoideus, and mylo-hyoideus muscles, their antagonists being undivided, had caused this inversion of the respective situations of the tongue, the larynx, and trachea.

M. Lallemand draws a very vivid picture of the alarming situation in which he was placed. Fortunately he had cauteries heated, and he applied them over the bleeding surface, so as to restrain the hemorrhage. He then extended the incision downwards; laid bare the trachea below the cricoid cartilage, and opened it. In an instant the air rushed into the lungs, the chest became dilated, and the man awoke as if from a profound sleep. Want of space obliges us to be concise in our detail. The wound was dressed with charpie, and drawn together with straps of sticking-plaster. Suppuration was established on the fourth day; on the seventh, the eschars formed by the cautery were separated, leaving a healthy surface beneath; and finally, on the 4th of December, 1823, Guillot was completely cured; there remaining only, of this great loss of substance, a space of two inches between the two extremities of the jaw.

This case, which we have been obliged to abridge, extends through several pages, and, though highly creditable to M. Lallemand, we think it hardly offers any encouragement to repeat so formidable an operation.

In the Archives Generales (January 1824,) we have an account of *the extirpation of the parotid gland*, by M. Beclard: the disease was a cancerous ulceration of the gland. "The disease had commenced eight years previously; but, though indolent for a long time at first, it had latterly increased rapidly, and become the scene of lancinating pains. It was fixed; and, when he entered the hospital, it was of considerable elevation. It raised the lobe of the ear above, apparently involving the cartilaginous portion of the auditory canal; downwards it extended more than an inch below the angle of the jaw; backwards, it adhered to the sterno-mastoid muscle; and anteriorly, it covered a great part of the masseter. It was ulcerated in two places.

"*Operation.*—The tumor was inclosed by two curved incisions, one inferior and one posterior. That part situated over the masseter was easily dissected off. Then an attempt was made from below upwards; but a projection of its substance plunged deeply behind and beneath the internal pterigoid, the removal of which would, the operator thought, endanger hemorrhage. M. B. therefore decided to dissect upwards, by striking the bistoury into the structure of the tumor itself, on a level with the projection, while the instrument divided the cellular tissue connecting it with the adjoining parts. Half the inferior circumference of the cartilage contributing to form the auditory canal, was removed by the first dissection. Numerous arteries were tied at this stage of the operation; and M. Beclard continued the extirpation of the remainder of the tumor. When nearly the whole scirrhous mass was removed by successive slices, a large jet of arterial blood announced the section of the external carotid, or one of its large branches. A finger was put on the place whence the jet issued, and the vessel was seized with the forceps, while a needle with a double ligature was passed around it. An assistant tied the vessel above and below the wound in it, which was lateral. The artery was then held forward out of the way, whilst the surgeon completed the extirpation of great part of the tumor. One small projection of the tumor, placed before the cervical vertebra, was left, on account of its proximity to the internal jugular vein. M. Beclard passed two ligatures beneath this part, by means of a needle, tying the one at the superior, the other at the inferior extremity. The wound, which formed a tremendous chasm, was dressed forthwith. Nothing particular occurred for the first days after the operation. All that side of the face was bereaved of expression. The right eye remained open, and, in consequence of being dry, became inflamed. The suppuration was going on kindly, and healthy granulations covering the wound, when, on the twelfth day, the patient experienced rigors, followed by fever. Erysipelatous inflammation attacked the neighbouring parts, and delirium supervened. When this subsided, taciturnity ensued, and ultimately mental alienation, ending in death, better than three months after the operation; the wound being closed except in one place near the ear, where it was again assuming the cancerous appearance.

"On dissection, the pia mater was found injected, water in the ventricles, some pus in the meatus auditorius. The external carotid artery was found to terminate in cellular membrane, resulting from cicatrization of the wound; the internal jugular vein was obliterated at the same place."*

* *Medico-Chirurgical Review,* June.

In this operation we cannot find any thing to commend: considering the extent of the disease, its attachments, &c. we do not think that any prudent surgeon would have ventured upon its removal, because it was (unlike the case just recorded) nearly impossible to suppose that the whole of the diseased mass could be extirpated. This proved to be the case; and, after three months of suffering, the man died, the wound being closed, *except in one place near the ear, where it was again assuming the cancerous appearance.*

M. Maréschal has given us an account of *the extirpation of a fungous tumor of the upper lip*, by M. Roux, in which the ligature of the external maxillary, the right suborbital, and the left coronary arteries, was first performed. The detail of this case is most tediously minute, and is preceded by some reflections upon varicose or aneurismatic tumors, which however present no point of novelty. The patient, a young female, seventeen years of age, was admitted into La Charité, in November 1823, with a considerable tumor, which had existed eighteen months, on the right side of the upper lip. It was not preceded by any congenital mark upon the skin, but showed itself suddenly, and did not attract the particular notice of the patient until it acquired some size. At the date above mentioned, the whole right side of the upper lip and the corresponding cheek, formed a soft tumor, of a violet colour. The edge of this tumor was not well defined on the side of the cheek, so that it was difficult to decide where the alteration of structure terminated. It presented pulsations evident to the eye, and still more so when included between the fingers and thumb. Besides this pulsation of the whole mass, several isolated pulsations, indicating the course of arterial branches over the part, were visible. Compression upon any of the principal arteries of the face produced no change in these appearances; but, if all of them were simultaneously compressed, the tumor was sensibly lessened. Upon this was founded M. Roux's determination of tying the arteries we have mentioned above; after which he proposed to employ pressure upon the tumor, by means of two metallic plates adapted to the shape and size of the tumor, and which were acted upon by a screw, so contived as to diminish or increase the pressure at pleasure. On the 30th of November the arteries were tied, and the tumor lost all its pulsation, excepting a slight one on the posterior surface of the lip, near its edge. The machine for compression was then applied; and on the sixth day the apparatus was removed, when the lip, the tumor, and the neighbouring parts of the cheek, were found in a state of high inflammation; but which, after some days, subsided, and then it was perceived how much had been gained by the ligatures and the compression, both with regard to its extent, form, and *maniere d'etre;* and, therefore, M. Roux proceeded to complete the cure by extirpation, which was performed on the 15th of January, and the patient was in a state to leave the hospital on the 31st.

We are inclined to think that this case might have been better managed. It is evident that the cases of Mr. Travers and Mr. Dalrymple gave the hint to M. Roux for the ligature of the arterial branches; but why was the metallic compression employed? It evidently did no good, and what could it be expected to have done? It brought on pain, inflammation, and very nearly suppuration; and then it was taken away. We think that, in this country, bolder measures would have been pursued than the mere tying the three arteries: at any rate, there could have been no reason to have prevented the excision of the tumor as soon as the supply of blood had been cut off by their obliteration.

Operations for the cure of *popliteal aneurism*, by tying the femoral artery, are now so common as not to merit particular detail; and therefore we need do no more than observe, that Mr. EASTNER has published an account of a successful case of *the ligature of the crural artery*, in the Transactions of the Medical Society of Copenhagen.

Our continental neighbours afford us another remarkable instance of their bold and successful application of surgery, in *the extirpation of an immense tumor*, composed of the diseased integuments of the penis and scrotum. The following account will be read with interest, and perhaps with astonishment.

"A baker of Perpignan had some preputial excoriations, which were considered to be chancres, but they did not yield to mercury; and he enlisted in the gendarmerie on the confines of Spain. Riding produced engorgement of the prepuce, which at length ensheathed the whole penis and scrotum. In six years the tumor had advanced till it hung down below his knee.

"The patient was admitted into the chirurgico-clinical department of Montpellier, where Professor DELPECH recognized in the tumor the characters of elephantiasis; and, having determined that the testicles and the penis were sound, and that there were no other symptoms of the same affection, he conceived the following plan of operation:—1. To make two semicircular flaps with the sound skin on the two sides of the neck of the tumor, each one commencing at the corresponding inguinal ring, and ending before the anus. 2. In the interval of these two flaps, to form a third with the sound skin of the anterior portion of the neck of the tumor. 3. To uncover, right and left, the testicular cords; to ascertain if there was any hernia; to follow, then, the course of the cords in the tumor down to the testicles; to isolate these last, with their cords, and to place them upon the abdomen. 4. To slit open, from below up, the swollen sheath as far as the glans; then to dissect and denude the penis, and to place it on the abdomen. 5. To take away all the tumor in dissecting the perineum. The operation was accomplished, and lasted fifty minutes, and the tumor weighed sixty pounds. The testicles were enveloped in the two lateral flaps, which went to form a new scrotum. The penis was inclosed in the middle flap, which formed a new sheath. In six weeks after, every thing was cicatrized. The only inconveniences which followed the operation were a deep-seated spasm, relieved by two doses of opium the same day of the operation, and a slight fever the five following days. A small part of the anterior flap perished by gangrene, so that the penis has no prepuce. The scrotum is well formed, and the testicles move freely within it. Professor Delpech was under the necessity of digging down eight or ten inches through the immense mass of fat which formed the tumor, before he discovered any vestige either of cord or testicle. This was the occasion of considerable detention in the progress of the operation."*

Amongst the record of operations, we must not forget to mention Mr. SLEIGH's improved *recto-vesical operation,* and Dr. BOWEN's method of *depressing the cataract into the vitreous humour;* both of these publications we have already noticed at some length. Mr. Sleigh's operation has not been performed, we believe, upon the living subject; and we are not aware that Dr. Bowen's plan has yet been put to the test by any of our London oculists, though, from the success that has attended it in the numerous instances cited by Dr. Bowen, it is certainly highly deserving of a trial.

We now proceed to notice a few interesting surgical cases that have appeared within the last half-year; and we must observe, that a solitary instance of success in the treatment of a surgical disease is more worthy of our attention than any single medical case can be, because what the surgeon has once effected may be done again; the good produced is directly traceable to the means employed, and there is no room to suspect that the efforts of nature have performed a cure, which the physician ascribes to the action of some new or favourite remedy.

The first case we have to mention is recorded by Mr. MACLURE, of Glasgow, and in which that gentleman's perseverance, skill, and attention, are very conspicuous. The facts of the case were these:—A man who, from previous disease, *had lost the lower segment of the glans, and was afflicted with a fistulous opening in the perineum,* of many years' standing, applied to Mr. M. A bent probe could be introduced by the fistula backwards into the bladder; and a catheter or bougie might be introduced by the orifice of the urethra, and carried down to the obstructed part, which appeared to occupy about an inch and a half. The plan adopted for the removal of this obstruction was the use of the caustic bougie, which was persevered in for many months, through many circumstances of difficulty and discouragement, but finally with the result of re-establishing the integrity of the canal. The fistulous opening was not, however, closed; and the ulterior means which Mr. Maclure intended to have adopted for the cure of the fistula, were either the actual cautery, or denuding the sides of the sore by means of a cutting instrument, or the tagliacotian operation: circumstances, however, compelled the man to quit Glasgow, before either of these trials could be made.†

In the same work there is recorded a *case of sloughing of the bladder after parturition,* in which the patient was restored to health, and saved from the misery of a permanent fistula between the bladder and vagina, by the steady application of a tent composed of sponge, placed

---

* *Anderson's Quarterly Journal of the Medical Sciences,* No. 2.

† *Edinburgh Medical and Surgical Journal,* April 1824.

in the vagina as nearly as possible in contact with the perforation of the bladder, and a flexible catheter, fixed by suitable bandages, and passed into the bladder, where it was suffered constantly to remain.

We are aware that there is nothing very novel in the application of such obvious means of cure; but we mention this case, as well as the former one, with pleasure, because they evince great patience and steadiness of purpose on the part of the practitioner, and demonstrate how much good these solid qualities may effect, when under the guidance of a sound judgment. Little more than a month was sufficient to effect the cure of this fistula; and an examination, five months afterwards, proved that the patient remained perfectly free from her complaint, and had no inconvenience either in retaining or discharging her urine.

A nearly similar case is described by Dr. Cumin, of Glasgow, in the January Number of the same Journal. The result was equally fortunate, and the means employed were in effect the same.

In the Edinburgh Journal, we also find a history of *amputation*, performed for the cure of *tetanus traumaticus*. The operation was not successful; but Mr. Liston expresses his opinion that, had it been resorted to earlier, it would have been attended with a happier result. We do not think that general experience warrants this expectation; and, as far as our observation has extended, we have not found that amputation has been able to arrest the progress of this formidable disease.

A more than usual number of works relating to various practical points of surgery has been published within the last half-year: we shall notice the most important of these publications.

Mr. Syme and Mr. Liston both appear as advocates for *a new mode of performing the operation of amputation.** The former gentleman gives us rather an exaggerated picture of the usual mode of amputating by the circular incision; at least, we do not recognize the picture he has drawn as applicable to the practice of the metropolis. He recommends the adoption of the flap operation, not only in the leg but in other parts, and describes the method of performing it as follows:

"When the flap operation is performed, the soft parts may be cut either from without inwards, or from within outwards. The first of these methods was the one practised at the first introduction of the plan, and is still, I am informed, employed with great dexterity by that skilful surgeon Langenbeck; but the second is, I think, preferable, as it is done more easily, and seems to give less pain to the patient. In both cases, the object of the surgeon is precisely the same,—viz. the formation of proper flaps, by cutting more or less obliquely, according to the thickness of the soft parts. As I have said before, no general directions can be given for determining the extent of the incisions; I shall, therefore, content myself with describing the manner in which they may be executed most advantageously.

"Lisfranc recommends a long, very narrow knife, sharp on both

---

* *Edinburgh Medical and Surgical Journal*, January.

edges; but I think the one used by Mr. Liston better calculated for the purpose. It is about six inches in length and five-eighths in breadth, thin and blunt in the back, except for an inch from the point, which is very sharp; the back is straight, and so is the belly, except about an inch and a half from the point, which is slightly convex.

"The dimensions which I have stated are fully sufficient for the arm and fore-arm, the leg, and all amputations in children; for the thigh of an adult, a greater length will, of course, be required.

"The surgeon, grasping the limb with the left hand, at the place where it is to be removed, ascertains the situation of the bone by means of his thumb. He then introduces the knife over the bone at that part where he wishes to apply the saw, and, perpendicularly to it, he passes close by its side, and so on until the point appears directly opposite. This finishes the first part of the operation, or the transfixion, as it is called; after which he cuts his way outwards, in a line forming an angle with the bone, more or less acute, according to circumstances, so as to complete one flap.

"He then embraces the remaining undivided parts with his left hand, and, gathering them together, passes the knife on their side of the bone, so as to insulate it completely. Removing the restraint of his left hand, he now forms a second flap in the same way as he did the first.

"If there is only one bone, he immediately divides it, his assistant holding aside the flaps with his hands as high as it is exposed. If there are two, he separates the interosseous substance, and does the same thing; thus finishing his operation, which, with reasonable haste, and without the smallest hurry, need never occupy more than half a minute at most.

"It is impossible to imagine a greater contrast than that afforded by a comparison between the wound which results from this operation, and that caused by the method of circular incisions.

"The bone, instead of standing naked and conspicuous, can hardly be discovered until the flaps are laid aside. The muscles, not now deeply retracted, and exposing an abrupt ragged termination, extend far beyond the bone, and display two smooth equal surfaces, amply sufficient for coming into mutual contact, and well disposed for reciprocal union. Lastly, the skin, so far from forming a loose and hanging border about the wound, is left in undisturbed connexion with the subjacent parts, and in proportion just sufficient for supporting and covering them when the two flaps are brought together.

"The difference of appearance is not less remarkable after the wounds have been dressed.

"In the one case the line of union is straight, in the other it represents the arc of a circle;—in the first there is puckering of the integuments, in the second there is none;—in the former the muscles form projecting and inconvenient corners, in the latter they exist only when they are most required, i. e. over the bone."

In speaking of the two modifications of this operation by the circular incision, the one by M. Dupuytren, the other by Richter, Mr. Syme describes the former surgeon as cutting skin and muscles down to the bone at one sweep; and, after retracting the muscles to what he considers a sufficient extent, *in which he is not very particular*, he divides the bone, which he occasionally dresses round with dry charpie, previously to *approximating* the edges of the stump, for it is impossible to bring them into contact. Surely this detail does not entitle Mr. S. to call M. Dupuytren the ablest surgeon of the age we live in, although we are perfectly convinced that his merits are of a very high rank, and place him (some few antiquated prejudices excepted) upon a level with the first surgeons of this or any other country.

We cannot do more than say, that Mr. Syme's plan of amputating appears very plausible, though, perhaps, not free from objections; and that, with regard to the flap operation below the knee, as recommended by Mr. HEY, we know no good reason why it is generally disused, since the success attending it has usually been very complete, and the stump is much more firm and sound than that procured by a circular incision.

Mr. Liston's paper does little more than advocate the flap operation, as above described.

A short Memoir on *amputation at the shoulder-joint*, by LISFRANC, has been presented to us. The method of operating therein described is not essentially different from that mentioned by Mr. AVERILL, and we should not have noticed it but for two reasons: in the first place, to express our disgust at the silly vanity which leads a man so eminent as M. Lisfranc to say that this operation is one of the triumphs of French surgery, which formerly had no *rival*, and, perhaps, has none still. Willing as we are to give as much praise to French surgery as we can, —and in many respects it deserves very high commendation,—yet to say that it has no *rival*, is a stretch of absurdity that we are quite sure M. Roux (who has visited this country) would not have been guilty of. Our other reason is practical. M. Lisfranc agrees with M. Larrey in filling the wound, after this operation, with dry charpie; and adds, that that gentleman thinks, and *with reason*, that union by the first intention should not, in these cases, be attempted. What M. Larrey's reasons are, we are at a loss to conceive; but we should have been happy to have heard them.

One more remark upon another amputation now beginning to be in fashion, and we shall take leave of this subject: that remark is extracted from Mr. C. BELL's late work, of which an extended review has already been presented to our readers. At page 37, when speaking of necrosis of the thigh-bone consequent upon amputation, in which, the front of the stump falling into bad suppuration, the matter found its way into the medilulian of the bone, and necrosis followed.—" Even in such a case," says Mr. Bell, " if you found a bad stump and a projecting bone, and were to suppose that the whole bone was carious, and to conclude that, to amputate safely, you must take it off at the joint, you would commit one of those errors, for which it is a poor excuse to say that you knew no better." He pursues this subject in the following pages, which we seriously recommend to the perusal of the young surgeon, only lamenting that we have scarcely space for the following additional remark:—"Here is a bone," continues our able author, "which has been fractured by a musket-shot: the bone, you see, has united, but necrosis has been the remote consequence. Amputation has here been properly performed, since suppuration was wasting the patient; but was there a necessity for operating at the hip-joint?—You see there is no necrosis at the head or neck, or near the trochanter; and, if he had amputated even at the very centre of the bone, he could have withdrawn the sequestrum, and with it all source of irritation from the stump." We have seen this circumstance occur, highly to the credit of the surgeon who amputated; and we make no doubt that, had this gentleman been seduced by the temptation of performing the hip-joint operation, that the death of the patient must have been the result.

M. Lisfranc has published an account of *a new application of the stethoscope to the detection of fractures*, and which he affirms renders them easily detectable, even under circumstances of great swelling, and with a very slight motion of the fractured parts. We shall insert the general rules which this gentleman lays down for the application of this instrument.

"1. When the stethoscope is applied upon a fracture, it is almost indifferent whether the mouth-piece be left in it or not; but in proportion as we recede from it, the crepitation becomes more sensible, if the instrument be deprived of the mouth-piece.

"The more superficial the bones are, the more the crepitation is distinct; and, as the slightest movements are sufficient to produce it, it is the most sensible upon the fracture. Thus we not only determine the fact of the fracture, but also its precise seat. It would be useless to remark how much we thus avoid those painful examinations, and how important it is in practice to know the precise situation of the solution of continuity in the bone. To discover this situation with accuracy, it is indispensable that the movements impressed upon the fragments should be always the same.

"3. The crepitation is less appreciable in proportion as we recede from the fractured point, but it is heard at almost inconceivable distances: in that case, however, it must be rather strong.

"4. When a fracture exists with crossing of the part *(chevauchement)*, the crepitation is less easy to be distinguished; but, if an unexercised ear do not perceive it very easily, it may be rendered much more distinct by practising slight extension and counter extension.

"5. The crepitation occasioned by fragments of compact bones produces an acute sound, with loud crackling *(des forts petillemens)*: perceived by the stethoscope, they are often loud, and sometimes considerably offend the ear.

"6. The crepitation of fragments of spongy bones is obtuse *(sourde)*, and is similar to the action of a file upon a hard and porous body (the pumice-stone for instance); the noise, from time to time, is intermixed with rather stronger sounds, which have a certain analogy with the crepitation of compact bones.

"7. The crepitation of oblique fractures is stronger than that of transverse fractures.

"8. If a liquid be effused round the fragments, there is joined to the crepitation a noise similar to that which the foot produces in a bad shoe containing water.

"9. When the fracture is complicated with splinters, there is heard, besides the ordinary crepitation, a sort of crackling, similar to that which many hard and angular bodies would produce when rubbed against each other.

"10. When the fracture is accompanied by wounds of the soft parts, to the crepitation are joined sounds similar to those produced by strong inspirations and expirations, when the mouth is widely open.

"11. Luxations cannot be confounded with fractures; for the sensation produced by the displaced articular surfaces is slight, and hardly extends beyond the situation of displacement: it is obtuse, like that of two polished and humid surfaces moved upon each other.

"12. The movement of tendons in their sheaths produces sounds, full, obtuse, jerking, rather thin *(rares)*, and extremely different from crepitation."*

We shall do no more upon this occasion than allude to Mr. LISTON's remarks on *caries of the bones*, because his paper is at present incomplete: our next Retrospect will, therefore, embrace the whole of that obscure subject.

On *spinal diseases* a great deal has been written during the last half-year, and the treatment of the lateral curvature appears now to be placed upon a much firmer and more philosophical basis than formerly. Mr. SHAW's work must justly be quoted as a very valuable addition to our stock of knowledge in this class of complaints; and we also mention, with much satisfaction, that Mr. BAMPFIELD has lately received the Fothergillian medal of the Medical Society for his Essay on Spinal Curvatures, the substance of which appeared in the pages of this Journal some months ago.

On the subject of *syphilis* we have not much novelty to record. Many pages of the Journal Complementaire have, for two or three months, been occupied by the opinions of M. SIMON on the treatment of this disease: he is a violent advocate for salivation, and full of the old prejudices relating to the disease itself.

Mr. BOYLE has lately published a small volume, the object of which is to recommend large doses of the submuriate of mercury for the cure of the primary symptoms of this disease. This practice is not different from that in vogue in the days of WISEMAN, and more lately of TURNER. For our own parts, we conceive the question as to the propriety of administering mercury in this or that particular case, to be of infinitely more importance than a choice of the form of preparation employed. This we have no doubt about, that, among the poor and the labouring classes in great towns, the profuse exhibition of mercury is more fraught with evil, than one unacquainted with the cases usually

* *Anderson's Quarterly Journal*, No. 2.

met with at the public charities of the metropolis could suppose. Of Mr. Boyle's work we do not wish to speak unfavourably; but we cannot help thinking that it exhibits but a slight acquaintance with the real merits of the mercurial plan of treatment, so freely tried in this country some years ago; and that the facts brought to light by that investigation have either escaped his notice, or not made a due impression upon his mind.

Dr. BALINGALL* has published some remarks on *the carious skull of a person who died of syphilis;* and, since he has never been able to find that mercury has produced carious bones, or other secondary symptoms, in those who have taken that remedy freely for the cure of liver-complaints or other diseases, he concludes that syphilis, and not mercury, is the cause of caries. Now, we think a different explanation might be given of this matter. In the first place, is it not possible that there may be some *relation* between the disease (syphilis) and the remedy that calls into action the inflammation and suppuration of the bone? Has Dr. Balingall carefully examined all those cases of supposed syphilitic caries, whether of the bones of the nose, tibia, or cranium, that come before him? and, if so, has he not *invariably* found that these sufferers have *all* taken mercury freely, under circumstances of exposure to weather, or evident derangement of health? Anxious as we are to establish the truth in this important question, and having made it our business to inquire closely into it, we have found hitherto strong reasons for believing that mercury, pushed too far, or injudiciously administered, either with regard to time or situation, has always been the exciting cause of affections of the bones themselves. For example, may not syphilis excite a scrofulous affection of the periosteum and bone? and will not mercury, administered freely, under the supposition of this affection being venereal, inevitably produce inflammation and caries of the bone itself?—in which case, though the original taint be venereal, the diseased condition of the bone must undoubtedly be ascribed to the rash and intemperate administration of this remedy.

We have also seen two or three well-marked cases of *eruption*, *sore throat*, and *pains of the limbs*, consecutive upon the liberal exhibition of this remedy in diseases of the liver; and one remarkable case, where a severe *ptyalism* recurred, without any apparent cause, after a cessation of more than three months.

Mr. MACILWAIN has published a small work upon the treatment of *strictures of the urethra*, which we strongly recommend as containing sound views, and highly valuable practical rules of conduct. We shall endeavour to make our readers better acquainted with the contents of this little work, in a subsequent Number of our Journal; but we can truly say that such a publication was much wanted, and that want is now very satisfactorily supplied.

We have looked over M. OLLIVIER's work on *diseases of the spinal*

---

* *Edinburgh Medico-Chirurgical Transactions.*

*marrow,* but we do not find any surgical novelties to present to our readers.

A great number of valuable cases of *pressure produced by fracture and other injuries of the spine* are related; but, neither theoretically nor practically, is there any thing added to the present stock of our knowledge, or any improvement suggested as to their mode of treatment.

The *lateral operation for lithotomy* has lately found a very able advocate in Mr. KEY, whose Treatise is highly deserving of an attentive perusal. The object of Mr. Key's work is to recommend the substitution of a director, which is straight, with the exception of a slight curve near the extremity, and a knife, which is longer than a common scalpel, and slightly convex in the back near the point. The mode of performing the operation with these instruments he thus describes:—

"An assistant holding the director, with the handle somewhat inclined towards the operator, the external incision, of the usual extent, is made with the knife, until the groove is opened, and the point of the knife rests fairly in the director, which can be readily ascertained by the sensation communicated; the point being kept steadily against the groove, the operator with his left hand takes the handle of the director, and lowers it till he brings the handle to the elevation described in Plate III. keeping his right hand fixed; then, with an easy simultaneous movement of both hands, the groove of the director and the edge of the knife are to be turned obliquely towards the patient's left side. The knife, having the proper bearing, is now ready for the section of the prostate: at this time the operator should look to the exact line the director takes, in order to carry the knife safely and slowly along the groove; which may now be done without any risk of the point slipping out. The knife may then be either withdrawn along the director, or the parts further dilated, according to the circumstances I have adverted to. Having delivered his knife to the assistant, the operator takes the staff in his right hand, and passing the fore finger of his left along the director through the opening in the prostate, withdraws the director, and exchanging it for the forceps, passes the latter upon his finger into the cavity of the bladder.

"In extracting the calculus, should the aperture in the prostate prove too small, and a great degree of violence be required to make it pass through the opening, it is advisable always to dilate with the knife, rather than expose the patient to the inevitable danger consequent upon laceration."

We perfectly accord with Mr. Key in his objections to the gorget; and his observations upon the disadvantages of the staff are very strong, and it certainly requires much practice and considerable *tact* to manage it: yet the success, both of Mr. MARTINEAU and Mr. DALRYMPLE at Norwich, are as great as can reasonably be expected under any mode of operating that can be suggested. We know not why the *lithotome caché* has been so entirely banished from practice in this country. We have seen it frequently employed on the continent with the greatest success. Upon the whole, we are induced to think that the lateral operation performed with the knife and staff, or with the director, as recommended by Mr. Key, comes as nearly to perfection as possible.

One more point, we conceive, this gentleman has explained very properly,—that is, the cause of death after this operation; which, he says, arises generally from suppuration of the reticular texture surrounding the bladder. This serious disease, so often consequent upon wounds and punctures, is frequently overlooked, and has never been explained and illustrated in the manner it deserves.

We have now gone through our catalogue of the most important new publications, and nothing remains for us to notice but the new instruments and machines, which have been lately added to our surgical armoury. This is, however, the most difficult part of our task; since, to describe mechanical contrivances without the aid of engravings, is by no means easy.

Mr. ELDERTON has described, in the Edinburgh Journal, *a new fracture box*, intended to afford permanent and complete quietude in the treatment of compound fractures. This box is constructed to admit either of the horizontal posture, or of any degree of inclination that may be preferred; in which, however, it does not differ from other contrivances of the same kind. Its peculiar feature is, that, on the upper side of the *floor*, as it may be called, twenty transverse grooves are cut, an inch apart from each other, and half an inch deep, for the purpose of receiving a corresponding number of dove-tailed wooden slides. Each slide, which is five inches long, has a hole bored on its upper edge, half an inch in diameter and one inch deep, for the purpose of receiving the rounded tenon of the several perpendicular wooden pins, each five inches long. At the bottom of each transverse groove are bored small holes, a quarter of an inch apart, to receive iron pins and to support the slides; and which, as well as the wooden pegs above mentioned, are driven more or less inwards, to suit the different diameters of the fractured leg. There are also some peculiarities of contrivance respecting the foot-board, which, for want of the engraving, we cannot enter into. The advantage of the above contrivance consists in the surgeon being enabled to dress the wound, or to examine any part of the limb, without subjecting it to motion.

We have two contrivances presented to our notice, for the purpose of *tying deep-seated arteries*. We conceive that Mr. WEISS's aneurismal needle supersedes the necessity, almost, of recording any invention for that purpose; but it is our duty to mention every thing that comes before us, having a claim to novelty. One of these instruments is invented by Professor JACOBSON, of Copenhagen; the other by M. PREVOST, of Geneva: they are both too complex for mere description, and we can do no more than refer to the plate.*

We have already mentioned† Mr. WEISS's *new dilator for the anus or vagina*, upon which occasion we remarked the close resemblance which it bears to an instrument destined for the same purpose, described

---

* *Edinburgh Medical and Surgical Journal*, April 1824.

† *London Medical and Physical Journal*, May.

by AMBROSE PARÉ. We say this without meaning to detract from Mr. Weiss's merit, since we have no doubt that, as far as he is concerned, the invention is entirely his own; and we cannot too much praise the liberality which that gentleman has shown in putting his invention so unreservedly before the profession.

---

As the imperfections of any production are in some degree measured by its pretensions, we request our readers to observe, that, in the foregoing Paper, we do not profess to have given a complete history of the Medical Sciences during the last six months; but merely a compilation of the most important facts which have come to our knowledge within this period, *and which we have not previously had an opportunity of mentioning*. We adopt this plan to avoid unnecessary recapitulation; and, on this account, we decline noticing either the valuable original communications published in the preceding volume, or any of the works which we have already reviewed.

---

## Figures and Tables

**Figure f1:**
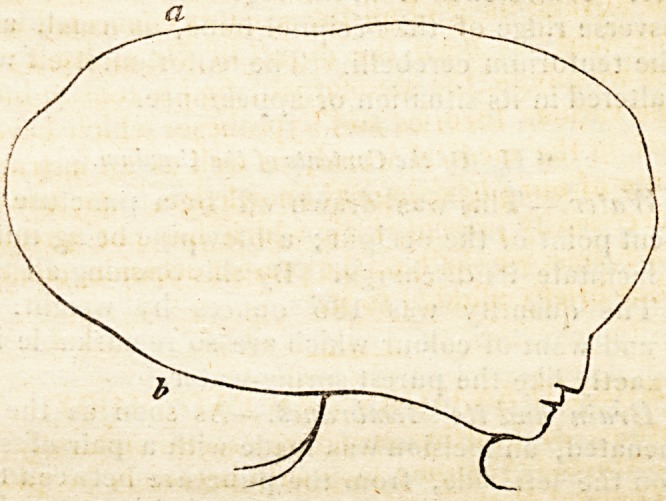


**Figure f2:**
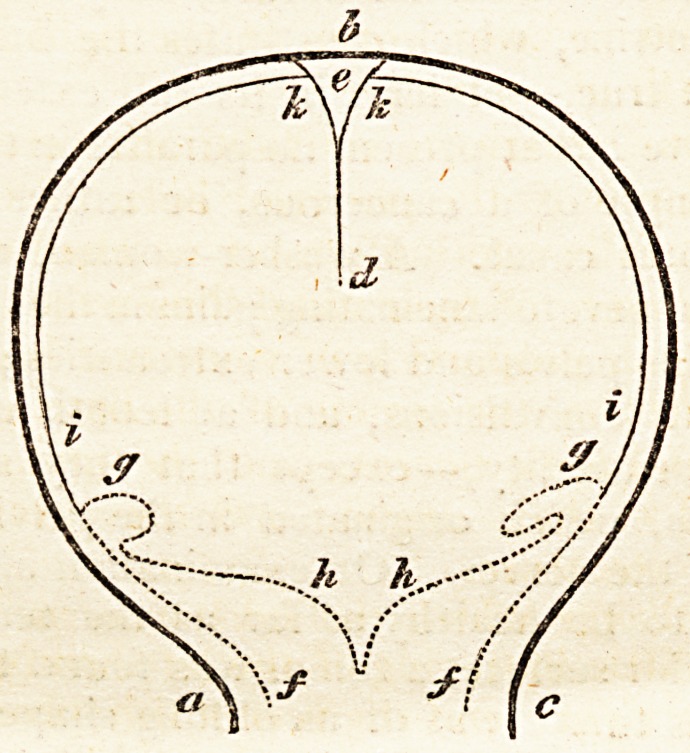


**Figure f3:**